# Supplementary material for: Changes in management of owned cats in the countryside – A comparison of results from surveys undertaken in the same rural area of Denmark in 1998 and 2022
Source: PLoS One. 2025 Feb 19;20(2):e0316704. doi: 10.1371/journal.pone.0316704 (PMC11838870; doi:10.1371/journal.pone.0316704)
Supplement: S1 File — (PDF) [file pone.0316704.s001.pdf]

Projekt "Landkat"

DYRENES BESKYTTELSE

Titel: Projekt ”Landkat”

Udarbejdet af: Marianne Ujvári

Styringsgruppe: Hans Baagøe (Zoologisk Museum), Bjarne Clausen (Dyrenes Beskyttelse) & Bengt Holst (Københavns Zoo)

Udgivelsestidspunkt: Juli 1999

Udgiver: Dyrenes Beskyttelse
Alhambravej 15
1826 Frederiksberg C

Med støtte fra: Aage V. Jensens Fonde

Forside foto: Klaus Bentzen/BIOFOTO

Oplag: 100

MÅ FØRST OFFENTLIGGØRES EFTER 1. JANUAR 2000.

Kan rekvireres hos: Dyrenes Beskyttelse
Alhambravej 15
1826 Frederiksberg C
Tlf. 33 22 32 22

INDHOLDSFORTEGNELSE

Forord s. 4
Indledning s. 5
Formål s. 7
Metode s. 8
Dataindsamling s. 8
Databehandling s. 10
Definitioner s. 10
Resultater s. 13
Antallet af katte s. 13
Beboernes holdning til antallet af katte i forsøgsområdet s. 17
Formålet med katteholdet s. 18
Kattenes pasning og sundhed s. 19
Kattenes reproduktion og bestandstilvækst s. 29
Diskussion s. 31
Antallet af katte s. 31
Kattenes reproduktion og bestandstilvækst s. 34
Kattene og faunaen s. 35
Kattene og mennesket s. 36
Kattenes pasning og sundhed – dyreværnsmæssige aspekter s. 37
Anbefalinger til Dyrenes Beskyttelse s. 42
Hvad mangler vi at få belyst og hvordan kan det gøres s. 43
Konklusion s. 45
Referencer s. 47

FORORD

Denne undersøgelse blev iværksat af Dyrenes Beskyttelse med støtte fra Aage V. Jensens Fonde. Undersøgelsen har kun været mulig i kraft af stor interesse, blandt beboerne i forsøgsområdet, i at besvare mine spørgsmål.

Undervejs i forløbet med at indsamle data og udfærdige nærværende rapport, har jeg fået værdifuld støtte og konstruktiv kritik af min styringsgruppe, bestående af lektor, Lic. scient., Hans J. Baagøe; vicedirektør, Cand. scient., Bengt Holst samt Cand. med. vet. Bjarne Clausen.

Endvidere ønsker jeg at rette en tak til Lic. scient. Tommy Asferg, Danmarks Miljø Undersøgelser, Kalø, for gode kommentarer og kritik.

Jeg håber at rapporten vil bidrage til en større forståelse af tamkattens (*Felis catus)* levevilkår i landområder i Danmark.

INDLEDNING

Tamkatten (*Felis catus*) er et meget almindeligt husdyr i Danmark, og den bliver tilsyneladende stadig mere populær. Ifølge Dansk Media Indeks steg andelen af husstande med kat fra 18 % til 22 % i perioden 1987 til 1995, mens andelen af husstande med hund faldt fra 30 % til 27 %. Katten udfordrer altså alvorligt hunden som det mest populære familiedyr. På trods af det nære forhold vi i dag har til katten, ved vi imidlertid kun lidt om dens adfærd og økologi. Katten er interessant set ud fra flere synspunkter, idet katten på flere områder adskiller sig fra andre danske husdyr.

Katten er et domesticeret dyr, men den lever et dobbeltliv – dels er den tæt tilknyttet mennesket, dels kan den bevæge sig frit omkring i naturen. Enkelte undtagelser er dog de deciderede indekatte, der holdes i lejligheder, huse og SPF stalde. Denne specielle livsstil som domesticeret og dog fritlevende, betyder at katten i modsætning til andre husdyr direkte påvirker faunaen. Denne kendsgerning gør katten interessant set ud fra et økologisk synspunkt. Men katten fodres også ofte af mennesket. Den har således mennesket at falde tilbage på i perioder med mangel på naturlige fødeemner. Derfor reguleres bestanden af katte ikke af mængden af deres byttedyr, som det er tilfældet for andre predatorer. Derved holdes bestanden af katte på et kunstigt højt niveau (Liberg & Sandell 1988).

Katten "skiller vandene" i dem, der er for, og dem, der er imod katten. Kattens antal og jagtinteresse bevirker, at den fra flere sider betragtes som skadevoldere. Dels af naturinteresserede, der ønsker så divers og talrig en fauna som muligt, dels af jægere der ofte opfatter katten som en konkurrent. Samtidig drager mennesket dog også stor nytte af katten, som vi igennem tiden har brugt som muse- og rottefanger. Desuden er det gennem de seneste år erkendt, at katten, såvel som andre familiedyr, har stor social – og mentalhygiejnisk værdi. Familiedyr stimulerer evnen til nonverbal kommunikation, tilbyder uforbeholden og betingelsesløs kærlighed samt har en beroligende virkning på de fleste mennesker, og virker derved angstnedsættende (Madsen 1988).

Dyrenes Beskyttelses udgifter til herreløse katte, fortrinsvis transport, aflivning, neutralisering og genudsætning af katte, steg fra kr. 598.056 i 1993 til kr. 1.177.540 i 1996

(ekskl. kattenes andel af foreningens udgifter til Falck i forbindelse med hjælp til tilskadekomne dyr) (Dorte Prytz Jensen, pers. comm.). I 1997 faldt udgifterne til de herreløse katte til kr. 542.851, hvilket sandsynligvis skyldes en holdningsændring i foreningen der betyder, at foreningen ikke længere betaler aflivningen af raske katte hvor herreløse disse end måtte være. Desuden blev der i 1997 iværksat en afvikling af fodérværtsordningen. En ordning hvor en person påtager sig at fodre herreløse katte, mod at Dyrenes Beskyttelse refunderer udgifterne. De store udgifter, til forvaltning af katte, belaster Dyrenes Beskyttelses samlede budget. Samtidig påpegede visse af foreningens udvalgsmedlemmer det dobbeltmorale i, på den ene side, at fodre, og på den anden side, at begrænse antallet af katte. Derfor opstod der et ønske om at indsamle viden om katten. Viden der vil lette forvaltningen af katteproblemerne, ved f.eks. at foreslå forebyggende aktiviteter, og således på længere sigt nedsætte foreningens udgifter til nødstedte, herreløse katte.

Et muligt rekrutteringsområde for herreløse katte er landet. Dels fordi såvel ejerkatte som herreløse katte på landet har udstrakt mulighed for at strejfe og derved blive betragtet som herreløse. Dels fordi afkom, af såvel ejerkatte som herreløse katte på landet, kan ende som herreløse katte. Da den nuværende viden om katte på landet og deres forhold til mennesker er begrænset, er det imidlertid vigtigt at frembringe mere viden om netop denne gruppe katte. Hvor mange katte er der på landet? Hvor mange killinger får kattene pr. kuld? Bekymrer landboerne sig om deres katte? Dét er nogle af de spørgsmål, der vil blive forsøgt besvaret i nærværende undersøgelse.

FORMÅL

Formålet med undersøgelsen er at:
Beregne antallet af katte med fast tilknytning til en husstand i et givent område på landet i Danmark. Undersøge om ejendommens karakter påvirker antallet af katte på ejendommen. Estimere det samlede antal katte på landet i Danmark med fast tilknytning til en husstand.

Belyse reproduktion hos katte med fast tilknytning til en husstand, bl.a. kuldstørrelse og tidspunkt på året. Komme med et bud på bestandstilvæksten i området udfra kuldstørrelsen og antallet af katte i området.

Belyse forholdet mellem katte og mennesker, dvs. formålet med katteholdet og hvorvidt beboerne føler der er for mange katte i området.

Belyse kattenes tilknytning til mennesker, dvs. pasning samt sundhed, og eventuelle dyreværnsmæssige problemer ved denne tilknytning eller mangel på samme.

METODE

Dataindsamling

Undersøgelsen var opbygget som en spørgeskemaundersøgelse (bilag 1), der blev udført som et besøgsinterview. Interviewet fandt sted i perioden 5. januar til 3. april 1998, i et 47 km^2 stort område nord for Ringsted på Midtsjælland (fig. 1). I området findes en varieret bebyggelse og en varieret arealanvendelse og driftsform. Området blev udvalgt ud fra to kriterier. Det skulle dels være så repræsentativt for landområder i Danmark som muligt, mht. bebyggelse, arealanvendelse og driftsform, dels ligge i kørselsafstand fra København.

Besøgsinterviewet blev i praksis udført ved, at en interviewer kørte rundt i bil og udspurgte beboerne og noterede på spørgeskemaet. Ugen før besøget meddeltes interviewerens ankomst via et postomdelt brev adresseret til beboeren (bilag 2). Det var ikke muligt at angive præcis dato og tidspunkt for besøget, men kun uge. Såfremt der ikke blev truffet nogen hjemme, blev der lagt en besked om, at intervieweren havde været der og ville komme igen på et nærmere fastlagt tidspunkt (bilag 3). For at undgå påvirkninger af svarpersonerne repræsenterede intervieweren den neutrale institution, Zoologisk Museum, under besøget, ikke interesseorganisationen Dyrenes Beskyttelse.

I nogle tilfælde formulerede hele familier svarene på interviewerens spørgsmål, i andre tilfælde formulerede kun én person svarene. Dette kan være en ulempe, idet data derved ikke er helt sammenlignelige. Et spørgeskema udfyldt på baggrund af interview af flere personer, vil nemlig ofte være et kompromis af de enkelte personers meninger. Der var imidlertid ikke noget alternativ, der ville give lige så høj svarprocent. På ejendomme hvor der ikke blev truffet mindst én beboer, ældre end 14 år, hjemme på besøgstidspunktet, blev interviewet udsat til en anden dag.

For at øge antallet af adspurgte fuldtidshusdyrbrug, blev 23 ejendomme, beliggende udenfor forsøgsområdet, udvalgt v.h.a. bogen "Større gårde og skove 88/89" (Anonym 1989). De første 23 landbrug i bogen, der levede op til forudsætningerne om at have husdyr og ligge i en afstand af max. 14 km fra forsøgsområdet, blev valgt. Der blev

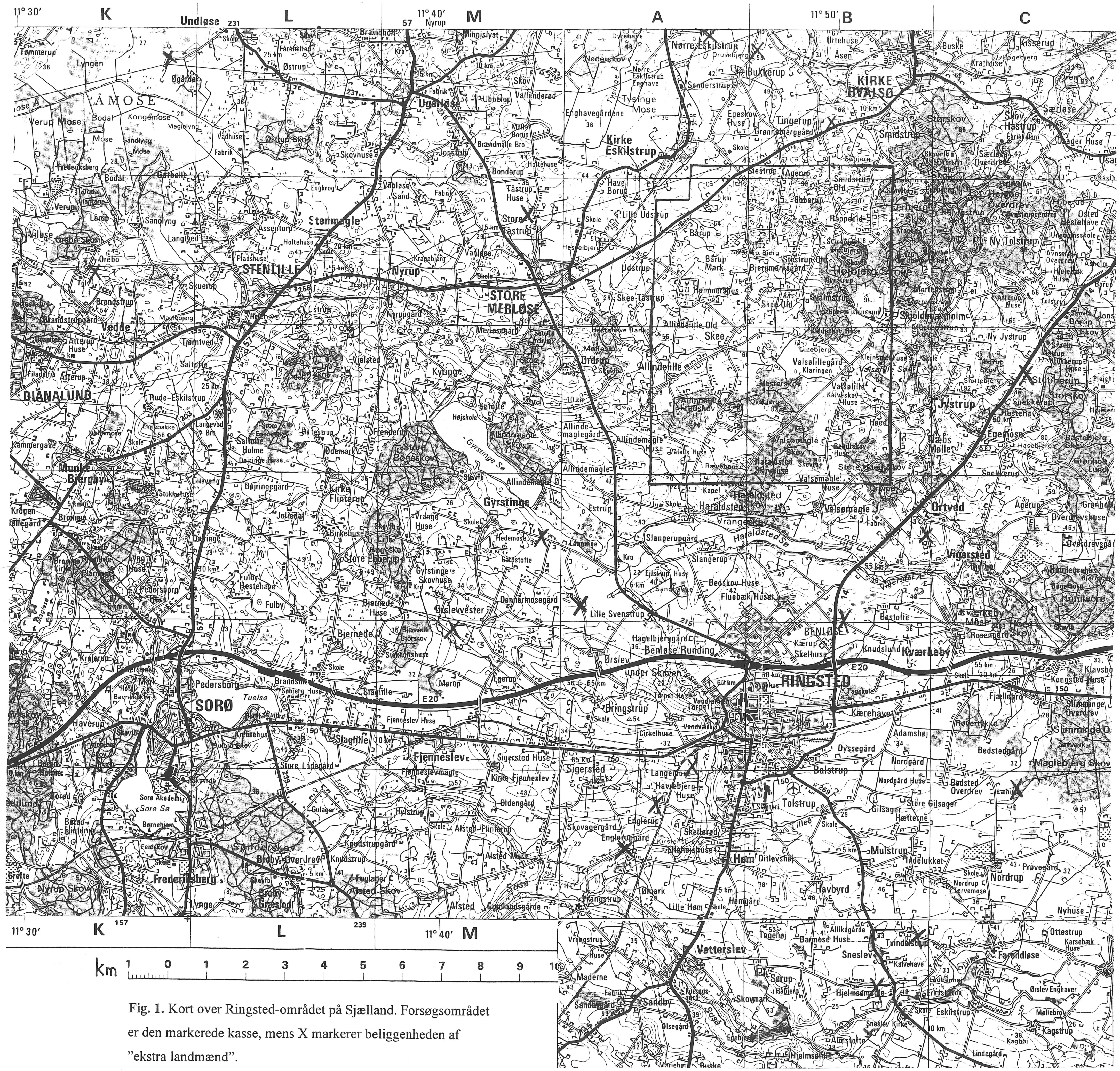

Fig. 1. Kort over Ringsted-området på Sjælland. Forsøgsområdet er den markerede kasse, mens X markerer beliggenheden af "ekstra landmænd".

sendt et brev, meddelende interviewerens ankomst til disse landbrug, 1 uge før den dag besøget skulle finde sted. Det lykkedes at interviewe beboerne på 18 af disse 23 landbrug d. 28. maj (fig. 1). Beboerne på disse landbrug (fremover kaldet "ekstra landbrug") blev kun spurgt om den del af spørgeskemaet, der vedrører ejendommen og antallet af katte på ejendommen. Besvarelserne fra disse landbrug blev kun inddraget i afsnittene "Antallet og fordelingen af kattene på ejendommene" og "Antallet af katte på landet i Danmark – et estimat".

Databehandling

Alle besvarelser blev lagt i databasen Access. Til at sammenligne to stikprøvers gennemsnit bruges en Mann-Whitney test. Er der flere end to stikprøver bruges en Kruskal-Wallis test. Til at teste om udfaldet af fordelingen af data i de forskellige grupper er tilfældig bruges χ^2. Der valgtes et niveau for signifikans på 0,05, og et niveau for tendens på 0,10.

Definitioner

I spørgeskemaet og nærværende rapport er følgende definitioner anvendt. Definitioner af ejendomstyper tager udgangspunkt i Danmarks Statistiks definitioner, men er tilpasset behovet i nærværende undersøgelse.

Parcelhus: Alle hustyper (villa, parcelhus, rækkehus) der ikke har mere jord, end hvad må betragtes som have. Dvs. der ejes ikke landbrugsjord og drives ikke landbrug fra/på ejendommen. Opdeles i enkeltliggende parcelhus, parcelhus i klynge og parcelhus i bymæssig bebyggelse.

Enkeltliggende parcelhus: Op til 3 parcelhuse, hvor afstanden mellem husene to og to er mindre end 200 meter, som er beliggende mere end 200 meter fra øvrige parcelhuse.

Mindre klynge parcelhuse: En sammenhængende bebyggelse med 4-10 huse; at bebyggelsen er sammenhængende vil sige, at afstanden mellem husene ikke overstiger 200 meter, medmindre afbrydelsen skyldes offentlige anlæg, kirkegårde o.lign.

Parcelhus i bymæssig bebyggelse: En sammenhængende bebyggelse med 11 huse eller flere; at bebyggelsen er sammenhængende vil sige, at afstanden mellem husene ikke overstiger 200 meter, medmindre afbrydelsen skyldes offentlige anlæg, kirkegårde o.lign.

Fritidslandbrug: Beboelser, uanset beliggenheden i forhold til andre boliger (bymæssig bebyggelse eller fritliggende), hvortil der er knyttet landbrugsjord eller jord, der bruges til fritidslandbrug, og altså ekskl. beboelser hvortil der blot findes almindelige haver. Drives på fritids- eller deltids-basis. Tilforpagtede og tillejede arealer medregnes ikke under bedriften, mens bortforpagtede eller bortlejede arealer betragtes som hørende til bedriften. Opdeles i fritidsagerbrug og fritidshusdyrbrug.

Fritidsagerbrug: Landbrug hvor agerbrug, braklægning, gartneri, permanente beplantninger, anden planteavl og blandet planteavl udgør hele brugets driftsform. Der kan holdes husdyr til landbofamiliens eget forbrug (ernæring og forlystelse), men aldrig i erhvervsøjemed. Holdet af husdyr til ernæring og forlystelse skønnedes ikke at udgøre en væsentlig del af landbruget, f.eks. optage størstedelen af jorden.

Fritidshusdyrbrug: Landbrug hvor kødkvæg, malkekvæg, svin, fjerkræ, minkavl, hestehold og/eller blandet husdyrhold udgør brugets driftsform eller skønnedes at udgøre en reel del heraf. Inkluderer de brug hvor husdyrene holdes i erhvervsøjemed eller til landbofamiliens eget forbrug, og hvor husdyrene skønnedes at udgøre en væsentlig del af landbruget, f.eks. ved at optage størstedelen af jorden.

Fuldtidslandbrug: Beboelser, uanset beliggenheden i forhold til andre boliger (bymæssig bebyggelse eller fritliggende), hvortil der er knyttet landbrugsjord, dvs. ekskl. beboelser hvortil der blot findes almindelige haver. Drives på fuldtids-basis. Tilforpagtede og tillejede arealer medregnes ikke under bedriften, mens bortforpagtede eller bortlejede arealer betragtes som hørende til bedriften. Opdeles i fuldtidsagerbrug og fuldtidshusdyrbrug, se fritidslandbrug for definition.

Kattene fodres: Katten(e) tilbydes foder dagligt.

Huskat: Katte der kommer indenfor i boligen. Katte som blot kommer indenfor i køkken, bryggers el.lign. sted i forbindelse med fodring er ikke inkluderet. Opdeles i familiekat og predatorkat.

Staldkat: Katte der ikke kommer indenfor i boligen, udover køkkenet, bryggers eller lign. i forbindelse med fodring. Opdeles i familiekat og predatorkat.

Familiekat: Katte holdt med det primære formål at være en del af familien, et kæledyr.

Predatorkat: Katte holdt med det primære formål at tage mus og rotter.

Killinger: Definitionen af killinger er ikke konsekvent, men er forskellig for spørgsmålene vedr. sygdom og aflivning. Aflivning: Killinger defineret af svarpersonen selv, dvs. det varierer fra individer, der endnu ikke har fået øjne, til individer, som er mindre afhængige af moderen. Sygdom: Ved killinger forstås katte i 0-7 måneders alderen begge måneder inklusive. Ved voksne katte forstås alle katte 8 måneder eller ældre .

Katten vaccineres: Katten bliver vaccineret jævnligt, dvs. er blevet vaccineret gentagne gange. Katte som kun er vaccineret én gang inkluderes ikke.

Årstider: Forår: marts, april, maj. Sommer: juni, juli, august. Efterår: september, oktober, november. Vinter: december, januar, februar

RESULTATER

Antallet af katte

Antallet af katte i forsøgsområdet

I det 47 km^2 store forsøgsområde var der i alt 488 ejendomme. Der blev truffet folk hjemme på 451 ejendomme svarende til 92,4 % af samtlige ejendomme i området. 269 af de 451 udspurgte havde kat(te), hvilket svarer til at 59,6 % af alle udspurgte havde kat(te).

På de 451 ejendomme, hvor rundspørgen blev foretaget, var der på besøgstidspunktet med sikkerhed 780 katte. Indenfor de sidste 12 måneder havde der, ifølge beboerne, været 920 katte i området. 140 katte var altså forsvundet fra området i det forgangne år. På 14 ejendomme var der usikkerhed om antallet af katte, og der kan have været yderligere 21 katte på disse ejendomme. Dermed var det samlede antal katte i området, indenfor de sidste 12 måneder, 941 katte. Bestandstætheden i området var således 16,6 – 20,0 katte pr. km^2. Beboerne på 269 ejendomme havde på besøgstidspunktet med sikkerhed én eller flere katte, dermed var der i gennemsnit 2,9 ± 2,6 (SD) katte pr. ejendom med kat(te). I de følgende belyste sammenhænge, er kun de katte taget i betragtning, som med sikkerhed var der på besøgstidspunktet.

Antallet og fordelingen af kattene på ejendommene (inkl. "ekstra landbrug")

I tab. 1 ses det gennemsnitlige antal katte pr. ejendom. Der er signifikant forskel på de 7 ejendomstyper mht. antal katte pr. ejendom ($p < 0,0001$). Der var ikke signifikant forskel på det gennemsnitlige antal katte på parcelhuse i klynge og hhv. enkeltliggende parcelhuse ($p = 0,4216$) og parcelhuse i bymæssig bebyggelse ($p = 0,2043$). Derimod var der signifikant flere katte på enkeltliggende parcelhuse end på parcelhuse i bymæssig bebyggelse ($p = 0,0062$). Samtlige tre typer parcelhuse havde i gennemsnit signifikant færre katte pr. ejendom end de fire typer landbrugsejendomme (PEn: FrA, $p = 0,0489$; FrH, $p < 0,0001$; FuA, $p = 0,0007$; FuH, $p < 0,0001$. PKl: FrA, $p = 0,0320$; FrH, $p < 0,0001$; FuH, $p = 0,0012$; FuA, $p < 0,0001$. PBy: FrA, $p = 0,0002$; FrH, $p < 0,0001$; FuA, $p < 0,0001$, FuH, $p < 0,0001$).

Husdyrbrug, uanset fuldtids- eller fritidsbasis, havde i gennemsnit signifikant flere katte pr. ejendom end fritidsagerbrug (hhv. $p = 0,0004$ og $p = 0,0007$). Der var ikke signifikant forskel på det gennemsnitlige antal katte på fuldtidsagerbrug og fritidshusdyrbrug ($p = 0,3004$) samt på fuldtidshusdyrbrug og fritidshusdyrbrug ($p = 0,2480$). Men der er tendens til at fuldtidshusdyrbrugene i gennemsnit havde flere katte pr. ejendom end fuldtidsagerbrugene ($p = 0,0708$) og til at fuldtidsagerbrug havde flere katte end fritidsagerbrug ($p = 0,0984$). Med 5,03 ± 5,62 (SD) katte i gennemsnit pr. fuldtidshusdyrbrug havde disse således flere katte pr. ejendom end alle andre typer ejendomme på nær fritidshusdyrbrug.

Tabel 1. Gennemsnitlig antal katte per ejendom.

Ejendomstype	Antal katte per ejendom i gennemsnit [a]	n [b]	Standardafvigelse
Parcelhus – enkeltliggende	1,16	161	1,63
Parcelhus – klynge	0,75	32	0,92
Parcelhus – bymæssig bebyggelse	0,54	65	0,90
Fritidsagerbrug	1,70	61	1,91
Fritidshusdyrbrug	3,07	69	2,72
Fuldtidsagerbrug	2,75	40	2,92
Fuldtidshusdyrbrug	5,03	39	5,62

[a] inkl. ejendomme med 0 katte og "ekstra landbrug".

[b] det totale antal ejendomme (467) fremkommer som følge af de 451 ejendomme i forsøgsområdet, minus 2 ejendomme med mangelfulde oplysninger om ejendommen, samt de 18 "ekstra landbrug".

Fordelingen af kattene på ejendommene ses i figur 2. I nærværende undersøgelse har 14 (36 %) fuldtidshusdyrbrug, 7 (18 %) fuldtidsagerbrug, 16 (23 %) fritidshusdyrbrug, 6 (10 %) fritidsagerbrug og 10 (6 %) enkeltliggende parcelhuse 5 katte eller flere. Der er signifikant forskel på fordelingen af ejendomme med hhv. 1-4 katte og 5 eller flere katte på de 7 ejendomstyper ($p < 0,0001$; PKl og PBy slået sammen).

Der er gennemsnitlig lige mange katte på arealmæssigt store fritidshusdyrbrug (> 5 ha) og på små fritidshusdyrbrug (≤ 5 ha), nemlig hhv. 3,1 ± 2,2 katte (n = 37) og 3,1 ± 3,2 (n

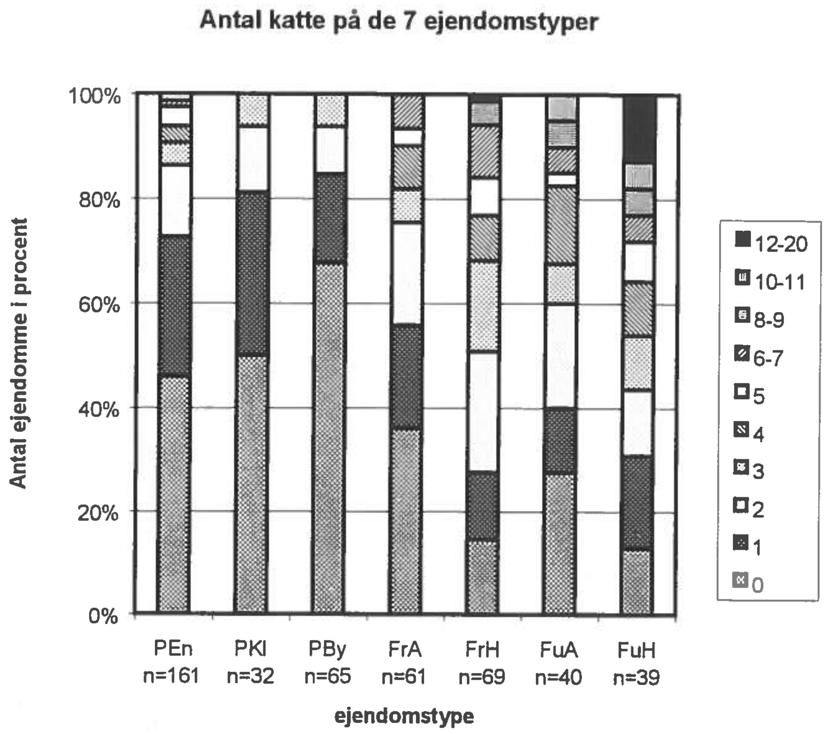

Fig. 2. Fordelingen af antal katte på de forskellige typer ejendomme. PEn: parcelhus - enkeltliggende, PKl: parcelhus - klynge, PBy: parcelhus - by, FrA: fritidsagerbrug, FrH: fritidshusdyrbrug, FuA: fuldtidsagerbrug, FuH: fuldtidshusdyrbrug. Inkl. "ekstra landbrug". Fordelingen af ejendomme med hhv. 1-4 og 5 katte eller flere på de 7 ejendomstyper er signifikant forskellig ($p < 0{,}0001$; Pkl og Pby slået sammen).

= 32) (p = 0,6297). På arealmæssigt store fritidsagerbrug er der i gennemsnit 1,9 ± 2,0 katte (n = 45), hvilket ikke er signifikant forskelligt fra de 1,2 ± 1,7 katte der er gennemsnittet på små fritidsagerbrug (n = 16) (p = 0,0700). Tilsvarende er der på store fuldtidshusdyrbrug (≥ 10 ha, inkl. "ekstra landbrug") i gennemsnit 4,7 ± 4,9 katte (n = 26) og på små fuldtidshusdyrbrug (< 10 ha) 6,1 ± 7,2 katte (n = 12), hvilket ikke er signifikant forskelligt (p = 0,9374). På store fuldtidsagerbrug er der i gennemsnit 3,7 ± 4,0 katte (n = 7), mens der på små fuldtidsagerbrug er 2,5 ± 2,7 katte pr. ejendom (n = 33), hvilket heller ikke er signifikant forskelligt (p = 0,4978).

Blandt samtlige ejendomme med minimum én kat er det gennemsnitlige antal katte på ejendomme, hvor antallet af katte reguleres, og på ejendomme, hvor antallet af katte ikke

reguleres, hhv. 3,1 ± 2,6 (n = 213) og 2,2 ± 2,7 (n = 56), hvilket er signifikant forskelligt (p = 0,0002). På ejendomme, hvor én eller flere katte er neutraliseret, er der i gennemsnit 2,6 ± 2,2 katte (n = 121), hvilket ikke adskiller sig signifikant fra de 3,2 ± 2,9 katte, som er gennemsnittet på ejendomme, hvor ingen af kattene er neutraliserede (n = 148) (p = 0,1472).

Det gennemsnitlige antal katte på ejendomme, hvor kattene holdes som hhv. familiedyr og predator, er hhv. 2,1 ± 1,6 (n = 84) og 3,5 ± 2,9 (n = 90), hvilket er signifikant forskelligt (p = 0,0001).

På ejendomme hvor beboeren mener der er for mange katte i området, uanset om der er katte på ejendommen eller ej, er der i gennemsnit 1,2 ± 1,8 katte (n = 51). På ejendomme hvor beboer mener der ikke er for mange katte i området, er der i gennemsnit 1,7 ± 2,3 katte (n = 385), hvilket ikke er signifikant forskelligt (p = 0,1054).

Antallet af katte på landet i Danmark – et estimat (inkl. "ekstra landbrug")

Danmarks Statistiks definition af et landområde er, at der ikke er sammenhængende bebyggelser med mere end 200 indbyggere (Anonym 1997b). Da der i nærværende undersøgelses forsøgsområde ikke findes byer med mere end 200 indbyggere, lever området altså op til ovennævnte definition af et landområde.

Der er 288.466 husstande i landområder i Danmark (Anonym 1998). Hvis det forudsættes at der er samme fordeling af ejendomstyper i resten af Danmarks landområder som i denne undersøgelses forsøgsområde (hhv.: parcelhus: 258/451 * 100 = 57,3 %; fritidslandbrug: 131/451 * 100 = 29,0 %; fuldtidslandbrug : 62/451 * 100 = 13,7 %) da er der i alt i landområder i Danmark 165.291 parcelhuse, 83.655 fritidslandbrug og 39.520 fuldtidslandbrug. Ifølge Danmarks Statistik (Anonym 1997a) er driftsformen på fuldtidslandbrugene i hele Danmark fordelt på 51,3% agerbrug og 48,7% husdyrbrug, hvorfor fuldtidslandbrug kan deles op i 20.274 fuldtidsagerbrug og 19.246 fuldtidshusdyrbrug. Driftsformen på fritidslandbrug er ikke udspecificeret i statistikken.

Antallet af katte i landområder med fast tilknytning til en husstand kan således estimeres til 513.540 katte. (Se tab. 2 for udregning).

Tabel 2. Estimat af antal katte i Danmarks landområder.

Ejendomstype	Antal husstande i DK´s landområder [a]	Antal katte pr. ejendom i gennemsnit [b]	Antal katte i DK´s landområder	
Fuldtids~~agerbrug~~ [illegible]	20.274	~~5,03~~ 2,75	~~101.978~~	55.754
Fuldtids~~husdyr~~brug [illegible]	19.246	~~2,75~~ 5,03	~~52.927~~	96.807
Fritidslandbrug	83.655	2,41	201.609	
Parcelhus	165.291	0,95	157.026	
			~~513.540~~	511.196

[a] beregnet på baggrund af oplysninger fra Danmarks Statistik samt fordelingen af ejendomstyper i nærværende undersøgelse.

[b] resultatet af nærværende undersøgelse.

Beboernes holdning til antallet af katte i forsøgsområdet

På 86 % af de besøgte husstande mente beboerne ikke der var for mange katte i området, mens beboerne på 11 % af ejendommene mente der var for mange katte (fig. 3). Bevæggrundene hertil er opgjort i fig. 4. De mest almindelige årsager, i alt 49 %, er at kattene tager småfugle og/eller jagtbart vildt. Desuden mener 12 % at kattene sviner og yderligere 12 % at de er for dyre i foder.

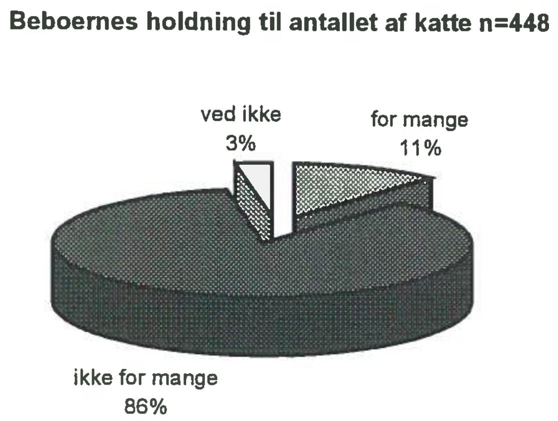

Fig. 3. Andelen af ejendomme hvor der, ifølge beboeren, er hhv. for mange katte og ikke for mange katte.

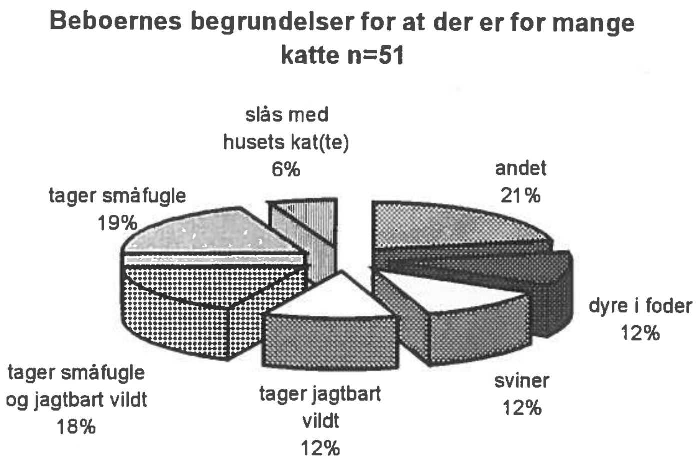

Fig. 4. Beboernes begrundelser for, at de mener, der generelt er for mange katte i området. "Andet" dækker bl.a. følgende grunde: indavl, syge katte, herreløse katte, tilløbne katte, trafikdrab samt bærer af lopper og sygdomme.

Formålet med katteholdet

Formålet med katteholdet for hhv. huskatte og staldkatte på samtlige ejendomme med kat(te) ses i fig. 5. Den altovervejende grund til at have kat er som familiedyr og predator, mens årsagerne "de er her bare/har altid været her" er relativt sjældne. Der er signifikant flere huskatte end staldkatte, der primært holdes som familiedyr, hhv. 45 % og 7 %, og omvendt signifikant flere staldkatte end huskatte, hhv. 56 % og 17 %, der primært holdes med det formål at holde skadedyr nede ($p < 0{,}0001$). Stort set samme andel af hus- og staldkatte holdes som både predator og familiedyr, hhv. 28 % og 24 %.

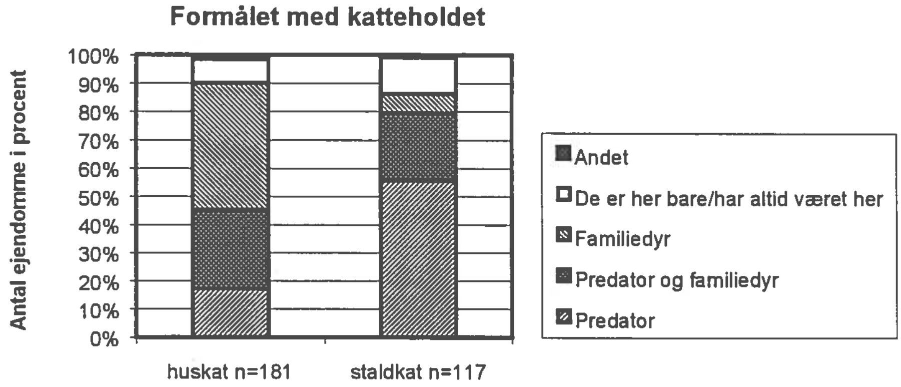

Fig. 5. Grunde til at holde kat, fordelt på huskat og staldkat. Der er statistisk signifikant forskel på fordelingen af de 5 grunde ($p < 0{,}0001$).

Kattenes pasning og sundhed

Bestandsbegrænsning

Eftersom der ikke er signifikant forskel på parcelhuse, fritids- og fuldtidslandbrug, mht. hvor mange der regulerer deres huskatte (p = 0,1821), sammenlægges de tre grupper. Heller ikke for staldkattenes vedkommende er der signifikant forskel på de tre ejendomstyper, mht. hvor mange der regulerer kattebestanden (p = 0,2975), hvorfor de tre ejendomstyper også lægges sammen her. For såvel hus- som staldkatte ses det, at der ikke er signifikant forskel på hvor mange af de adspurgte, der regulerer bestandene af hhv. hus- og staldkatte (p = 0,3175) (fig. 6). Disse to grupper af katte reguleres begge på over 70 % af alle ejendomme.

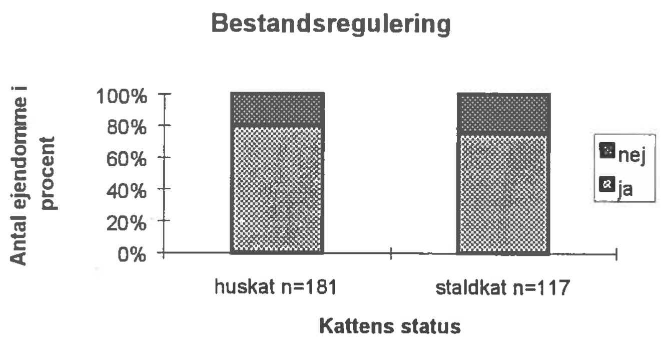

Fig. 6. Tilbøjeligheden til at regulere hhv. hus- og staldkatte. Der er ikke statistisk signifikant forskel på andelen, der regulerer huskatte, i forhold til andelen, der regulerer staldkatte (p = 0,3175).

Heller ikke når man opdeler i katte holdt som familiedyr og katte holdt som predator, er der signifikant forskel på hvor mange, af de adspurgte, der regulerer bestandene (p = 0,4226) (fig. 7). Også her er det mere end 70 % af adspurgte der regulerer bestanden.

Tilbøjeligheden til at begrænse kattebestanden blandt beboere, som mener, der er hhv. for mange katte og ikke for mange katte i området ses i fig. 8. Huskatte og staldkatte er

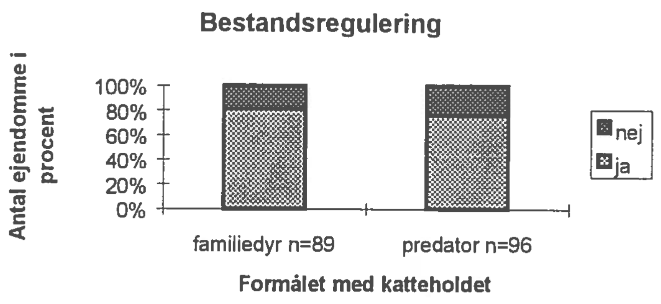

Fig. 7. Tilbøjeligheden til at regulere katte holdt som hhv. familiedyr og som predator. Der er ikke signifikant forskel på andelen af ejendomme, der regulerer familiekatte, i forhold til andelen, der regulerer predatorkatte (p = 0,4226).

slået sammen, dels fordi der er for få data (i det tilfælde hvor beboeren mener der er for mange katte i området), dels fordi der ikke er signifikant forskel på andelen af ejendomme, der regulerer bestandene af de to typer katte (i det tilfælde hvor beboeren mener der ikke er for mange katte [p = 0,1771]). Der er ikke signifikant forskel på fordelingen af tilbøjeligheden til at bestandsregulere blandt beboeren, der mener, der er hhv. for mange og ikke for mange katte i området (p = 0,2581).

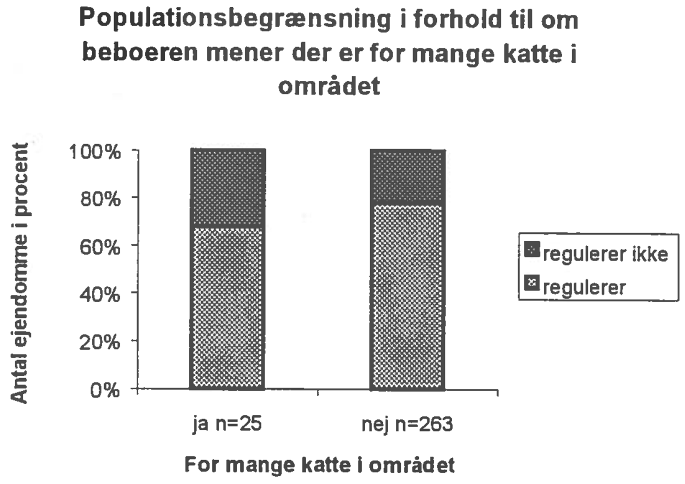

Fig. 8. Tilbøjeligheden til at foretage populationsbegrænsning blandt beboere, der mener, der er hhv. for mange og ikke for mange katte i området. Der er ikke signifikant forskel på andelen af ejendomme, der regulerer katte, på ejendomme hvor beboeren mener der er for mange katte i området, i forhold til andelen på ejendomme, hvor beboeren mener der ikke er for mange katte i området (p = 0,2581).

Der er signifikant forskel på hvor tilbøjelig ejendommenes beboere er til at kastrere én eller flere huskatte i forhold til staldkattene (fig. 9)(p < 0,0001). På 61 % af alle ejendommene med huskatte-hanner er én eller flere af disse kastreret, mens det tilsvarende tal er langt mindre, nemlig 13 %, for staldkattenes vedkommende.

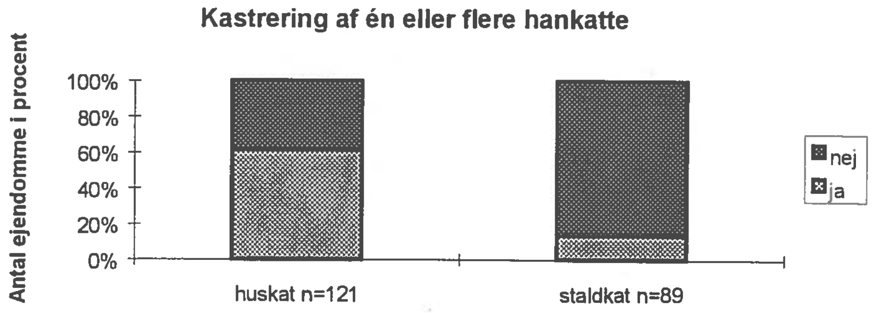

Fig. 9. Antal ejendomme med hhv. ingen og én eller flere kastrerede katte fordelt på hus- og staldkatte. Der er signifikant forskel på andelen af ejendomme, hvor én eller flere huskatte er kastreret, i forhold til andelen af ejendomme, hvor én eller flere staldkatte er kastreret (p < 0,0001).

Af fig. 10 ses, at hhv. 38 % og 31 % af adspurgte, som har hunkat(te) og som foretager bestandsregulering, benytter sig af p-piller. Enten alene eller i kombination med andre bestandsregulerende metoder til én eller flere af hhv. huskattene og staldkattene. Der er ikke signifikant forskel på brugen af p-piller for de to grupper katte (p = 0,3783).

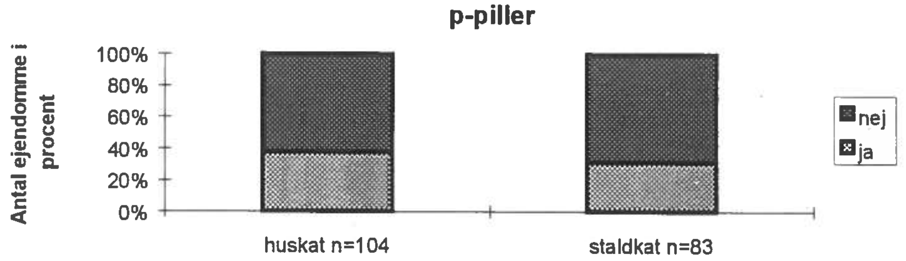

Fig. 10. Brugen af p-piller som populationsbegrænsende middel, til én eller flere af hunkattene, fordelt på hus- og staldkatte. Der er ikke signifikant forskel på andelen af ejendomme, der anvender p-piller til huskatte, i forhold til andelen, der anvender p-piller til staldkatte (p = 0,3783).

Der er derimod signifikant forskel på, hvor mange der steriliserer hhv. deres hus- og staldkatte (p = 0,0003) (fig. 11). 44 % af de adspurgte har steriliseret én eller flere af deres huskatte, mens det kun er 19 % der har steriliseret én eller flere af deres staldkatte.

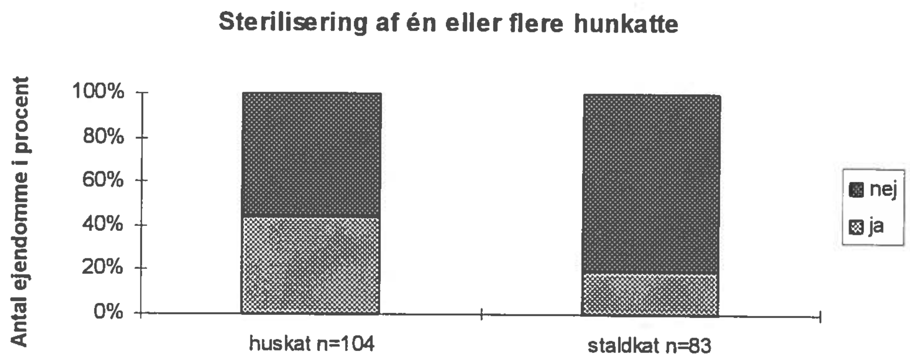

Fig. 11. Sterilisering af én eller flere hunkatte fordelt på hus- og staldkatte. Der er signifikant forskel på andelen af ejendomme, hvor én eller flere huskatte er steriliseret, i forhold til andelen af ejendomme, hvor én eller flere staldkatte er steriliseret (p = 0,0003).

Af de adspurgte som har hunkat(te), og som foretager bestandsregulering, er det for huskattenes vedkommende 21 %, som benytter sig af aflivning af kattekillinger. Enten alene eller kombineret med andre bestandsregulerende metoder til én eller flere af katte-ne. Det tilsvarende tal er 51 % for staldkattenes vedkommende (fig. 12). Sagt med andre ord, så aflives der ikke killinger af huskatte på 79 % af husstandene og der aflives ikke killinger af staldkatte på 49 % af husstandene. Der kan dog finde anden populationsbe-grænsning sted. Andelen af husstande der afliver killinger og husstande der ikke afliver killinger, for de to kategorier af katte, er signifikant forskellig (p < 0,0001).

Aflivning af killinger

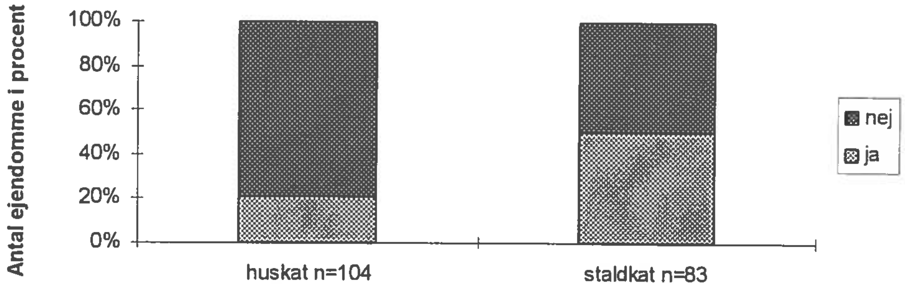

Fig. 12. Aflivning af killinger som populationsbegrænsende middel fordelt på hus- og staldkatte. Der er signifikant forskel på andelen af ejendomme, der afliver huskattekillinger, i forhold til andelen, der afliver staldkattekillinger ($p < 0{,}0001$).

Kun 5 % af adspurgte, svarende til 5 ejendomme, som har hunkat(te) og som foretager bestandsregulering af huskatte, benytter sig af aflivning af voksne katte alene eller kombineret med andre bestandsregulerende metoder til én eller flere af huskattene (fig. 13). Dette er signifikant færre end for staldkattenes vedkommende ($p = 0{,}0124$), idet 16 % af adspurgte, svarende til 13 ejendomme, afliver voksne staldkatte som populationsbegrænsning (fig. 13). På én ejendom var der både hus- og staldkatte, dvs. på i alt 17 ejendomme aflives der hus- og/eller staldkatte når hus- og staldkatte lægges sammen. På 11 ejendomme skydes kattene, på 4 ejendomme bruges dyrlægen, mens drukning og gasning benyttes på hver 1 ejendom (begge ejendomme med staldkatte).

Aflivning voksne katte

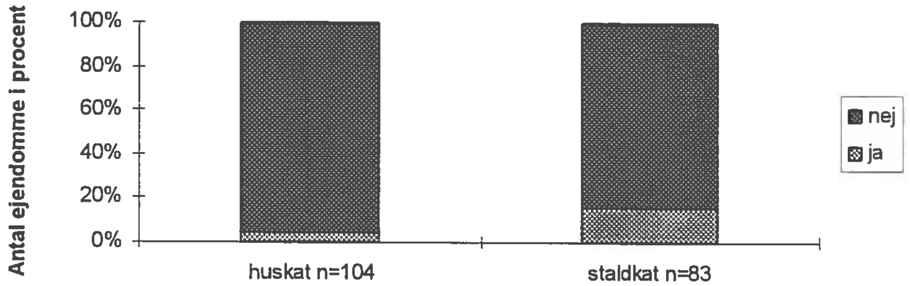

Fig. 13. Aflivning af voksne katte som populationsbegrænsende middel fordelt på hus- og staldkatte. Der er signifikant forskel på andelen af ejendomme, der afliver voksne huskatte, i forhold til andelen, der afliver voksne staldkatte ($p = 0{,}0124$).

Metoden til aflivning af hhv. huskatte- og staldkattekillinger, uanset køn, blandt de adspurgte, der benytter sig af aflivning af killinger som bestandsregulerende metode, ses i fig. 14. Der er ikke tilstrækkeligt med data til at lave statistik på de to grupper. Den mest udbredte metode, for både huskatte og staldkatte, er at slå killingens hoved mod et hårdt underlag. Ingen af de adspurgte gassede killinger. Såvel hus- som staldkattekillinger, blev på 10 % af ejendommene, aflivet ved drukning. På 30 % af ejendommene blev dyrlægen brugt til aflivning af staldkattekillinger, hvorimod det kun var tilfældet for huskattekillingernes vedkommende på 5 % af ejendommene. Det er signifikant mere udbredt at aflive samtlige killinger af huskatte end af staldkatte, hhv. 60 % og 27 %, (fig. 15) (p = 0,0323). Staldkattene får, oftere end huskattene, lov at beholde en enkelt killing, hhv. 64 % og 35 %.

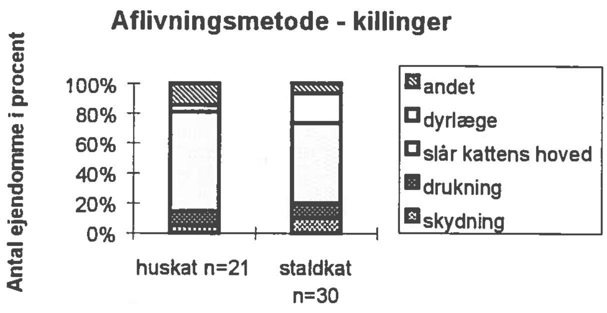

Fig. 14. Metoder til at aflive killinger fordelt på huskat og staldkat. Der er for få data til at udføre statistik. "Andet" dækker for huskat: 1 ved ikke, 1 æter, og 1 knækker nakken; for staldkat: 1 ved ikke og 1 hunden.

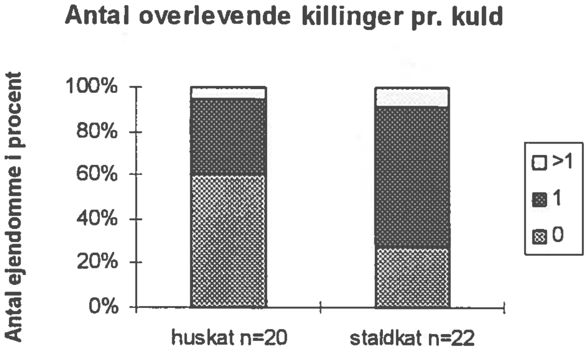

Fig. 15. Killinger pr. kuld, der overlever populationsreguleringen på husstanden, opdelt i hhv. hus- og staldkatte. Der er signifikant forskel på andelen af ejendomme, der fjerner alle huskattens killinger, i forhold til andelen, der fjerner alle staldkattens killinger (p = 0,0323).

Af de 435, der blev spurgt om de kender nogle, der drukner deres voksne katte og/eller killinger p.t., har de 5 % svaret bekræftende, 92 % benægtende og 3 % ved ikke.

I fig. 16 ses grunden til valg af aflivningsmetode for de to typer katte slået sammen. Af de 52, der afliver katte eller killinger er den mest almindelige årsag, 40 %, at de kan udføre det selv, mens 19 % ikke kan aflive kattene selv. 21 % gør som de gør fordi de altid har gjort sådan, 12 % fordi de mener, det er det mest humane. Blandt de beboere, der har valgt aflivningsmetode ud fra hvad de anser som det mest humane, aflives kattene på 3 ejendomme ved at slå killingernes hoved mod en hård genstand, på 2 ejendomme ved hjælp af dyrlægen, på 1 ejendom ved skydning og på 1 ejendom ved drukning!. Blandt de beboere, der ikke slev kan aflive kattene, bruges hovedsagelig dyrlægen, men på hver 1 ejendom benyttes hhv. beboerens svigerfar og nabo.

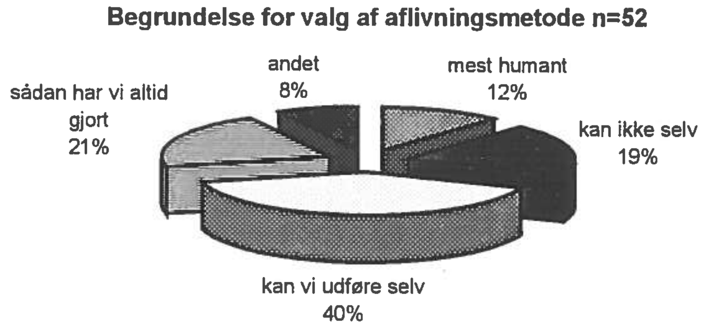

Fig. 16. Beboerens begrundelse for valg af aflivningsmetode.

De adspurgtes mening, om hvorvidt deres forsøg på at bestandsregulere lykkes, ses i fig. 17 udspecificeret i hus- og staldkatte. Kun 2 % af adspurgte mener at reguleringen af huskatte er mislykkedes, mens der er signifikant flere, nemlig 15 %, der mener at reguleringen af staldkatte er mislykkedes (p = 0,0044). Tilsvarende er det hhv. 98 % og 85 % der mener at populationsbegrænsning af hhv. huskatte og staldkatte er lykkedes.

Populationsbegrænsningens succes

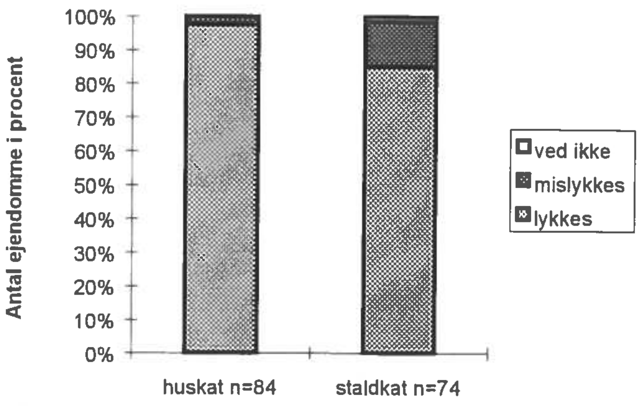

Fig. 17. Populationsbegrænsningens succes for hhv. huskatte og staldkatte. Der er signifikant forskel på andelen af ejendomme, hvor populationsbegrænsningen af huskatte er mislykkedes, i forhold til andelen, hvor begrænsningen af staldkatte er mislykkedes (p = 0,0044).

Fodring og foderstand

På spørgsmålet hvor mange katte der fodres, hhv. "ingen", "nogle" og "alle", på de respektive ejendomme, svares der på 264 ejendomme ud af 269 at alle katte fodres. Dvs. 98,1 % af alle de adspurgte, der har kat(te), fodrer alle deres katte. På én husstand fodres nogle af kattene, svarende til 0,4 %; mens kattene på 4 ejendomme ikke fodres, svarende til 1,4 % af ejendommene. Blandt de ejendomme, hvor alle ejendommens katte fodres, er der for de 173 ejendomme påtegnet, om det kun er de katte der hører til på ejendommen der fodres, eller om også fremmede katte fodres. Heraf fodres på 58 % af ejendommene udelukkende de katte der hører til. På de resterende 42 % af ejendommene fodres desuden fremmede katte.

Blandt de beboere, der ikke holder kat, er der 16 ud af i alt 104 adspurgte, svarende til 15 %, der fodrer nogle, eller alle de katte, der viser sig på ejendommen (fig. 18).

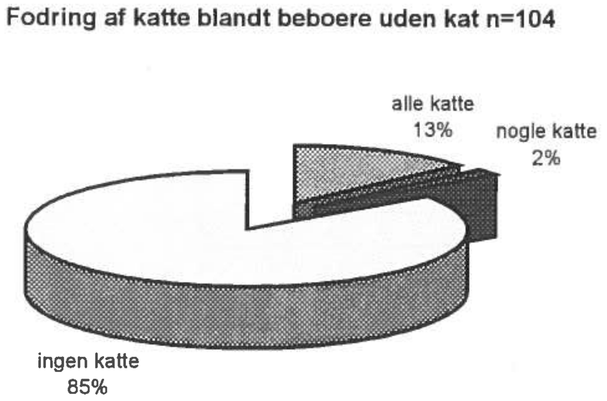

Fig. 18. Fodring af katte på ejendomme, hvor der ikke holdes kat.

Beboerne på ejendommen blev spurgt om deres mening om kattenes aktuelle foderstand. På 97 % og 98 % af samtlige ejendomme mente beboeren, at foderstanden hos samtlige hhv. hus- og staldkatte var god. Der er altså ganske få katte, som efter beboernes mening, var i dårlig foderstand (tab. 3). Intervieweren fandt ingen uoverensstemmelser med beboernes observationer.

Tabel 3. Beboernes vurdering af foderstanden hos ejendommens katte.

Foderstand	Huskat	Staldkat	I alt
God alle	175	113	288
God halvdelen eller de fleste	2	0	2
Dårlig de fleste	0	1	1
Dårlig alle	0	0	0
Ved ikke	3	1	4
I alt	180	115	295 [a]

[a] én kat færre end i tab. 4 pga. 1 ubesvaret.

Sygdom

Beboerne på ejendommen blev ligeledes spurgt, om deres mening, om den aktuelle sundhedstilstand hos såvel huskatte som staldkatte. Sundhedstilstanden hos huskatte og staldkatte blev, på hhv. 96 % og 95 % af samtlige besøgte ejendomme, angivet til at være god. Der er altså, ifølge beboerne, ganske få katte som ikke er raske (tab. 4). Heller ikke vedrørende kattenes sundhedstilstand observerede intervieweren uoverensstemmelser med beboernes angivelser.

Tabel 4. Beboernes vurdering af sundhedstilstanden hos ejendommens katte.

Sundhedstilstand	Huskat	Staldkat	I alt
God alle	173	110	283
God halvdelen eller de fleste	3	4	7
Dårlig de fleste	0	1	1
Dårlig alle	3	1	4
Ved ikke	1	0	1
I alt	180	116	296

På 88 % af samtlige ejendomme med huskatte og 76 % af samtlige ejendomme med staldkatte har beboeren ikke observeret sygdom blandt hhv. hus- og staldkattene (fig. 19). Hyppigheden af observerede tilfælde af influenza/kattesyge er signifikant hyppigere blandt staldkatte end blandt huskatte, hhv. 21 % og 8 % (p = 0,0089).

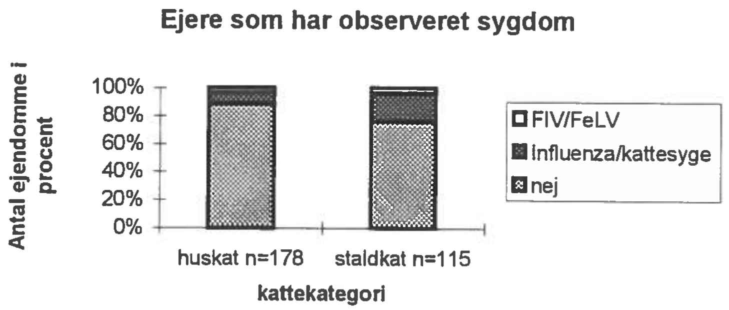

Fig. 19. Ejendomme hvorpå der er observeret sygdom blandt hhv. huskatte og staldkatte. Der er signifikant forskel på andelen af ejendomme, hvor der er observeret sygdom blandt huskattene, i forhold til andelen, hvor der er observeret sygdom blandt staldkattene (p = 0,0089).

Tilsvarende vaccineres huskattene signifikant oftere end det er tilfældet for staldkattene (p < 0,0001; den ene ejendom hvor kun nogle af staldkattene vaccineres sammenlagt med de ejendomme hvor alle staldkattene vaccineres, og de to ejendomme hvor beboeren ikke ved om kattene vaccineres sammenlagt med de ejendomme hvor kattene ikke vaccineres). På i alt 27 % af samtlige ejendomme med huskatte vaccineres alle huskatte, mens alle staldkatte vaccineres på i alt 6 % af samtlige ejendomme med staldkat (fig. 20).

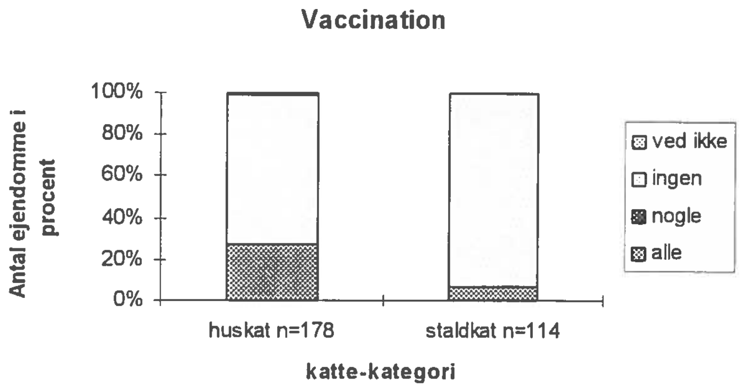

Fig. 20. Ejendomme hvorpå hhv. huskattene og staldkattene vaccineres. Der er signifikant forskel på andelen af ejendomme, hvor huskattene er vaccinerede, i forhold til andelen, hvor staldkatte er vaccineret ($p < 0{,}0001$; når "nogle" sammenlægges med "alle", og "ved ikke" sammenlægges med "ingen").

Ved sygdom blandt kattene er der tendens til at beboerne handler forskelligt overfor hus- og staldkatte (fig. 21), idet 75 % af huskattene mod 50 % af staldkattene hjælpes, dvs. aflives eller behandles ($p = 0{,}0808$).

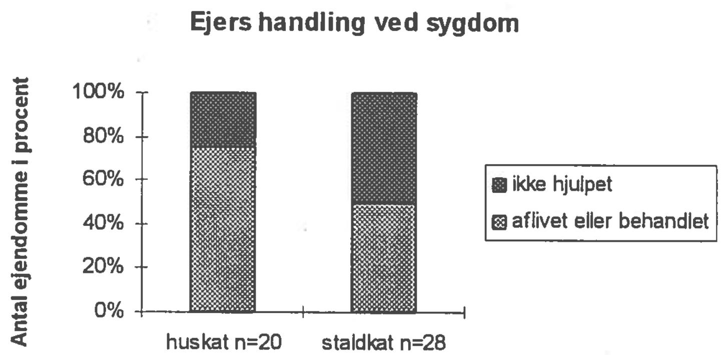

Fig. 21. Ejers handling ved sygdom, fordelt på huskatte og staldkatte. Der er tendens til, at der er forskel på andelen af ejendomme, der hjælper huskatte, i forhold til andelen, der hjælper staldkatte ($p = 0{,}0808$).

Kattenes reproduktion og bestandstilvækst

Kattenes reproduktion

Af fig. 22 ses, at killingerne hovedsagelig fødes forår (53 %) og sommer (35 %), mens kun en mindre del fødes i efteråret (11 %) og om vinteren (1 %). Ifølge 45 % af de adspurgte er der stor variation mellem årene mht. hvornår på året kattene får killinger. Lige så mange, 45 %, mener dog, at der ikke er nogen variation mellem årene mht. hvornår på

året killingerne fødes, og 10 % ved ikke. Størstedelen af de adspurgte, 73 %, mener desuden, at kuld født i foråret ikke er større end kuld født sommer eller efterår, 9 % mener forårskuldene er større end sommer- og efterårskuldene og 18 % ved ikke om der er forskel.

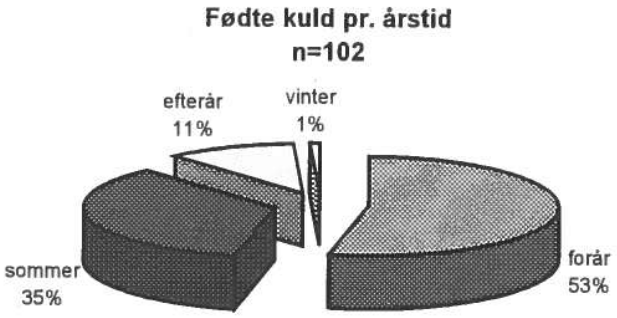

Fig. 22. Fødselstidspunkt fordelt på årstid.

I nærværende undersøgelse blev beboerne spurgt om størrelsen på de fødte killingekuld i det forløbne år. Beboerne havde samlet kendskab til i alt 102 kuld med i alt 380 killinger. Hvilket svarer til i gennemsnit 3,73 ± 1,41 (SD) killinger pr. kuld. Beregnes kuldstørrelsen, på baggrund af det antal killinger beboerne mener der er gennemsnitligt blandt deres katte, får kattene 3,84 ± 0,99 (SD) killinger pr. kuld.

Bud på bestandstilvæksten i området

Ifølge beboerne på de 451 ejendomme, kom der altså sidste år mindst 380 nye killinger. For de 226 killingers vedkommende har beboeren angivet, at kattebestanden på ejendommen reguleres vha. aflivning af killinger eller voksne. Derfor risikerer disse 226 killinger at blive slået ihjel. Aflivningen af killingerne er dog ikke helt effektiv. Dels fordi killinger af hunkatte, der ikke er tamme, ikke altid kan findes mens de er små, og dels fordi beboerne, på mere end 35 % af samtlige ejendomme (fig. 15), har svaret at de lader hunkatten beholde én til flere killinger pr. kuld. I denne undersøgelse er der fundet at kuldstørrelsen er 3,73 killinger, hvilket betyder at de 226 killinger, der risikerer at blive dræbt af ejeren, svarer til 60,6 kuld. Når der for 35 % af kuldenes vedkommende beholdes mindst én killing pr. kuld, er der altså yderligere 21 killinger der overlever (60,6 kuld * 35 %). Dermed er der 205 der dræbes af ejeren (226 – 21). Dvs. der er en restproduktion på 175 killinger pr. år (380 – 205).

DISKUSSION

Besøgsinterviewmetodens umiddelbare fordel ligger i den direkte konfrontation mellem interviewer og svarperson, med deraf følgende høj kvalitet og troværdighed af oplysningerne (Christoffersen 1984). Desuden er det en styrke for undersøgelsen, at samtlige interviews er udført af én og samme person. Derved er der stor ensartethed i dataindsamlingen. Ulempen ved besøgsinterviews er risikoen for at svarpersonerne påvirkes af interviewerens optræden eller den institution intervieweren repræsenterer (Jensen & Nielsen 1985). Af samme grund repræsenterede intervieweren en neutral forskningsinstitution, Zoologisk Museum, under interviewet, ikke interesseorganisationen, Dyrenes Beskyttelse. Besøgsinterviews har desuden den ulempe, at de er meget tidskrævende. Nærværende undersøgelse dækker derfor også kun et relativt lille landområde med deraf følgende repræsentative problemer, idet det er umådelig svært af finde et så lille område der fuldstændig repræsenterer alle Danmarks landområder. Tilgengæld er der opnået en meget høj svarprocent (92,4 %), hvilket er en styrke for undersøgelsen. Dermed er der nemlig stor sikkerhed for at undersøgelsens resultater er dækkende for det pågældende område.

Den brugte dataindsamlingsmetode har endvidere den ulempe, at kun katte med et fast tilhørsforhold til en husstand registreres, mens metoden udelukker katte som ikke har et fast tilhørsforhold. Vi har således stadig ikke nogen ide om, hvor mange katte der er helt vildtlevende, eller som strejfer så meget at beboerne i området ikke genkender dem. Alle resultater og diskussioner i nærværende undersøgelse omhandler derfor katte med et fast tilhørsforhold til en husstand, hvilket langtfra er optimalt. På trods heraf giver metoden mulighed for indsamling af værdifuld viden om katte på landet. Viden som ikke tidligere er forsøgt indsamlet i Danmark.

Antallet af katte

I nærværende undersøgelse har mere end hver anden ejendom én eller flere katte med fast tilknytning til en husstand og bestandstætheden i området er 16,6 – 20,0 katte pr. km^2. Men den beregnede bestandstæthed i området er et absolut minimum, som kan være større. Dels fordi den brugte metode ikke giver mulighed for at indsamle data om vildtle-

vende katte, dels fordi beboerne i området muligvis undervurderer antallet af katte på deres ejendom. Sidstnævnte viste sig at være tilfældet blandt beboere i Wisconsin, USA, der underestimerede antallet af katte på deres ejendom med 11 % (Coleman & Temple 1993). Desuden kan der være katte med fast tilhørsforhold til flere husstande, f.eks. i kraft af fodring på disse ejendomme. Hvis disse katte ikke er knyttet til nogen af disse ejendomme i en sådan grad, at beboeren vurderer at kattene hører til på ejendommen, vil kattene ikke være repræsenteret i estimatet af antal katte. På den anden side, er der også en risiko for, at disse katte tæller dobbelt, hvis mere end én ejendom betragter katten som tilhørende.

Hvad antal katte pr. ejendom angår udgør fuldtidshusdyrbrug i nærværende undersøgelse den ene ekstrem, med flest katte/ejendom, og parcelhuse i bymæssig bebyggelse den anden ekstrem, med færrest katte/ejendom. Dette tyder på at antallet af katte på en ejendom afhænger af, hvor isoleret ejendommen er placeret og af hvor meget dyrehold der findes på ejendommen. Landbrugets areal, derimod, har tilsyneladende ikke nogen effekt på antallet af katte på ejendommen, uanset driftsform og fuldtids-/fritidsbasis.

I tabel 6 ses en summering af relevante undersøgelser af populationstætheder af katte på landet. Som det ses, er der stor variation i bestandstætheden af katte i områder på landet i de forskellige lande. Bestandstætheden i nærværende undersøgelse er højest, hvilket kan skyldes, at der er flere husstande på et givet areal end i de andre undersøgelsesområder. Tilgengæld er der, i nærværende undersøgelse, i gennemsnit færre katte pr. ejendom med kat(te) end de to undersøgelser fra USA (Coleman & Temple 1993; Warner 1985). Dette kan skyldes, at der procentvis er færre landbrug og flere parcelhuse i forsøgsområdet. Parcelhuse har, ifølge nærværende undersøgelse, i gennemsnit færre katte pr. ejendom end fritids- og fuldtidslandbrug uanset driftsform. Såfremt antallet af katte pr. ejendom med katte i et område afhænger af fordelingen af landbrug og ikke-landbrug, må man dog forvente, at der var mindre end i gennemsnit 3 katte pr. ejendom med katte i den svenske undersøgelse, hvor der ikke var nogen fungerende landbrug (Liberg 1980). At det ikke er tilfældet skyldes måske, at der i nærværende undersøgelse, i modsætning til den svenske, er inddraget parcelhuse i bymæssig bebyggelse. Parcelhuse i bymæssig be-

byggelse ligger i bunden med 0,54 katte pr. ejendom, hvilket er signifikant mindre end såvel enkeltliggende parcelhuse som fritidslandbrug og fuldtidslandbrug uanset driftsform.

Lokalitet	Populationstætheden af katte / km^2	Gennemsnitlig antal katte / ejendom med kat(te)	Antal husstande / 50 km^2	Landbrug : ikke landbrug	Kilde
Sverige, militært område, ukultiveret men 40 % afgræsses	2,5 – 3,3 [1]	3 [2]	64	0 : 100	[1] Liberg 1980, [2] Liberg 1984
Lowland England, landbrugsområde *	4,3 – 6,3	4,0		100 : 0 **	Macdonald et al., 1987
USA, Illinois, landområde	6,3 – 8,7	5,6 ± 0,9 (SD)	385	75 : 25	Warner, 1985
USA, Wisconsin, landbrugsland	10 – 14	7,6		82 : 18	Coleman & Temple, 1993
Danmark, landbrugsområde	16,6 – 20,0	2,9 ± 2,6 (SD)	519	43 : 57	Denne undersøgelse

Tabel 6. En oversigt over relevante undersøgelser af populationstætheder af katte i landområder. * Kun landbrug ekskl. andre husstande. ** Fordelingen af landbrug : ikke landbrug i datamaterialet er ikke repræsentativt for fordelingen af samme i forsøgsområdet, fordi alle ikke landbrug er udelukket.

Estimatet af antal katte på landet i Danmark, med fast tilknytning til en husstand, er behæftet med visse svagheder. Dels er det, som før nævnt, svært at finde et området i Danmark, der nøjagtigt repræsenterer de samlede landområder i Danmark. Derfor havde det været en styrke for undersøgelsen, hvis undersøgelsen var blevet gentaget i andre egne af landet. Af økonomiske og tidsmæssige årsager var dette imidlertid ikke muligt.

De 513.540 katte, der er resultatet af beregningerne i nærværende undersøgelse, er et absolut minimumstal, idet det er baseret på de katte der med sikkerhed var på de ud-

spurgte ejendomme på besøgstidspunktet. Dertil skal lægges de katte som folk ikke ved de har og de katte som er helt vildtlevende. Bestandstætheden af de vildtlevende katte er sandsynligvis ubetydelig. I Norditalien blev bestandstætheden af de vildtlevende katte estimeret til 1,5 katte / km^2 (Genovesi et al. 1995), mens den er nede på 0,3 katte / km^2 i det sydlige Sverige (Liberg 1980). Lignende undersøgelser af vildtlevende kattes bestandstætheder i Danmark er ikke foretaget. Vildtbiolog Tommy Asferg, Danmarks Miljø Undersøgelser, Kalø (pers. comm.), mener at de vildtlevende katte, i et bestandsmæssigt perspektiv, er uden betydning. Der findes enkelte observationer af katte der reproducerer vildtlevende i Danmark. Dvs. hvor reden er placeret udenfor menneskelig bebyggelse og hvor der ikke er tale om, at katten har flyttet sine killinger ud fra menneskelig bebyggelse som følge af forstyrrelser. Disse observationer er imidlertid så fåtallige og behæftet med så stor usikkerhed, at det ikke giver grund til at tro, at der er en stor population af vildtlevende katte på landet i Danmark, om end der sikkert findes nogle (Baagøe, H. B., pers. comm.).

På trods af, at estimatet af antal katte på landet i Danmark med fast tilknytning til en husstand skal tages med forbehold, giver estimatet dog et indtryk af kattebestandens størrelse og hermed også af nødvendigheden af, at inddrage kattene i den biologiske og dyreværnsmæssige debat. Ikke mindst sat i perspektiv med, at den samlede bestand af ræve i Danmark næppe overstiger 250.000 (Asferg, T, pers. comm.).

Kattenes reproduktion og bestandstilvækst

I nærværende undersøgelse får kattene, hus- og staldkatte samlet, hovedsagelig killinger forår og sommer. Ifølge beboerne er forårskuldene tilsyneladende ikke større end sommer- og efterårskuldene. Den gennemsnitlige kuldstørrelse i nærværende undersøgelse er 3,73 killinger pr. kuld. Det er lidt færre end de 4,3 killinger i gennemsnit pr. kuld, for såvel tamme som vilde katte fra Storkøbenhavnsområdet, som dyrlæge Tom S. Kristensen (pers. comm.) er kommet frem til. Undersøgelsen er hovedsagelig baseret på en optælling af antal fostre ældre end 14 dage. Forskellen er derfor muligvis udtryk for, dels at der sandsynligvis går fostre til under drægtigheden, dels at beboerne i nærværende undersøgelse ikke får kendskab til samtlige killinger, f.eks. fordi hunkatten gemmer dem. I

kraft af at beboerne ikke får kendskab til samtlige killinger, vil der sandsynligvis være en større tilgang af katte til området end de 380 killinger der, ifølge beboerne i nærværende undersøgelse, blev født i perioden forår ′97 – forår ′98. 175 killinger ud af de 380 overlevede sandsynligvis ejerens populationsbegrænsning. Disse 175 killinger har erstattet det naturlige frafald af katte i området, f.eks. pga. alder, sygdom og trafik. I det forgangne år var dette frafald af voksne katte fra området på 140 katte, ifølge beboernes oplysninger. Dødeligheden af juvenile og subadulte katte kendes ikke. Eftersom dødeligheden blandt sudadulte katte generelt er stor (Clausen 1996) må man dog formode, at bestanden af katte i området er nogenlunde stabil med det nuværende niveau af populationsbegrænsning og kattens popularitet som familiedyr. På grund af kattens specielle levevis og tilknytning til mennesket, afhænger bestandstætheden af katte imidlertid meget af menneskets adfærd og holdning til kattene. Hvis katte bliver stadig mere populære som familiedyr vil områdets kattebestand sandsynligvis stige i fremtiden, såfremt der opretholdes samme niveau af populationsbegrænsning som p.t. og der ikke sker større ændringer i fordelingen af parcelhuse:landbrug i området.

Kattene og faunaen

Det der i høj grad adskiller katte fra alle andre husdyr er, at de på en gang spiser af "menneskets hånd" og samtidig selvbetjener sig fra naturens spisekammer. Sidstnævnte har lagt dem for had blandt jægere og ornitologer. Der findes adskillige studier af kontinentale kattes predation på faunaen. I de fleste undersøgelser er der udelukkende fokuseret på en oversigt over menuen, mens der kun findes få undersøgelser af predationens påvirkning af diversiteten og mængden af de prederede arter. Relationerne mellem katte og faunaen er i sig selv et litteraturstudie værd. I det følgende skal disse relationer nævnes meget kort.

Kontinentale katte prederer hovedsagelig på små pattedyr, specielt gnavere, unge kaniner og harer, mens fugle kun udgør en lille del af deres føde og krybdyr endnu mindre (Fitzgerald 1988) Undersøgelser fra Sverige viser, at katten var på niveau med ræven, mht. predation på harer, og at den samlede predation langt oversteg antallet af afskudte og trafikdræbte harer. De to arter stod for i alt 90 % af den samlede predation på harer.

Kattens betydning for predationen af fasaner var derimod sekundær, idet ræven stod for over 2/3 heraf (Erlinge et al. 1984). I visse tilfælde spiser predatorerne en betydelig del af den årlige produktion af et byttedyr. Således blev knap 40 % af den årlige produktion af harer spist af predatorer i den svenske undersøgelse (Erlinge et al. 1984). Betydningen af ræve og kattes predation på populationerne af harer og andre byttedyr er imidlertid usikker, idet mange faktorer spiller ind (Erlinge et al. 1984). Bl.a. mangler man viden om, og i hvor høj grad, byttedyrene kan kompensere for predationen gennem et større reproduktivt output, og hvor stor del af de prederede byttedyr der ville dø af andre årsager hvis de ikke var blevet ædt af katte. Kattenes predation på de vilde småfugle i Sverige vurderes af Svensson (1996) at være lille, hvis man ser på hele den svenske fauna. Derimod tyder undersøgelsen på at predationen omkring bebyggelse kan være så stor, at den påvirker fuglenes antal (Svensson 1996). Om kontinentale katte må altså siges, at de er en del af faunaen, på lige fod med andre predatorer, men at det ikke nødvendigvis er så fatalt for de enkelte arter af byttedyr. Endelig kan kattene konkurrere med f.eks. brud, hermelin og andre rovdyr om byttedyrene. Der er, så vidt vides, ikke lavet nogle undersøgelser af effekten, af denne konkurrence, på populationen af rovdyrene.

Kattene og mennesket

I nærværende undersøgelse mente 11 % af samtlige beboere i forsøgsområdet at der var for mange katte i området. Den altovervejende grund hertil var at kattene tager småfugle og jagtbart vildt. Blandt de beboere der havde kat(te) og som mente der var for mange katte i området, må genen dog have været begrænset. Deres holdning til antallet af katte havde nemlig ikke nogen effekt på antallet af katte på deres ejendom og på deres tilbøjelighed til at bestandsregulere. Dette er i modstrid med hvad Coleman & Temple (1993) fandt i Wisconsin. Her viste det sig, at de beboere, der mente der var for mange katte i området, var mere tilbøjelige til at foretage populationsregulering, end andre beboere. At der i nærværende undersøgelse ikke var færre katte og større brug af populationsbegrænsning, på de ejendomme hvor beboeren mente der var for mange katte i området, kan skyldes at beboerne mener, dels at det er de andre der har for mange katte, dels at et stort antal katte er nødvendigt for at holde skadedyrene nede.

I nærværende undersøgelse er der flere katte på ejendomme, hvor kattene holdes som predatorer, end på ejendomme hvor de holdes som familiedyr. Dette stemmer overens med Coleman & Temple (1993) som fandt, at husstande hvor kattens funktion primært var predator eller både predator og familiedyr, havde flere katte, end husstande hvor der blev holdt katte primært som familiedyr eller af andre grunde. Tilsvarende fandt Macdonald et al. (1987) at landmænd, der havde katte hovedsagelig som nyttedyr, havde flere katte end landmænd, der havde katte af andre årsager. Det er p.t. ikke muligt, på baggrund af videnskabelige undersøgelser, at sige hvor mange katte der skal til for at holde en ejendom ugeneret af mus og rotter. Det afhænger, ifølge Turner & Meister (1988), delvis af kattens køn, idet hunkatte med killinger er de mest effektive musejægere, forudsat at alle katte fodres ens, og at der hovedsagelig er gnavere at jage. Derimod mangler der bevis for, at katte der ikke fodres er mere ivrige jægere end katte der fodres (Turner & Meister 1988). Derfor skal man ikke afholde sig fra at fodre sine katte.

Ifølge Elton (1953) er 4 katte tilstrækkeligt til at hindre en invasion af rotter på en ejendom. Katte fanger hovedsagelig juvenile og subadulte rotter (Childs 1986) og enhver rotte der ankommer til en gård, beboet af katte, er umiddelbart meget udsat for at blive fanget, fordi den i modsætning til kattene er ukendt med området (Elton 1953). I nærværende undersøgelse har 14 (36 %) fuldtidshusdyrbrug, 7 (18 %) fuldtidsagerbrug, 16 (23 %) fritidshusdyrbrug, 6 (10 %) fritidsagerbrug og 10 (6 %) enkeltliggende parcelhuse 5 katte eller flere. Hvis man ganger det med det gennemsnitlige antal katte på de respektive ejendomstyper, er der hele 161 katte mere i området end det er nødvendigt for at holde rotter i skak. Dvs. 21 % af samtlige katte i området kunne sandsynligvis undværes, hvilket ville give en bestandstæthed på 13 katte / km^2. Hvorvidt 4 katte pr. ejendom er tilstrækkeligt til at regulere musebestandene er uvist, men viden herom er essentielt i forbindelse med den fremtidige forvaltning af kattebestanden.

Kattenes pasning og sundhed – dyreværnsmæssige aspekter

På samtlige ejendomstyper reguleres mere end 70 % af kattene, uanset om de opdeles i hus- og staldkatte eller familie- og predatorkatte. Ifølge beboerne lykkes populationsbegrænsningen af såvel hus- som staldkatte for størstedelens vedkommende. Selvom tilbø-

jeligheden til at lave populationsbegrænsning ikke medfører et færre antal katte pr. ejendom. Derimod er der i gennemsnit flere katte på ejendomme, hvor der laves populationsbegrænsning, end på ejendomme hvor der ikke laves populationsbegrænsning. Dette kan skyldes, at populationsbegrænsningen ikke er effektiv nok, eller at folk først iværksætter en populationsbegrænsning når det er pinedød nødvendigt. Det er også muligt, at effekten af populationsbegrænsningen ikke er synlig, fordi populationsbegrænsningen først for nylig er introduceret på ejendommen. Man burde imidlertid kunne se en effekt af populationsreguleringen allerede det efterfølgende år, uanset hvordan reguleringen finder sted. I nærværende undersøgelse har neutralisering af én eller flere af en ejendoms katte heller ikke nogen effekt på antallet af katte. Dette kan forklares ved, at det ikke, eller kun sjældent, er alle katte som er neutraliserede, hvilket ikke kan udelukkes, men desværre heller ikke hverken be- eller afkræftes i nærværende datamateriale. Coleman & Temple (1993) derimod fandt, at de adspurgte, der neutraliserede nogle eller alle deres katte, havde færre katte end dem, der ikke neutraliserede overhovedet. I nærværende undersøgelse er én eller flere hhv. han- og hunkatte neutraliserede på hhv. 61 % og 44 % af samtlige ejendomme med huskatte. Tilsvarende er alle eller nogle hhv. han- og hunkatte neutraliserede på hhv. 13 % og 19 % af samtlige ejendomme med staldkatte. Med hensyn til neutralisering behandles de to typer katte altså forskelligt, hvilket sandsynligvis skyldes en kombination af økonomi og menneskets forhold til de to typer katte. Ofte vil der være et tættere forhold mellem huskat og menneske end mellem staldkat og menneske. Dette bekræftes af nærværende undersøgelse, hvor huskatte signifikant oftere, end staldkatte, holdes primært som familiedyr. Ejere af huskatte vil derfor oftere, end ejere af staldkatte, være villige til at ofre penge på en neutralisering. For hunkattenes vedkommende koster dét omkring 1000 kr. (Anonym 1997c).

Ifølge beboerne i nærværende undersøgelse lykkes populationsbegrænsningen af huskatte oftere (98 %) end af staldkatte (85 %). Det kan måske undre fordi der, for det første, ikke er flere ejendomme som bruger p-piller til huskatte end til staldkatte, og for det andet, fordi der procentvis er flere ejendomme hvor der aflives staldkattekillinger og voksne staldkatte, end ejendomme hvor der aflives huskattekillinger og voksne huskatte. Tilgengæld steriliseres huskattene på flere ejendomme end staldkattene. Desuden er p-piller

til staldkatte sandsynligvis forbundet med en større usikkerhed end p-piller til huskatte, fordi staldkatte oftere end huskatte er sky og det derfor ikke er så nemt at kontrollere om alle hunkatte har fået ugens pille. At staldkattene er sky besværliggør desuden aflivning af staldkattekillinger, fordi de skjuler deres killinger. Så på trods af at 51 % af ejendommene med staldkat afliver staldkattekillinger, i modsætning til at 21 % af ejendommene med huskat afliver huskattekillinger, kan der være en større andel af staldkattekillingerne der overlever og som husstandens beboere først opdager når de har fået øjne. Det kan være grunden til, at ejendomme med staldkattekillinger hyppigere end ejendomme med huskattekillinger dels overlader aflivningen af killingerne til dyrlægen, dels afliver voksne katte. Det faktum at staldkatte oftere, end huskatte, får lov at beholde én enkelt killing pr. kuld, kan også være medvirkende til at populationsbegrænsningen oftere mislykkes på ejendomme med staldkatte, end på ejendomme med huskatte.

Aflivning af voksne katte er generelt ikke nogen hyppigt brugt metode til at regulere kattebestanden. På i alt 17 ejendomme hvor der aflives voksne katte, sker det i de 15 tilfælde lovligt vha. dyrlæge (4 katte) og personer med jagttegn (11 katte). De resterende 2 ejendomme afliver selv deres katte, hhv. drukner og gasser med udstødningsgas, hvilket i øvrigt er ulovligt (Bekendtgørelse om slagtning og aflivning af dyr, kap. 4, § 13, 1994). Den hyppigste aflivningsmetode af killinger, på såvel ejendomme med huskatte som ejendomme med staldkatte, er at slå killingens hoved mod et hårdt underlag eller kant. På 10 % af nærværende undersøgelses ejendomme med huskatte og på 10 % af ejendommene med staldkatte druknede beboeren killingerne. Der blev ikke fundet nogen ejendomme hvor man gassede killingerne. Drukning af killinger er forbudt ved lov (Bekendtgørelse om slagtning og aflivning af dyr, kap. 4, § 13, 1994; Dyreværnslov, kap 2, § 13, 1993). Ulovlige metoder til aflivning blev altså, for killingernes vedkommende, taget i brug på såvel 10 % af samtlige ejendommene med huskat (drukning) som på 10 % af samtlige ejendommene med staldkat (drukning), og for de voksne kattes vedkommende på 12 % af ejendommene med staldkat (drukning og gasning). Der var ikke registreret brug af ulovlige aflivningsmetoder for voksne huskatte, men materialet er også meget lille, kun 5 husstande. Ved aflivning af kattekillinger ved skydning og ved at slå killingernes hoved mod et hårdt underlag indtræder døden med det samme, såfremt den der udfø-

rer handlingen har håndelaget, hvorfor der ikke kan indvendes noget dyreværnsmæssigt mod disse aflivningsformer. Der kan være risiko for at svarpersonen ikke har angivet aflivningsmetode i overensstemmelse med virkeligheden. Specielt hvis svarpersonen har været bekendt med, at det er ulovligt at drukne katte og killinger. Det tyder dog på at svarpersonerne har svaret ærligt. Der er nemlig procentvis flere svarpersoner, som svarer, at de drukner katte/killinger, end svarpersoner der kender nogle, der drukner katte/killinger og som ikke selv drukner katte.

Tilsyneladende er det af stor betydning for befolkningen på landet, at de selv kan aflive deres killinger, idet 40 % har angivet dette som den primære årsag til valg af aflivningsmetode. Gammel vane spiller dog også en stor rolle, idet 21 % afliver killingerne på samme måde som de altid har gjort. Det var mere udbredt blandt ejendomme med staldkatte, at lade katten beholde én enkelt killing end blandt ejendomme med huskatte. På 60 % af samtlige ejendomme med huskat(te) aflives hele huskattens kuld, mens dette kun sker for staldkattene på 27 % af ejendommene. På dette punkt behandles de to typer katte altså forskelligt. Dette afspejler nok at der muligvis er en større omsætning i kattebestanden på en gård end i et parcelhus og at der på gården derfor jævnligt skal lægges nye katte til. Desuden betyder én kat fra eller til ikke det store på en gård, i modsætning til hvis man kun har én kat, passer og plejer den, har den liggende i sin seng og fodrer den udelukkende med whiskas. Det er imidlertid også sandsynligt at landmænd gennem generationer har opnået samme erfaring som Turner & Meister (1988), at hunkatte med killinger er de mest effektive musejægere.

De to typer katte behandles også forskelligt hvad angår tilbøjeligheden til at hjælpe kattene ved sygdom og til at forebygge sygdom. Husstande med huskatte hjælper hyppigere deres katte ved sygdom (75 %) end husstande med staldkatte (50 %). Resultatet bygger på små tal, fordi kun få beboere på husstande med katte har registreret sygdom blandt kattene. Dertil skal imidlertid lægges de katte, muligvis mange, der bliver syge uden at ejendommens beboer ved det. Husstande med huskatte vaccinerer desuden hyppigere deres katte end husstande med staldkatte, hvilket medfører at kattesyge er observeret oftere på ejendomme med staldkatte end på ejendomme med huskatte. Det er dog stadig

kun på 27 % af samtlige ejendomme med huskatte, hvor huskattene er vaccinerede, mod at staldkattene er vaccinerede på 6 % af samtlige ejendomme med staldkatte. Den aktuelle sundhedstilstand blandt hus- og staldkatte er, ifølge svarpersonen, god på hhv. 96 % og 95 % af de besøgte ejendomme.

Foderstanden af hus- og staldkatte var, ifølge beboerne, god på hhv. 97 % og 98 % af ejendommene. Dette afspejler sandsynligvis det faktum, at beboerne i området villigt fodrer katte. På 98 % af samtlige ejendomme med kat(te) fodres ejendommens katte dagligt og på 42 % af samtlige ejendomme med kat(te) og 15 % af samtlige ejendomme uden kat fodrer beboeren fremmede katte. Disse fremmede katte kan høre til på en ejendom i nærheden, men kan også være herreløse og kan evt. spise på flere ejendomme. Denne tilførsel af føde reducerer vigtigheden af fangst af naturlige fødeemner, specielt for drægtige og diegivende hunkatte (Laundré 1977). Desuden muliggør det at kattene kan leve i grupper, bestående af beslægtede hunkatte og deres afkom, med langt større bestandstætheder end når føden er mere knap (Liberg & Sandell 1988). Bestandstætheden af katte er altså en følge af mængden af føde (Liberg & Sandell 1988). En effektiv metode til at mindske bestanden af katte vil derfor være, at reducere mængden af den tilgængelige føde. Det vil dog næppe være muligt at praktisere, eftersom folk fodrer kattene, fordi de ikke kan få over deres dyreværnsvenlige hjerter at lade være, når de ser en kat der er sulten, katteskuespil eller ej. Desuden har de jo lige denne her rest fra aftensmåltidet, som de ikke kan lide at smide ud. Mere realistisk er det at hindre en bestandstilvækst af katte gennem en reduktion af den tilgængelige føde, når der ad anden vej er blevet tyndet ud i bestanden.

Den tætte bestand af katte er i og for sig ikke nødvendigvis et problem for kattene. Men når en stor del af beboerne på landet fodrer andre katte end deres egne, opstår der problemer omkring ejerforholdet til katten. Der er retspraksis, fra Fakse ret, for at man har ansvaret for en kat man har fodret gennem en periode. Dermed er man også forpligtet til at overholde dyreværnsloven. Dvs. uanset om man opfatter sig selv som ejer af katten, kan man ikke stoppe med at fodre katten, undlade at hjælpe den ved sygdom o.s.v. Risikoen for at disse katte overlades til sig selv er reelt til stede, fordi folk ikke føler sig for-

pligtet til fortsat at tage vare på katten, domspraksis eller ej. Dyrenes Beskyttelse opererer med ovennævnte definition af en ejerkat og foreningens politik for ejerkatte er at få kattens ejer (enkeltperson, boligforening el.lign.) til at tage ansvar for den. I de tilfælde hvor ejerforholdet ikke ligger fast og det formodes at katten vil blive nødstedt, forsøges det at få kommunen til at løse problemet, og kun i helt særlige tilfælde giver foreningen økonomisk hjælp til aflivning af ejerkatte.

Anbefalinger til Dyrenes Beskyttelse

Af resultaterne i nærværende undersøgelse kan man tolke, at der ikke er store dyreværnsmæssige problemer for katte på landet i Danmark. Men dermed er den hellige grav ikke velforvaret. For katteejerne har ansvaret for den meget store bestand af katte, og for de skader og påvirkninger kattene måtte øve på faunaen. Med andre ord, så har katteejerne har et ansvar, fordi de skaber så stor en bestand af katte, der æder faunaen. Derfor er yderligere viden om kattens antal og levevilkår påkrævet.

Følgende kan anbefales Dyrenes Beskyttelse:
Iværksætte yderligere undersøgelser af kattens antal og levevilkår (se afsnittet "Hvad mangler vi at få belyst og hvordan kan det gøres").

Iværksætte en oplysningskampagne om problemerne omkring fodring af fremmede katte. Dette kan gøres med udgangspunkt i den allerede producerede folder "Tilløber-katte". Der skal gøres et stort benarbejde for at oplyse landbefolkningen om folderen, gennem skrevne og levende medier. Kampagnen skal hovedsagelig rettes mod ikke-landbrug, dvs. beboere på parcelhuse i landområder.

Når/hvis der findes viden om det tilstrækkelige antal katte pr. ejendom, for at holde ejendommen ugeneret af mus og rotter, lave en oplysningskampagne herom rettet mod landbrug, såvel fuldtids- som fritidslandbrug, agerbrug som husdyrbrug. At katte der fodres og neutraliseres er mindst lige så effektive jægere som katte der ikke fodres og neutraliseres. At mindre kattehold muliggør bedre kontakt mellem menneske og hver enkelt kat, med deraf følgende mulighed for udelukkende at fodre egne katte samt for større kontrol

med kattene, dvs. større grad af succes med populationsbegrænsning, behandling ved sygdom o.lign. At få velpassede katte medfører større fornøjelse/færre problemer for ejendommens beboere, i kraft af raskere katte og bedre afkom.

Hvad mangler vi at få belyst og hvordan kan det gøres

Følgende viden er vigtig for den fremtidige forvaltning af katte:
En undersøgelse af bestandstætheden af katte i et landbrugsområde i Jylland. For at undersøge pålideligheden af nærværende undersøgelses estimat af antal katte på landet. Når vi kender estimatets pålidelighed, har vi et godt grundlag for at sammenligne med bestandstætheder af katte i by- og sommerhusområder.

Antallet af katte i byer med mere end 200 indbyggere, opdelt i villa-, lejligheds- og industrikvarterer. Spørgebrev eller rundspørge af antal katte og om disse er øremærkede eller ej for at få en ide om katte med et fast tilhørsforhold til en husstand. Foto-fangst/genfangst og evt. fælde-fangst/genfangst for at få en ide om katte uden fast tilhørsforhold til en husstand.

Antallet af katte i sommerhusområder. Udføres som foto-fangst/genfangst og/eller fældefangst/genfangst. Spørgebrevsundersøgelse uegnet, hvem skal brevene stiles til, ejeren eller lejeren ? Interview uegnet fordi mange kun bebor deres huse kortvarigt, de når ikke at få et indtryk af kattebestanden i området. Desuden er der ved en rundspørge generelt problemer omkring hvorvidt sommerhusgæsterne betragter katten som deres eller naboens.

Vildtlevende kattes bestandstæthed i landområder. Dvs. katte der reproducerer vildtlevende. Kan udføres som en spørgebrevsundersøgelse til f.eks. jægere og landmænd, hvor de bliver spurgt om deres kendskab til katte der reproducerer vildtlevende i f.eks. rævegrave, markskel o.lign.

Det er stadig et åbent spørgsmål hvor de herreløse katte kommer fra. Er det faktum, at befolkningen gerne fodrer fremmede katte, medvirkende til at øge antallet af herreløse katte?

Hvorledes stemmer landbefolkningens estimat af antallet af katte på deres ejendom overens med virkeligheden? Foretages dels som spørgebrevsundersøgelse, dels ved fotomærkning af katte på de implicerede ejendomme.

Hvor mange katte er tilstrækkeligt til at holde en ejendom ugeneret af mus og rotter? Sammenligne musebestandene på ejendomme hvor bestande af katte introduceres, reduceres og forøges. Ejendommene skal som udgangspunkt have pæne, kendte bestande af mus.

Kattepopulationernes påvirkning af vildtlevende byttedyr og af bestandene af vildtlevende rovdyr, f.eks. brud og hermelin, som predator og fødekonkurrent. Såvel den bestandsmæssige som den adfærdsmæssige side bør undersøges.

KONKLUSION

Områdets bestandstæthed af katte, med et fast tilhørsforhold til en husstand, er 16,6 – 20,0 katte / km^2. Mere end hver anden husstand har kat. Bestanden af katte i området er tilsyneladende stabil.

Antallet af katte på landet i Danmark, med fast tilhørsforhold til en husstand, estimeres til minimum en halv million katte.

Antallet af katte på en ejendom afhænger af hvor isoleret ejendommen er placeret og af hvor meget dyrehold der findes på ejendommen. Således havde fuldtidshusdyrbrug flest katte pr. ejendom, nemlig 5,03 katte og parcelhuse i bymæssig bebyggelse færrest katte, nemlig 0,54 katte pr. ejendom.

11 % af samtlige beboere i området mente der var for mange katte. Disse beboere havde dog ikke færre katte på ejendommen og var ikke mere tilbøjelig til at bestandsregulere.

Der er flere katte på ejendomme, hvor kattene holdes som predatorer, end på ejendomme, hvor kattene holdes som familiedyr.

Killinger fødes hovedsagelig forår og sommer. Kuldstørrelsen på disse årstider er ikke større end efterår og vinter. Der er i gennemsnit 3,73 killinger pr. kuld.

Nærværende undersøgelse viser at landboere bekymrer sig om deres katte, idet:

- Kattene fodres på 98 % af samtlige ejendomme. Kattenes aktuelle foderstand og sundhedstilstand er god på mere end 95 % af samtlige ejendomme.
- Antallet af katte reguleres på mere end 70 % af samtlige ejendomme. Reguleringen af stald- og huskatte lykkes på hhv. 85 % og 98 % af samtlige ejendomme.
- Langt hovedparten af katte og killinger aflives dyreværnsmæssigt forsvarligt.

På to områder kan landboerne gøre det bedre.

- Kattene hjælpes ved sygdom kun på 50 % - 75 % af samtlige ejendomme.
- Beboerne bør ophøre med at fodre fremmede katte.

REFERENCER

Anonym, 1989. Større gårde og skove 88/89. Redaktion: Nørgaard, C., Terndrup, H & Serner, E. Kraks Forlag, København, 342 s.

Anonym, 1997a. Landbrugsstatistik 1996. Danmarks Statistik, Danmarks Statistiske Trykkeri, Kbh., 310 s.

Anonym, 1997b. Statistisk Årbog 1997. Danmarks Statistik, Århus Stiftsbogtrykkeri, Århus, 571 s.

Anonym, 1997c. Tænk, no. 7. Forbrugerrådet, Kbh.

Anonym, 1998. Statistisk Efterretning, Befolkning og valg, nr. 18. Danmarks Statistik, Danmarks Statistiske Trykkeri, Kbh., 28s.

Bekendtgørelse om slagtning og aflivning af dyr, kap. 4, § 13. Justitsministeriets bekendtgørelse nr. 1037 af 14. dec. 1994.

Childs, J. E., 1986. Size-dependent predation on rats (*Rattus norvegicus*) by house cats (*Felis catus*) in an urban setting. Journal of Mammalogy 67(1): 196-199.

Christoffersen, M. N., 1984. Brev, telefon, besøg. En vurdering af anvendte dataindsamlingsmetoder. Socialforskningsinstituttet, København, 146 s.

Clausen, B., Rønn, A. & Hviid, I., 1996. Katte uden ejer. Dansk Veterinærtidsskrift 79(12): 536-544.

Dyreværnslov, kap 2, § 13. Lov nr. 386 af 6. juni 1991 som ændret ved lov nr. 183 af 14. april 1993.

Coleman, J.S. & Temple, S.A., 1993. Rural residents´ free-ranging domestic cats: a survey. Wildlife Society Bulletin 21: 381 – 390.

Elton, C. S., 1953. The use of cats in farm rat control. The british journal of animal behaviour 1: 151-155.

Erlinge, S., Frylestam, B, Göransson, G., Högstedt, G., Liberg, O., Loman, J., Nilsson, I. N., Schantz, T. von & Sylvén, M., 1984. Predation on brown hare and ring-necked pheasant populations in southern Sweden. Holarctic Ecology 7: 300-304.

Fitzgerald, B. M., 1988. Diet of demestic cats and their impact on prey populations. Side 123-150 i Turner, D. C. & Bateson, P. (redaktion): The domestic cat. The biology of its behaviour. Cambridge University Press, Beds, Great Britain, 222 s.

Genovesi, P., Besa, M. & Toso, S., 1995. Ecology of a feral cat *Felis Catus* population in an agricultural area of northern Italy. Wildlife Biology 1(4): 233-237.

Jensen, E. & Nielsen, T. S., 1985. Metode og skriftlig fremstilling i samfundsfag. Gyldendal, København, 122 s.

Laundré, J., 1977. The daytime behaviour of domestic cats in a free-roaming population. Animal Behaviour 25: 990-998.

Liberg, O., 1980. Spacing patterns in a population of rural free roaming domestic cats. Oikos 35: 336-349.

Liberg, O., 1984. Food habits and prey impact by feral and house-based domestic cats in a rural area in southern sweden. Journal of Mammalogy 65(3): 424-432.

Liberg, O. & Sandell, M., 1988. Spatial organisation and reproductive tactics in the domestic cat and other felids. Side 83-98 i Turner, D. C. & Bateson, P. (redaktion): The

domestic cat. The biology of its behaviour. Cambridge University Press, Beds, Great Britain, 222 s.

Macdonald, D.W., Apps, P.J., Carr, G.M. & Kerby, G., 1987. Social dynamics, nursing coalitions and infanticide among farm cats (*Felis catus)*. Advances in Ethology, no. 28. Supplement to Ethology. Verlag Paul Parey, Berlin, 66 s.

Madsen, B. L., 1988. Børn. Dyr & Natur. Forlaget Børn og Unge, København, 323 s.

Svensson, S., 1996. Huskattens predation på fåglar i Sverige. Ornis Svecica 6: 127-130.

Turner, D. C. & Meister, O., 1988. Hunting behaviour of domestic cat. Side 111-122 i: Turner, D. C. & Bateson, P. (redaktion): The domestic cat. The biology of its behaviour, 222 s.

Warner, R. E., 1985. Demography and mowements of free-ranging domestic cats in rural Illinois. Journal of Wildlife Management 49(2): 340-346.

BILAG

EJENDOMMEN

PARCELHUS/VILLA M. HAVE (def. 1) — SÆT X
- Enkeltliggende
- mindre klynge huse (2-10 huse)
- bymæssig bebyggelse (11-20 huse)

SOMMERHUS / FRITIDSHUS — SÆT X
- Enkeltliggende
- mindre klynge huse (2-10 huse)
- bymæssig bebyggelse (11-20 huse)

FRITIDSLANDBRUG (def. 2) — SÆT X

Arealstørrelse (ha) — SÆT X
- 2000 m^2 - 5 ha
- over 5 ha

FULDTIDSLANDBRUG (def. 3) — SÆT X

Arealstørrelse (ha) — SÆT X
- 0 - 9,9
- 10 – 29,9
- 30 - ∞

ANDET: Hotel, kro, kursuscenter, golfklub, museum, vandværk

VEDR. LANDBRUGET/FRITIDSLANDB.

DRIFTSFORM — SÆT X
- Agerbrug (inkl.brak)
- Anden planteavl
  - Gartneri
  - Permanente beplantninger
  - Blandet planteavl
- Kvæg mm.
- Anden husdyrhold
  - Svin
  - Fjerkræ
  - Blandet husdyrhold
- Blandet planteavl og husdyrhold

STALDAREAL (M^2) (Def.4)

AREAL DÆKKET AF LØVSKOV (EXCL. PLANTAGE) — sæt X
- ≥ 25 %
- < 25 %

JAGT

	Ja	Nej
Drives der jagt på ejendommen ?.		

Hvem udfører jagten ?. — sæt X
- Selv
- Familie
- Ven
- Udlejet
- Blandet

VEDR. EJENDOMMEN

AFSTAND FRA BEBYGGELSE TIL NÆRMESTE SKEL SÆT X
- ≤ 50 m
- > 50 m

ANTALLET AF KATTE PÅ EJENDOMMEN, HVOR MANGE HAR MULIGHED FOR AT STREJFE, OG HVOR MEGEN KONTAKT MAN HAR MED DEM.

ANTAL KATTE	antal	Han	Hun
Hvor mange katte holder til på din ejendom?.			
Hvad er det største antal katte du har haft Samtidig inden for de sidste 12 måneder ?.			
Hvor mange katte har derudover vist sig på din ejendom i alt de sidste 12 måneder ?.			

	Ja	Nej	Nogle
Kender du de katte (dvs. ovennævnte) (fx. naboens/genboens)			
Hvor mange af dem ?.			

Hvordan ser de ud:

Hvordan ser de katte ud, som du ikke ved hvor kommer fra:

FORHOLD TIL KATTENE	antal	Han	Hun
Hvor mange katte kan der kæles for (def. 5)?.			

Hvor mange katte fodrer I (def. 6)?
- ingen
- nogle — Antal:
- Alle egne
- Alle også fremmede

	antal	Han	Hun
Hvor mange af kattene anser du som hørende til på din ejendom ?.			
Hvor mange katte kommer indendørs (hus)(def. 7) ?.			

Udfyldes af interviewer på baggrund af besvarelserne af ovenstående spørgsmål !.

ANTAL KATTE (def. 8-11)

	Huskat	Staldkat	SPF-kat	Strejferkat	I alt
Han					
Hun					
Ialt					

Er der nogen speciel grund til at I har katte?	Huskat sæt x	Staldkat Sæt x	SPF-kat sæt x
Rotte/musebekæmpelse			
Kæledyr			
De er her bare/har altid været her			
Andet			

	J	N	?
Er der for mange katte i området?.			

Hvorfor ?. — Huskat sæt x
- De tager småfuglene
- De tager det jagtbare vildt
- De er for dyre i foder
- De sviner
- Andet

Bemærkninger:
a: adult.
j: juvenil.
J: ja.
N: nej.
?: ved ikke.

Beboerne på de landbrug der lå udenfor forsøgsområdet, blev kun bedt om at besvare spørgsmålene indtil den firkantede kasse med dobbelt streg omkring.

Bilag 1. Spørgeskema brugt til interview af beboere i forsøgsområdet. "Ekstra landmænd" kun interviewet vedr. spørgsmålene til og med kassen med dobbelt streg omkring.

Bilag 1…fortsat.

BEGRÆNSES ANTALLET AF KATTE, HVORFOR, HVORDAN OG ER DET EFFEKTIVT ?.

POPULATIONSBEGRÆNSNING

	Huskat Ja	Huskat Nej	Staldkat Ja	Staldkat Nej	SPF-kat Ja	SPF-kat Nej	Strejferkat Ja	Strejferkat Nej
Gør I noget for at her ikke skal komme flere katte?.								
Vil I gøre det engang i fremtiden?.								

Hvordan ?. Gør I / vil I gøre .	Huskat sæt x	Staldkat sæt x	SPF-kat sæt x	Strejferkat sæt x
P-piller				
Neutralisering				
Hankat				
Hunkat				
Aflivning af kattekillinger (def. 12)				
Hankilling				
Hunkilling				

Hvor mange killinger beholder I pr. kuld: 0 / 1 / <1

Aflivning af voksne	sæt x	sæt x	sæt x	sæt x
Hankatte				
Hunkatte				

Hvordan foregår aflivningen?.	Huskat a	Huskat j	Staldkat a	Staldkat j	SPF-kat A	SPF-kat j	Strejferkat a	Strejferkat j
Skydning								
Gasning								
Drukning								
Slår kattens hoved mod hård kant, mur, gulv								
Dyrlæge								
Andet								

Synes I aflivningsmetoden er: God / Dårlig / Ved ikke

Hvorfor har I valgt den aflivningsmetode I bruger?.

	SÆT X
Anbefalet af dyrlægen	
Billigst	
Kan vi udføre selv	
Sådan har vi altid gjort	
Ved ikke	
Andet, beskriv	

	Huskat J	N	?	Staldkat J	N	?	SPF-kat J	N	?	Strejferkat J	N	?
Hvis I bruger populations-begrænsning på hunkatte, lykkes det så at begrænse antallet af katte?.												

Kender I nogen der afliver deres kattekillinger ved drukning? (exkl. dem der drukner selv): Ja / Nej / Ved ikke

SUNDHEDSTILSTAND OG OM MAN BEHANDLER SINE KATTE

	Huskat J	N	?	Staldkat J	N	?	SPF-kat J	N	?
Synes du dine katte ser raske ud ?.									
Synes du dine katte er i god foderstand ?.									

	Huskat Ja	Huskat Nej	Staldkat Ja	Staldkat Nej	SPF-kat Ja	SPF-kat Nej
Har dine katte været syge ?.						

Hvilke sygdomme	Huskat sæt x	Staldkat sæt x	SPF-kat sæt x
Influenza, kattesyge (næseflåd, nysen, hoste, Spytflåd/savl, opkastninger, diarre)			
FIV, FeLV (vægttab, nedsat ædelyst, sløvhed, Opkastninger, håntab)			

Var flere dyr syge samtidig: Ja / Nej / Ved ikke

Hvilke dyr var hovedsagelig syge (def. 12): Killinger / Voksne / Ved ikke

Hvordan forløb sygdommen (def. 13): Akut / Længerevarende / Ved ikke

Er kattene blevet behandlet ?.	Huskat	Staldkat	SPF-kat
Ja			
Nej			
Ved ikke			
Husker ikke			

	Huskat Ja	Huskat Nej	Staldkat Ja	Staldkat Nej	SPF-kat Ja	SPF-kat Nej
Er kattene blevet aflivet ?						

Hvor mange af kattene bliver vaccineret (def. 15)?.	Huskat sæt x	Staldkat sæt x	SPF-kat sæt x
Alle			
Nogle			
Ingen			

FORMERING

Hvornår fik kattene killinger sidste år?.
Skriv årstid (def. 15)

Er det altid på den årstid ?.: Ja / Nej / Ved ikke

Hvor mange killinger fik katten(e) pr. kuld sidste år ?.: antal

Får hunkattene killinger samtidig eller forskudt (def. 16)?.: Samtidig / forskudt / ved ikke

Hvor mange killinger får dine katte normalt hver = gennemsnit her på stedet med de katte der holder til her ?: antal

Er der forskel på kuldstørrelse alt efter fødselstidspunkt på året ?.: Ja / Nej / Ved ikke

Bilag 1…fortsat.

Definitioner

1. Parcelhus: Alle hustyper (villa, parcelhus, rækkehus) der ikke har mere jord end hvad må betragtes som have. Dvs. der ejes ikke landbrugsjord og drives ikke landbrug fra/på ejendommen. Opdeles i enkeltliggende parcelhus, parcelhus i klynge og parcelhus i bymæssig bebyggelse. Enkeltliggende parcelhus: Op til 3 parcelhuse, hvor afstanden mellem husene to og to er mindre end 200 meter, som er beliggende mere end 200 meter fra øvrige parcelhuse. Mindre klynge parcelhuse: En sammenhængende bebyggelse, der på opgørelsestidspunktet har 4-10 huse; at bebyggelsen er sammenhængende vil sige, at afstanden mellem husene ikke overstiger 200 meter, medmindre afbrydelsen skyldes offentlige anlæg, kirkegårde o.lign. (Boliger på Valsølille Gården inkluderet her). Parcelhus i bymæssig bebyggelse: En sammenhængende bebyggelse, der på opgørelsestidspunktet har 11 huse eller flere; at bebyggelsen er sammenhængende vil sige, at afstanden mellem husene ikke overstiger 200 meter, medmindre afbrydelsen skyldes offentlige anlæg, kirkegårde o.lign.

2. Fritidslandbrug: Beboelser, uanset beliggenheden i forhold til andre boliger (bymæssig bebyggelse eller fritliggende), hvortil der er knyttet landbrugsjord eller jord der bruges til fritidslandbrug og altså eksl. beboelser hvortil der blot findes almindelige haver. Drives på fritids- eller deltids-basis. Tilforpagtede og tillejede arealer medregnes ikke under bedriften, mens bortforpagtede eller bortlejede arealer betragtes som hørende til bedriften. Opdeles i fritidsagerbrug og fritidshusdyrbrug. Fritidsagerbrug: Landbrug hvor agerbrug, braklægning, gartneri, permanente beplantninger, anden planteavl og blandet planteavl udgør hele brugets driftsform. Der kan holdes husdyr til landbofamiliens eget forbrug (ernæring og forlystelse), men aldrig i erhvervsøjemed. Holdet af husdyr til ernæring og forlystelse skønnedes ikke at udgøre en væsentlig del af landbruget, f.eks. optage størstedelen af jorden. Fritidshusdyrbrug: Landbrug hvor kødkvæg, malkekvæg, svin, fjerkræ, minkavl, hestehold og/eller blandet husdyrhold udgør brugets driftsform eller skønnedes at udgøre en reel del heraf. Inkluderer de brug hvor husdyrene holdes i erhvervsøjemed eller til landbofamiliens eget forbrug og hvor husdyrene skønnedes at udgøre en væsentlig del af landbruget, f.eks. ved at optage størstedelen af jorden.

3. Fuldtidslandbrug: Beboelser, uanset beliggenheden i forhold til andre boliger (bymæssig bebyggelse eller fritliggende), hvortil der er knyttet landbrugsjord, dvs. excl. beboelser hvortil der blot findes almindelige haver. Drives på fuldtids-basis. Tilforpagtede og tillejede arealer medregnes ikke under bedriften, mens bortforpagtede eller bortlejede arealer betragtes som hørende til bedriften. Opdeles i fuldtidsagerbrug og fuldtidshusdyrbrug, se fritidslandbrug for definition.

4. Staldareal: Ejendommens bebyggede areal excl. beboelse, dvs. inkl. lade, lo osv.

5. Katte der kan kæles for: Er katte der kan tages op af mennesker og kæles med. Katte som blot kan kæles med i forbindelse med fodring er ikke inkluderet.

6. Kattene fodres: Katten(e) har daglig adgang til foder. Eksklusiv de katte der er henvist til at selvbetjene sig på mink- og/eller grisefoderet.

7. Katte der kommer indendørs i boligen: Er katte der får lov til at være indenfor, ligge indenfor. Katte som blot kommer indenfor i køkken, bryggers el.lign. sted i forbindelse med fodring er ikke inkluderet.

8. Huskat: Katte der kommer indenfor i boligen, se def. 7.

9. Staldkat: Katte der ikke kommer indenfor i boligen, udover køkkenet, bryggers eller lign. i forbindelse med fodring.

10. SPF-kat: Katte der holdes i stalde med SPF-svin og som hindres i at komme ud.

11. Strejferkat: Katte uden fast tilhørsforhold til den besøgte husstand.

12. Killinger: Definitionen af killinger er ikke konsekvent, men er forskellig for spørgsmålene vedr. sygdom og aflivning. Aflivning: Killinger defineret af svarpersonen selv, dvs. det varierer fra individer der endnu ikke har fået øjne til individer som er mindre afhængige af moderen. Sygdom: Ved killinger (juvenile) forstås katte i 0-7 måneders alderen begge måneder inklusiv. Ved voksne katte (adult) forstås alle katte på 8 måneder eller ældre .

13. Akut sygdom: Op til to uger (incl.). Længerevarende sygdom: Mere end to uger.

14. Vaccineres: Bliver vaccineret jævnligt, dvs. er blevet vaccineret gentagne gange. Katte som kun er vaccineret én gang inkluderes ikke.

15. Forår: marts, april, maj. Sommer: juni, juli, august. Efterår: september, oktober, november. Vinter: december, januar, februar

16. Samtidig nedkomst: To eller flere hunkatte på samme husstand har fået killinger indenfor 7 dage. Forskudt nedkomst: Mere end 7 dage imellem at to hunkatte på samme husstand har fået killinger.

ZOOLOGISK MUSEUM
Københavns Universitet

19/12-1997.

Til !

Vedr. Projekt Landkat.

Antallet af katte på landet i Danmark er stigende. Men vi ved ikke nøjagtigt hvor mange katte der er. Vi ved heller ikke hvad husejerene på landet synes om kattene. Nogle er måske glade for de mange katte, mens andre føler sig generet af dem.

En nærmere undersøgelse af antallet af katte og husejernes holdning til katte på den egn hvor De bor er i gang. Undersøgelsen hedder Projekt Landkat og er et samarbejde mellem Zoologisk Museum i København og Dyrenes Beskyttelse. I forbindelse med undersøgelsen er jeg interesseret i at høre Deres mening om katte.

Jeg vil derfor tillade mig at ulejlige Dem med et kort besøg sidst i uge 2 (5.-9. januar) eller begyndelsen af uge 3 (12.-16. januar) i det nye år.

Jeg håber De vil deltage i undersøgelsen. Det er nemlig meget vigtigt, at jeg får snakket med så mange beboere på egnen som muligt.

På forhånd tak for hjælpen i det nye år.

Med venlig Hilsen

M. Ujvári

Marianne Ujvári
Cand. scient. i biologi

Zoological Museum, University of Copenhagen

Universitetsparken 15
DK 2100 København Ø
Danmark

Tel: + 45 35 32 10 00
Fax: + 45 35 32 10 10
Telex: 22221 unicop dk

Bilag 2. Skrivelse sendt til samtlige beboere i forsøgsområdet ca. 1 uge før besøget.

ZOOLOGISK MUSEUM
Københavns Universitet

Dato ________ Kl. _____

Til ________________________ !

Vedr. Projekt Landkat.

Jeg har været her, for at høre jeres holdning til katte på jeres ejendom.

Jeg vil tillade mig at komme forbi igen dag d. / , kl. ca. .

Jeg håber I da vil være hjemme. Det er nemlig meget vigtigt for undersøgelsen, at jeg får snakket med så mange beboere her på egnen som muligt.

Såfremt I foretrækker et andet tidspunkt end ovennævnte, kan I lægge en besked herom hos Dyrenes Beskyttelse på telefon nr. **31 22 32 22** (hverdage mellem kl. 8.30 og 15.30). Opgiv venligst gade-navn og nummer. Jeg vil så ringe tilbage hurtigst muligt for at aftale et bedre tidspunkt. Hverdage efter kl. 15.30 samt lørdage og søndage kan jeg kontaktes på mobil telefon nr. **30 46 14 16**.

På forhånd tak.

Med venlig Hilsen

Marianne Ujvári
Cand. scient. i biologi

Zoological Museum, University of Copenhagen

Universitetsparken 15
DK 2100 København Ø
Danmark

Tel: + 45 35 32 10 00
Fax: + 45 35 32 10 10
Telex: 22221 unicop dk

Bilag 3. Skrivelse til de beboere der ikke blev træffet hjemme, vedr. aftale af et nyt tidspunkt for besøget.
