# Supplementary material for: Changes in management of owned cats in the countryside – A comparison of results from surveys undertaken in the same rural area of Denmark in 1998 and 2022
Source: PLoS One. 2025 Feb 19;20(2):e0316704. doi: 10.1371/journal.pone.0316704 (PMC11838870; doi:10.1371/journal.pone.0316704)
Supplement: S2 File — (PDF) [file pone.0316704.s002.pdf]

Bilag 8 - Spørgeskema papirversion 2022

Spørgeskema, Projekt Landkat vol. 2

1. Skriv adresse og nummer fra masterliste:

2. Status:
- ☐ Ikke besøgt
- ☐ Har besvaret
- ☐ Ønsker ikke at deltage
- ☐ Ikke hjemme, besøg igen
- ☐ Send skema til email
- ☐ Ring til
- ☐ Aftal besøg på forhånd

3. Ligger ejendommen indenfor forsøgsområdet?
☐ Ja ☐ Nej

Ejendommen.
Efterfølgende spørgsmål omhandler hvilken type ejendom deltageren bor i.

4. Hvilken type ejendom bor du i?
- Parcelhus/villa m. have
- Sommerhus/fritidshus
- Fritidslandbrug
- Fultidslandbrug
- Andet. F.eks Hotel, kro, kursuscenter, golfklub, museum, vandværk.________________________

5. Hvad er afstanden fra bebyggelse til nærmeste skel?
☐ ≤50m ☐ ≥50

6. Hvilken type parcelhus/villa m. have er det? (stilles kun til beboere som tidligere har markeret parcelhus)
- ☐ Enkeltliggende
- ☐ Mindre klyngehuse (2-10 huse)
- ☐ Bymæssig bebyggelse (11-20 huse)

7. Hvilken type sommerhus/fritidshus er det (stilles kun til beboere som tidligere har markeret sommerhus/fritidshus)
- ☐ Enkeltliggende
- ☐ Mindre klyngehuse (2-10 huse)
- ☐ Bymæssig bebyggelse (11-20 huse)

8. Hvor stort et areal (ha) er dit fritidslandbrug (stilles kun til beboere som tidligere har markeret fritidslandbrug)
- ☐ Arealstørrelse 2000 m2 - 5 ha
- ☐ Arealstørrelse over 5ha

9. Hvor stort et areal (ha) er dit fultidslandbrug (stilles kun til beboere som tidligere har markeret fultidslandbrug)
- ☐ Arealstørrelse 0 - 9,9 ha
- ☐ Arealstørrelse 10 - 29,9 ha
- ☐ Arealstørrelse over 30 ha

10. Hvad er jeres driftsform? (stilles kun til beboere som tidligere har markeret fultids/fritidslandbrug)
- ☐ Planteavl (korn, grøntsager, skov mm)
- ☐ Kvæg
- ☐ Svin
- ☐ Fjerkræ
- ☐ Blandet husdyrhold:__________________
- ☐ Andet husdyrhold: __________________
- ☐ Blandet planteavl og husdyrhold

11. Hvad er staldarealet i m2? (stilles kun til beboere som tidligere har markeret fultids/fritidslandbrug): _______m2

12. Efterfølgende spørgsmål drejer sig om antallet af katte på ejendommen

13. Hvor mange katte har du, som får lov til at komme indenfor i din bolig? (A. antal huskatte)
- Antal hanner _____________
- Antal hunner _____________
- Antal ukendt køn _____________
- Antal i alt _____________
- Ingen ☐ Ved ikke

14. Hvor mange katte har du, som kun opholder sig indendørs? (antal strikte inde-huskatte)
- Antal hanner _____________
- Antal hunner _____________
- Antal ukendt køn _____________
- Antal i alt _____________
- Ingen ☐ Ved ikke

15. Hvad er den primære grund til at du/I har huskatte?
- ☐ Rotte/mus bekæmpelse
- ☐ Kæledyr
- ☐ De er her bare / har altid været her
- ☐ Andet. Hvilket: ______________________
- ☐ Ved ikke

16. Fodrer du dine huskatte?
- ☐ Ja, mindst en gang om dagen
- ☐ Ja, nogle gange (ugentligt)
- ☐ Nej

17. Hvis ja, hvad fodrer du dem med?
- ☐ Kattefoder (tør/vådfoder)
- ☐ Andet foder / madrester
- ☐ Begge dele

18. Hvor mange katte har du, som kun opholder sig udenfor (evt ind i bryggers ifbm fodring), dvs staldkatte/udekatte? (B. antal staldkatte/udekatte)
- Antal hanner _____________
- Antal hunner _____________
- Antal ukendt køn _____________
- Antal i alt _____________
- Ingen ☐ Ved ikke

19. Hvor mange af staldkattene er tamme og hvor mange er sky/"vilde"? (Stilles kun hvis deltageren markerer at de har staldkat/udekat)
- Tamme (skriv antal): __________
- Sky/vilde (skriv antal): __________

20. Hvad er den primære grund til at du/I har staldkatte/udekatte?
- ☐ Rotte/mus bekæmpelse
- ☐ Kæledyr
- ☐ De er her bare / har altid været her
- ☐ Andet. Hvilket: ______________________
- ☐ Ved ikke

21. Fodrer du dine staldkatte/udekatte

- ☐ Ja, mindst en gang om dagen
- ☐ Ja, nogle gange (ugentligt)
- ☐ Nej

22. Hvis ja, hvad fodrer du dem med?
- ☐ Kattefoder (tør/vådfoder)
- ☐ Andet foder / madrester
- ☐ Begge dele

23. Hvor mange katte kommer på din ejendom, som du ved har et andet hjem? f.eks. naboens. (C. antal nabokatte)
- Antal hanner ______________
- Antal hunner ______________
- Antal ukendt køn ______________
- Antal i alt ______________
- Ingen ☐ Ved ikke

24. Fodrer du naboens katte?
- ☐ Ja, mindst en gang om dagen
- ☐ Ja, nogle gange (ugentligt)
- ☐ Nej

25. Hvis ja, hvad fodrer du dem med?
- ☐ Kattefoder (tør/vådfoder)
- ☐ Andet foder / madrester
- ☐ Begge dele

26. Hvor mange katte kommer på din ejendom, som du ved eller tror ikke har et hjem (strejferkatte, hjemløse katte) (D. antal strejferkatte)
- Antal hanner ______________
- Antal hunner ______________
- Antal ukendt køn ______________
- Antal i alt ______________
- Ingen ☐ Ved ikke

27. Hvor mange af strejferkattene er tamme og hvor mange er sky/"vilde"? (stilles hvis deltagerene har markerer at der er strejfekatte på ejendommen)
- Tamme (skriv antal): ___________
- Sky/vilde (skriv antal): ___________

28. Fodrer du strejferkattene?
- ☐ Ja, mindst en gang om dagen
- ☐ Ja, nogle gange (ugentligt)
- ☐ Nej

29. Hvis ja, hvad fodrer du dem med?
- ☐ Kattefoder (tør/vådfoder)
- ☐ Andet foder / madrester

30. Synes du at der er for mange katte i området?
☐ Ja ☐ Nej ☐ Ved ikke

31. Hvorfor synes du at der er for mange katte? (stilles kun hvis der svares ja ovenfor)
- ☐ De tager småfuglene
- ☐ De tager det jagtbare vildt
- ☐ De er for dyre i foder
- ☐ De sviner
- ☐ Andet, skriv: _________________

Populationsbegrænsing (hvorfor, hvordan og er det effektivt?)

33. Gør du/I noget for at begrænse antallet af:

Huskatte (spørgsmål 33)			Staldkatte (spørgsmål 37)			Strejferkatte (spørgsmål 41)		
Ja	Nej	Ved ikke	Ja	Nej	Ved ikke	Ja	Nej	Ved ikke

34. Hvis ja til spørgsmål 33: Hvad gør du/I? for hhv huskatte, staldkatte, strejferkatte

Metode:		Huskat (sp.34)	Staldkat (sp.38)	Strejferkat (sp.42)
P-piller	P-piller			
Neutralisere	Hankatte			
	Hunkatte			
Aflive killinger	Hankillinger			
	Hunkillinger			
	Ukendt køn			
Aflive voksne	Hankatte			
	Hunkatte			
	Ukendt køn			

35. Hvis nej til spørgsmål 33: Vil du/I gøre det i fremtiden? for hhv. huskatte, staldkatte, strejferkatte

Huskatte (sp.35)			Staldkatte (sp.39)			Strejferkatte (sp.43)		
Ja	Nej	Ved ikke	Ja	Nej	Ved ikke	Ja	Nej	Ved ikke

36. Hvis ja til spørgsmål 35: Hvad vil du /I gøre? for hhv huskatte, staldkatte, strejferkatte

Metode:		Huskat (sp.36)	Staldkat (sp.40)	Strejferkat (sp.44)
P-piller	P-piller			
Neutralisere	Hankatte			
	Hunkatte			
Aflive killinger	Hankillinger			

	Hunkillinger			
	Ukendt køn			
Aflive voksne	Hankatte			
	Hunkatte			
	Ukendt køn			

45. Har du nogensinde været i en situation, hvor du/I skulle aflive en kat?

☐ Ja, mine huskatte ☐ Ja, mine staldkatte ☐ Ja, strejferkatte ☐ Nej ☐ Ved ikke

46. Hvis ja, hvordan foregik det? for hhv. huskatte, staldkatte, strejferkatte

Metode:	Huskatte (sp.46)		Staldkatte (sp.47)		Strejferkatte (sp.48)	
	voksne	unge	voksne	unge	voksne	unge
Skydning						
Gasning						
Drukning						
Slår hovedet mod hårdt underlag						
Dyrlæge						
Andet						

49 - 54. (for hver afkrydset mulighed ovenfor, stil spørgsmål:) Hvorfor har I valgt den aflivningsmetode, I bruger?

Metode	Årsag til valg af aflivningsmetode						
	Anbefalet af dyrlæge	Billigst	Kan selv udføre	Bedst for katten	Sådan har vi altid gjort	Ved ikke	Andet, beskriv.
Skydning (sp.49)							
Gasning (sp.50)							
Drukning (sp.51)							
Slår hovedet mod hårdt underlag (sp.52)							
Dyrlæge (sp.53)							
Andet (sp.54)							

Sundhedstilstand og om man behandler sine katte

56, 57, 58.

Spørgsmål:	Huskatte				Staldkatte				Strejferkatte			
	Ja, alle	Ja, en / flere	Nej, ingen	Ved ikke	Ja, alle	Ja, en / flere	Nej, ingen	Ved ikke	Ja, alle	Ja, en / flere	Nej, ingen	Ved ikke
Synes du dine katte ser raske ud? (sp.56)												
Synes du dine katte er i god foderstand? (sp.57)												
Har dine katte været syge de sidste 12 mdr? (sp.58)												

59. Hvis ja til sp.58: Hvilke symptomer har de haft? hhv. huskatte, staldkatte, strejferkatte.

Huskatte (sp.59)	Staldkatte (sp.67)	Strejferkatte (sp.75)
• Næseflåd	• Næseflåd	• Næseflåd
• Nysen	• Nysen	• Nysen
• Hoste	• Hoste	• Hoste
• Savl	• Savl	• Savl
• Opkast	• Opkast	• Opkast
• Diarre	• Diarre	• Diarre
• Vægttab	• Vægttab	• Vægttab
• Nedsat ædelyst	• Nedsat ædelyst	• Nedsat ædelyst
• Sløvhed	• Sløvhed	• Sløvhed
• Andet, beskriv:	• Andet, beskriv:	• Andet, beskriv:
• Kendt/diagnosticeret sygdom:	• Kendt/diagnosticeret sygdom:	• Kendt/diagnosticeret sygdom:

60. Er kattene blevet behandlet i forbindelse med deres sygdomsforløb?

	Huskatte (sp.60)	Staldkatte (sp.68)	Strejferkatte (sp.76)
Ja			

Nej			
Ved ikke			

61. Blev kattene aflivet i forbindelse med deres sygdomsforløb?

Huskat (sp.61)			Staldkat (sp.69)			Strejferkat (sp.77)		
Ja	Nej	Husker ikke	Ja	Nej	Husker ikke	Ja	Nej	Husker ikke

62. Bliver kattene vaccineret?

Huskat (sp.62)			Staldkat (sp.70)			Strejferkat (sp.78)		
Ja, alle	Ja, nogle	Ingen	Ja, alle	Ja, nogle	Ingen	Ja, alle	Ja, nogle	Ingen

63. Hvis ja, hvor tit bliver de vaccineret?

Huskatte (sp.63)	Staldkatte (sp.71)	Strejferkatte (sp.79)
• Hvert år	• Hvert år	• Hvert år
• Mindst hvert 3. år	• Mindst hvert 3. år	• Mindst hvert 3. år
• Aldrig	• Aldrig	• Aldrig
• En gang i alt	• En gang i alt	• En gang i alt

Formering (forår 21 - forår 22, dvs fra marts 21 indtil nu)

80. Fik I killinger i denne periode?
☐ Ja ☐ Nej ☐ Ved ikke

81. Hvor mange kuld fik du/I i denne periode?
- Skriv antal: _____________ ☐ Ved ikke

82. Hvor mange killinger fik katten(e) i alt i perioden?
- Skriv antal: _____________ ☐ Ved ikke

83. Hvor mange killinger beholdt du/I per kuld?
☐ 0 ☐ 1 ☐ >1 ☐ Husker ikke antallet

84. Hvad gør du/I med de overskydende killinger? (nyt spørgsmål)
- ☐ Gav alle til nye hjem
- ☐ Gav nogle til nye hjem
- ☐ Aflivede alle
- ☐ Aflivede nogle
- ☐ Ikke relevant
- ☐ Husker ikke
- ☐ Andet: __________

Nye spørgsmål

85. Boede du på samme adresse i 1998?
☐ Ja ☐ Nej ☐ Ved ikke

86. Har du samme antal katte som i 1998?
- ☐ Har samme antal katte
- ☐ Har flere katte
- ☐ Har færre katte
- ☐ Ved ikke

87. Er dine huskatte mærket (chip og/eller øremærket) og registreret i et af de to danske katteregistre? (stilles kun til personer, som tidligere har markeret, at de har huskat).
- Ja, alle er chipmærket og/eller øretatoveret samt registreret
- Ja, nogle er chipmærket og/eller øretatoveret samt registreret
- Nej
- Ved ikke

88. Er dine staldkatte mærket (chip og/eller øremærket) og registreret i et af de to danske katteregistre? (stilles kun til personer, som tidligere har markeret, at de har staldkat).
- Ja, alle er chipmærket og/eller øretatoveret samt registreret
- Ja, nogle er chipmærket og/eller øretatoveret samt registreret
- Nej
- Ved ikke

89. Kender du/I til den ændring af lovgivningen, som betyder, at man kun kan gøre krav på at eje en kat, hvis den er mærket og registreret?
☐ Ja ☐ Nej ☐ Ved ikke

90. Hvad er din holdning til at neutralisere eller aflive katte i området for at begrænse antallet? (både dine egne, naboens og strejferkatte)
- ☐ OK at aflive overskydende killinger
- ☐ Ikke OK at aflive overskydende killinger
- ☐ OK at aflive voksne katte, hvis der er for mange
- ☐ Ikke OK at aflive voksne katte, hvis der er for mange
- ☐ OK at aflive voksne katte, hvis alternativet er, at de lider
- ☐ Ikke OK at aflive voksne katte, selv hvis alternativet er, at de lider
- ☐ Bedst at forebygge ved at kastrere eller sterilisere katte
- ☐ I tvivl
- ☐ Ved ikke

91. Kommentarfelt
