# Supplementary material for: Changes in management of owned cats in the countryside – A comparison of results from surveys undertaken in the same rural area of Denmark in 1998 and 2022
Source: PLoS One. 2025 Feb 19;20(2):e0316704. doi: 10.1371/journal.pone.0316704 (PMC11838870; doi:10.1371/journal.pone.0316704)
Supplement: S3 File — (PDF) [file pone.0316704.s003.pdf]

Supporting information 3 – Questionnaire, paper version, 2022

Questionnaire, project country cat vol. 2

1. State address and number from master list:

2. Status:
- ☐ Not visited
- ☐ Has answered
- ☐ Does not wish to participate
- ☐ Not home, visit again
- ☐ Send questionnaire by email
- ☐ Call
- ☐ Schedule visit in advance

3. Is the property located within the study area?

☐Yes ☐ No

The property. The following questions revolve around the participant's type of property.

4. Which type of property do you reside in?
- ☐ Detached house/villa with garden
- ☐ Holiday home/weekend cottage
- ☐ Farm, small-scale (hobby)
- ☐ Farm, full-scale
- ☐ Other, e.g. hotel, inn, conference center, golf club, museum, waterworks: __

5. What is the distance between buildings and the nearest boundary?

☐ ≤50m ☐ ≥50

6. Which type of detached house/villa with garden is it? (This question is only asked if the participant has marked detached house.)
- ☐ Single house
- ☐ Small group of houses (2-10 houses)
- ☐ Urban area (11-20 houses)

7. Which type of holiday home/weekend cottage is it? (This question is only asked if the participant has marked holiday home/weekend cottage.)
- ☐ Single house
- ☐ Small group of houses (2-10 houses)
- ☐ Urban area (11-20 houses)

8. How large is the area (in hectares) covered by your small-scale farm? (This question is only asked if the participant has marked farm, small-scale (hobby).)
- ☐ Area size 2000 square meters – 5 hectares
- ☐ Area size above 5 hectares

9. How large is the area (in hectare) covered by your full-scale farm? (This question is only asked if the participant has marked farm, full-scale.)
- ☐ Area size 0 - 9.9 hectares
- ☐ Area size 10 - 29.9 hectares
- ☐ Area size above 30 hectares

10. What type of farming do you do? (This question is only asked if the participant has marked full-scale/small-scale farm.)
- ☐ Plant production (cereal, vegetables, forestry, etc.)
- ☐ Cattle
- ☐ Pigs
- ☐ Poultry
- ☐ Mixed livestock farming:_______________
- ☐ Other livestock farming: _______________
- ☐ Mixed plant production and livestock farming

11. How big is the area of the stable/cowshed/pigsty etc. in square meters? (This question is only asked if the participant has

marked full-scale/small-scale farm.): ________square meters

12. The following questions revolve around the number of cats on the property.

13. How many cats do you have with indoor access to your home? (A. number of domestic cats)

- Number of males ______________
- Number of females ______________
- Number of cats with unknown sex ______________
- Number in total ______________
- None ☐ Don't know

14. How many cats do you have that only stay indoors? (Number of strict indoor domestic cats)

- Number of males ______________
- Number of females ______________
- Number of cats with unknown sex ______________
- Number in total ______________
- None ☐ Don't know

15. What is your primary reason for having domestic cats?

- ☐ Preventing rat/mouse infestation
- ☐ Pets
- ☐ There are just here/have always been here
- ☐ Other, describe: _______________________
- ☐ Don't know

16. Do you feed your domestic cats?

- ☐ Yes, at least once a day
- ☐ Yes, sometimes (weekly)
- ☐ No

17. If yes, what do you feed them?

- ☐ Cat food (dry/wet)
- ☐ Other type of food/leftovers
- ☐ Both

18. How many cats do you have that only live outdoors (possibly in a utility room or the like in connection with feeding), i.e. stable cats/outdoor cats? (B. number of stable cats/outdoor cats)

- Number of males ______________
- Number of females ______________
- Number of cats with unknown sex ______________
- Number in total ______________
- None ☐ Don't know

19. How many of the stable cats are tame, and how many of them are shy/"wild"? (This question is only asked if the participant has marked that they have stable cats/outdoor cats.)

- Tame (state number): ___________
- Shy/"wild" (state number): _______

20. What is your primary reason for having stable cats/outdoor cats?

- ☐ Preventing rat/mouse infestation
- ☐ Pets
- ☐ There are just here/have always been here
- ☐ Other Describe: _______________________
- ☐ Don't know

21. Do you feed your stable cats/outdoor cats?

- ☐ Yes, at least once a day
- ☐ Yes, sometimes (weekly)
- ☐ No

22. If yes, what do you feed them?

- ☐ Cat food (dry/wet)
- ☐ Other type of food/leftovers

- ☐ Both

23. How many cats frequent your property that you know have another home (e.g. belong to the neighbor)? (C. number of neighbor cats)

- Number of males ______________
- Number of females ______________
- Number of cats with unknown sex ______
- Number in total ______________
- None ☐ Don't know

24. Do you feed the neighbor's cats?

- ☐ Yes, at least once a day
- ☐ Yes, sometimes (weekly)
- ☐ No

25. If yes, what do you feed them?

- ☐ Cat food (dry/wet)
- ☐ Other type of food/leftovers
- ☐ Both

26. How many cats frequent your property that you know or suspect do not have a home (stray cats, homeless cats) (D. number of stray cats)

- Number of males ______________
- Number of females ______________
- Number of cats with unknown sex ______
- Number in total ______________
- None ☐ Don't know

27. How many of the stray cats are tame, and how many of them are shy/"wild"? (This question is only asked if the participant has marked having stray cats on their property.)

- Tame (state number): ___________
- Shy/"wild" (state number):________

28. Do you feed stray cats?

- ☐ Yes, at least once a day
- ☐ Yes, sometimes (weekly)
- ☐ No

29. If yes, what do you feed them?

- ☐ Cat food (dry/wet)
- ☐ Other type of food/leftovers

30. In your opinion, are there too many cats in the area?

☐Yes ☐ No ☐ Don't know

31. In your opinion, what problems arise from having too many cats? (This question is only asked if the participant has marked yes to the question above.)

- ☐ They catch small birds
- ☐ They catch huntable game
- ☐ They are too expensive to feed
- ☐ They make a mess
- ☐ Other, describe: _______________

Population control (how, why, and is it efficient?)

33. Do you do anything to limit the population of:

Domestic cats (question 33)			Stable cats (question 37)			Stray cats (question 41)		
Yes	No	Don't know	Yes	No	Don't know	Yes	No	Don't know

34. If yes to question 33: What do you do (with regards to domestic cats, stable cats, and stray cats, respectively)?

Method:		Domestic cats (question 34)	Stable cats (question 38)	Stray cats (question 42)
Birth control hormones	Birth control hormones			
Neutering	Male cats			
	Female cats			
Euthanizing kittens	Male kittens			
	Female kittens			
	Unknown sex			
Euthanizing grown-up cats	Male cats			
	Female cats			
	Unknown sex			

35. If no to question 33: Do you intend to do this in the future (with regards to domestic cats, stable cats, and stray cats, respectively)?

Domestic cats (question 35)			Stable cats (question 39)			Stray cats (question 43)		
Yes	No	Don't know	Yes	No	Don't know	Yes	No	Don't know

36. If yes to question 35: What do you intend to do (with regards to domestic cats, stable cats, and stray cats, respectively)?

Method:		Domestic cats (question 36)	Stable cats (question 40)	Stray cats (question 44)
Birth control hormones	Birth control hormones			
Neutering	Male cats			
	Female cats			
Euthanizing kittens	Male kittens			
	Female kittens			
	Unknown sex			
Euthanizing grown-up cats	Male cats			
	Female cats			
	Unknown sex			

45. Have you ever been in a situation where you had to euthanize a cat?

☐ Yes, my domestic cats ☐ Yes, my stable cats ☐ Yes, stray cats ☐ No ☐ Don't know

46. If yes, how did you handle it (with regards to domestic cats, stable cats, and stray cats, respectively)?

Method:	Domestic cats (question 46)		Stable cats (question 47)		Stray cats (question 48)	
	grown-up cats	young cats	grown-up cats	young cats	grown-up cats	young cats
Shooting						
Fumigation						
Drowning						
Knocking its head against a hard surface						
Veterinarian						
Other						

49.-54. (for every marked option above, ask questions:) Why did you choose the stated method of euthanization?

Method:	Reason for choosing euthanization method						
	Recommended by veterinarian	Cheapest	Can handle it myself	Best for the cat	That is how we always handled it	Don't know	Other, describe:
Shooting (question 49)							
Fumigation (question 50)							
Drowning (question 51)							
Knocking its head against a hard surface (question 52)							
Veterinarian (question 53)							
Other (question 54)							

State of health and veterinary care

56., 57., 58.

Questions:	Domestic cats				Stable cats				Stray cats			
	Yes, all of them	Yes, one or more of them	No, none of them	Don't know	Yes, all of them	Yes, one or more of them	No, none of them	Don't know	Yes, all of them	Yes, one or more of them	No, none of them	Don't know
Do you think your cats look healthy? (question 56)												
Do you think your cats are well fed? (question 57)												
Have your cats been sick within the last 12 months? (question 58)												

59. If yes to question 58: Which clinical signs did they have (with regards to domestic cats, stable cats, and stray cats, respectively)?

Domestic cats (question 59)	Stable cats (question 67)	Stray cats (question 75)
• Nasal discharge	• Nasal discharge	• Nasal discharge
• Sneeze	• Sneeze	• Sneeze
• Cough	• Cough	• Cough
• Drool	• Drool	• Drool
• Vomit	• Vomit	• Vomit
• Diarrhea	• Diarrhea	• Diarrhea
• Weight loss	• Weight loss	• Weight loss
• Reduced appetite	• Reduced appetite	• Reduced appetite
• Drowsiness	• Drowsiness	• Drowsiness
• Other, describe:	• Other, describe:	• Other, describe:
• Known/diagnosed disease:	• Known/diagnosed disease:	• Known/diagnosed disease:

60. Have the cats received medical care in the course of their disease?

	Domestic cats (question 60)	Stable cats (question 68)	Stray cats (question 76)
Yes			
No			
Don't know			

61. Have the cats been euthanized in the course of their disease?

Domestic cats (question 61)			Stable cats (question 69)			Stray cats (question 77)		
Yes	No	Don't remember	Yes	No	Don't remember	Yes	No	Don't remember

62. Do the cats get vaccinated?

Domestic cat (question 62)			Stable cat (question 70)			Stray cat (question 78)		
Yes, all of them	Yes, some of them	None of them	Yes, all of them	Yes, some of them	None of them	Yes, all of them	Yes, some of them	None of them

63. If yes, how often are they vaccinated?

Domestic cats (question 63)	Stable cats (question 71)	Stray cats (question 79)
• Every year	• Every year	• Every year
• At least every 3 years	• At least every 3 years	• At least every 3 years
• Never	• Never	• Never
• Once in total	• Once in total	• Once in total

Reproduction (spring '21 - spring '22, i.e. from March '21 until today)

80. Did you have kittens during this period?

☐ Yes ☐ No ☐ Don't know

81. How many litters did you have during this period?

- State number: ___________ ☐ Don't know

82. How many kittens did the cat(s) have in total during this period?

- State number: ___________ ☐ Don't know

83. How many kittens per litter did you keep?

☐ 0 ☐ 1 ☐ >1 ☐ Don't remember the number

84. How do you handle surplus kittens? (new question)

☐ Gave all of them to new homes
☐ Gave some of them to new homes
☐ Euthanized all of them
☐ Euthanized some of them
☐ Not relevant
☐ Don't remember
☐ Other, describe: ___

New questions

85. Did you reside at the same address in 1998?

☐ Yes ☐ No ☐ Don’t know

86. Do you have the same number of cats as you did in 1998?

- ☐ The same number of cats
- ☐ More cats
- ☐ Fewer cats
- ☐ Don’t know

87. Are your domestic cats marked (microchipped and/or ear marked) and registered in one of the two Danish cat registers? (This question is only asked if the participant has marked that they have domestic cat(s).)

- Yes, all of them are microchipped and/or ear marked as well as registered
- Yes, some of them are microchipped and/or ear marked as well as registered
- No
- Don’t know

88. Are your stable cats marked (microchipped and/or ear marked) and registered in one of the two Danish cat registers? (This question is only asked if the participant has marked that they have stable cat(s).)

- Yes, all of them are microchipped and/or ear marked as well as registered
- Yes, some of them are microchipped and/or ear marked as well as registered
- No
- Don’t know

89. Are you familiar with the legislative change meaning that you can only claim ownership of a cat if it is marked and registered?

☐ Yes ☐ No ☐ Don’t know

90. What is your opinion on neutering or euthanizing cats in the area to keep the population down? (This applies to both your own and your neighbor’s cats as well as to stray cats.)

- ☐ It is okay to euthanize surplus kittens
- ☐ It is not okay to euthanize surplus kittens
- ☐ It is okay to euthanize grown-up cats if there are too many
- ☐ It is not okay to euthanize grown-up cats if there are too many
- ☐ It is okay to euthanize grown-up cats if the alternative is that they are suffering
- ☐ It is not okay to euthanize grown-up cats, even if the alternative is that they are suffering
- ☐ It is best to take precautionary measures by neutering or sterilizing the cats
- ☐ In doubt
- ☐ Don’t know

91. Comment box
