# Supplementary material for: Changes in management of owned cats in the countryside – A comparison of results from surveys undertaken in the same rural area of Denmark in 1998 and 2022
Source: PLoS One. 2025 Feb 19;20(2):e0316704. doi: 10.1371/journal.pone.0316704 (PMC11838870; doi:10.1371/journal.pone.0316704)
Supplement: S4 File — (PDF) [file pone.0316704.s004.pdf]

Samtykke til deltagelse i ”Projekt Landkat vol 2”

”Projekt Landkat vol 2” bygger videre på forskningsprojekt gennemført i 1998. Her blev antallet af katte og måden, de blev hold på undersøgt i et landområde på Vestsjælland. Undersøgelsen foregik på grundlag af en liste af spørgsmål, som blev stillet til beboerne i området. Vi vil nu gentage undersøgelsen og dermed finde ud af, hvad der i mellemtiden er sket i forhold til antallet af katte på landet og i forhold til, hvordan de lever.

Projektet gennemføres af de to dyrlægestuderende Bryndis Wöhler og Ulrike Gade som et specialeprojekt. Vejledere på projektet er professorerne Peter Sandøe, Søren Saxmose Nielsen og Henrik Meilby. Det er planen, at projektets resultater efter de to studerendes specialeeksamen skal danne grundlag for en videnskabelig artikel og efterfølgende for en række yderligere faglige og formidlende artikler.

Du kan læse mere om projektet her: bit.ly/3Kzhb6u

Din besvarelse af spørgeskemaet vil blive behandlet fortroligt. Kun de to studerende og deres vejledere vil have adgang til dem. Når vi er færdige med undersøgelsen, senest ved udgangen af september 2022, vil alle data blive anonymiseret. Det betyder, at det ikke vil være muligt at finde frem til, hvem der svarede hvad.

Du har til enhver tid, indtil data er blevet anonymiseret, mulighed for at trække din besvarelse tilbage. Du kan også vælge at stoppe besvarelsen undervejs.

Efter at have læst ovenstående siger jeg ja til at medvirke som interviewperson i “Projekt Landkat vol2”.

Navn: ____

Adresse: ____

Underskrift:____
