# Supplementary figures and images for: Changes in management of owned cats in the countryside – A comparison of results from surveys undertaken in the same rural area of Denmark in 1998 and 2022
Source: PLoS One. 2025 Feb 19;20(2):e0316704. doi: 10.1371/journal.pone.0316704 (PMC11838870; doi:10.1371/journal.pone.0316704)

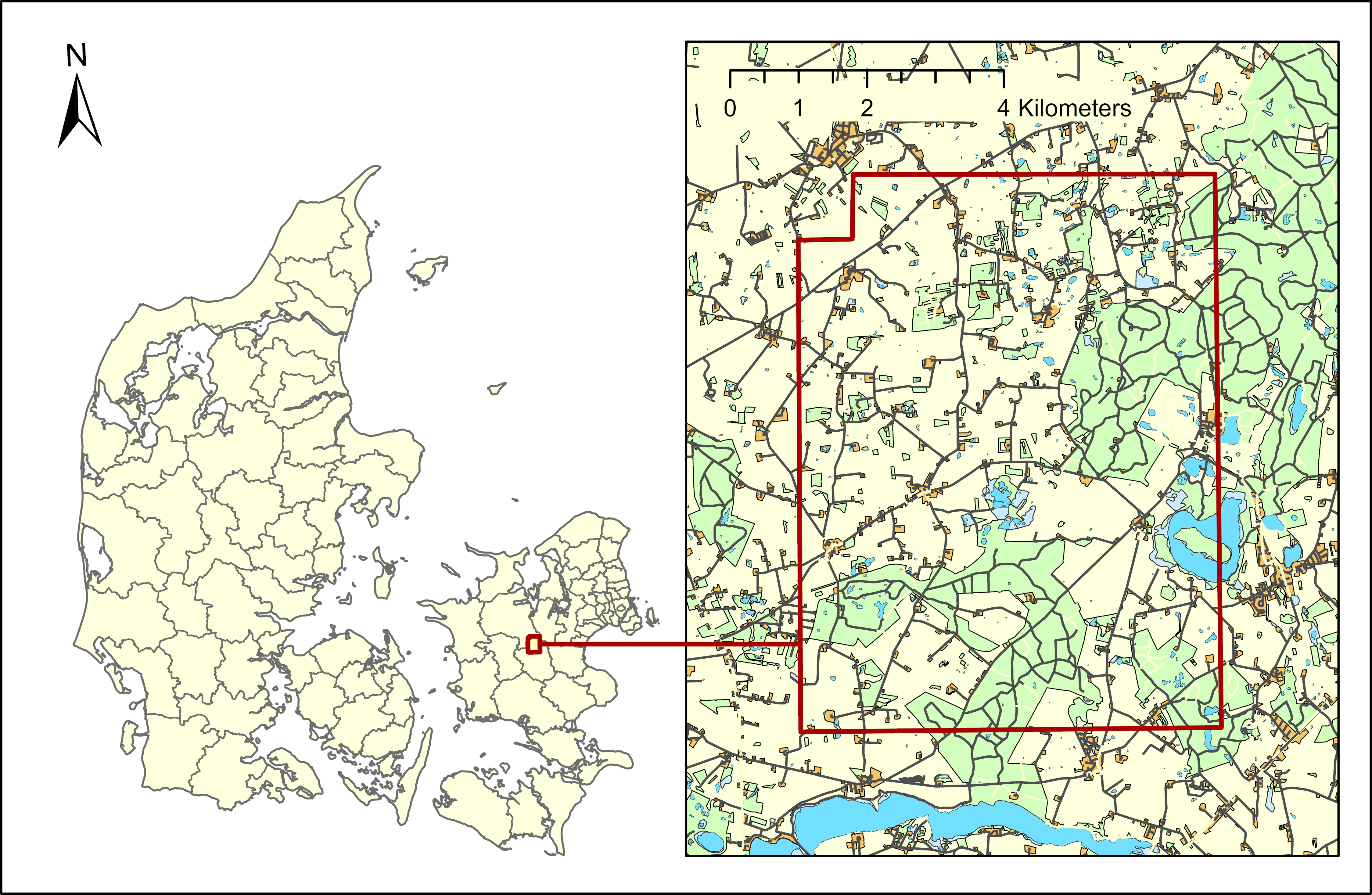

Supplement: S1 Fig — Based on map data provided by The Danish Agency for Climate Data, GeoDanmark, which has granted users permission to use the data under the CC BY 4.0 license. (TIF) [file pone.0316704.s006.tif]
