# Supplementary material for: Changes in management of owned cats in the countryside – A comparison of results from surveys undertaken in the same rural area of Denmark in 1998 and 2022
Source: PLoS One. 2025 Feb 19;20(2):e0316704. doi: 10.1371/journal.pone.0316704 (PMC11838870; doi:10.1371/journal.pone.0316704)
Supplement: Attachment — Submitted filename: PONE-D-24-41843_reviewer_CDR.pdf [file pone.0316704.s007.pdf]

PLOS ONE

Change in management of cats in the countryside – A comparison of results from surveys undertaken in the same rural area of Denmark in 1998 and 2022

--Manuscript Draft--

**Manuscript Number:**	PONE-D-24-41843
**Article Type:**	Research Article
**Full Title:**	Change in management of cats in the countryside – A comparison of results from surveys undertaken in the same rural area of Denmark in 1998 and 2022
**Short Title:**	Change in management of cats in the countryside
**Corresponding Author:**	P. Sandøe, DPhil<br>University of Copenhagen: Kobenhavns Universitet<br>Frederiksberg C, Copenhagen DENMARK
**Keywords:**	Felis catus; country cats; neuter; spay; marking
**Abstract:**	This study investigates changes in the management of owned cats in a rural area of Denmark, over a 24 year time period. Households in a 47 km2 area, and on 23 farms near this area, were presented with the same questionnaire in 1998 and in 2022. Additional data about the number of cats marked and registered in 1998 were provided by two cat registers. The study population was compared to other rural areas in Denmark using data collected in 2021 from a nationwide probability survey of cat owners. A slight drop in number of owned cats and a major shift away from cats living on full-scale farms (under a third in 2022 compared to 1998) and on hobby farms (the number halved in the period) was found, together with nearly a doubling of the number of cats that live in residential homes. In line with this, there was also a significant drop in the proportion of cats without indoor access. Cat owners increasingly adopted measures to manage their cats, including much higher proportions of cats spayed and castrated (e.g., increase from 61% to 98% among male cats with indoor access and from 13% to 70% among male cats without indoor access). There was, also, a major increase in cats that have been marked and registered from 8% to 64%. This change in management has led to many fewer kittens being born and being euthanized. The study area was found to represent rural areas in Denmark well. The findings therefore provide evidence of changing rural human-cat relationships, with rural cats increasingly being managed in the same way as urban owners of companion cats commonly do. The findings also help to explain why the number of unowned cats in Denmark recently has been found to be much lower than was hitherto assumed.
**Order of Authors:**	P. Sandøe, DPhil
	Ulrike Gade, BA
	Marianne Lund Ujvári, MSc
	Bryndis Wöhler, DVM
	Thomas Bøker Lund
	Henrik Meilby
	Clare Palmer
	Søren Saxmose Nielsen
**Additional Information:**	
**Question**	**Response**
**Financial Disclosure**<br><br>Enter a financial disclosure statement that describes the sources of funding for the work included in this submission. Review the submission guidelines for detailed	Yes

requirements. View published research articles from *PLOS ONE* for specific examples.<br><br>This statement is required for submission and **will appear in the published article** if the submission is accepted. Please make sure it is accurate.<br><br>**Funded studies**<br>Enter a statement with the following details:<br>• Initials of the authors who received each award<br>• Grant numbers awarded to each author<br>• The full name of each funder<br>• URL of each funder website<br>• Did the sponsors or funders play any role in the study design, data collection and analysis, decision to publish, or preparation of the manuscript?<br><br>Did you receive funding for this work?	
Please add funding details.<br>as follow-up to "**Financial Disclosure**<br><br>Enter a financial disclosure statement that describes the sources of funding for the work included in this submission. Review the submission guidelines for detailed requirements. View published research articles from *PLOS ONE* for specific examples.<br><br>This statement is required for submission and **will appear in the published article** if the submission is accepted. Please make sure it is accurate.	We received funding from Skibsreder Per Henriksen, R. og Hustrus Fond and Kitty og Viggo Freisleben Jensens Fond for the 2022 study and from Animal Welfare Denmark and Aage V. Jensens Fonde for the 1998 study. The funders had no influence on the design of the study and the interpretation of the presented results.

**Funded studies**<br>Enter a statement with the following details:<br>• Initials of the authors who received each award<br>• Grant numbers awarded to each author<br>• The full name of each funder<br>• URL of each funder website<br>• Did the sponsors or funders play any role in the study design, data collection and analysis, decision to publish, or preparation of the manuscript?<br><br>Did you receive funding for this work?"	
Please select the country of your main research funder (please select carefully as in some cases this is used in fee calculation).<br>as follow-up to "**Financial Disclosure**<br><br>Enter a financial disclosure statement that describes the sources of funding for the work included in this submission. Review the submission guidelines for detailed requirements. View published research articles from *PLOS ONE* for specific examples.<br><br>This statement is required for submission and **will appear in the published article** if the submission is accepted. Please make sure it is accurate.<br><br>**Funded studies**<br>Enter a statement with the following details:<br>• Initials of the authors who received each award<br>• Grant numbers awarded to each author<br>• The full name of each funder<br>• URL of each funder website<br>• Did the sponsors or funders play any role in the study design, data collection and analysis, decision to publish, or preparation of the manuscript?<br><br>Did you receive funding for this work?"	DENMARK - DK
**Competing Interests**	Marianne Lund Ujvári worked for Animal Protection Denmark while doing the first part

Use the instructions below to enter a competing interest statement for this submission. On behalf of all authors, disclose any competing interests that could be perceived to bias this work—acknowledging all financial support and any other relevant financial or non-financial competing interests.<br><br>This statement is required for submission and **will appear in the published article** if the submission is accepted. Please make sure it is accurate and that any funding sources listed in your Funding Information later in the submission form are also declared in your Financial Disclosure statement.<br><br>View published research articles from *PLOS ONE* for specific examples.<br><br>**NO authors have competing interests**<br><br>Enter: *The authors have declared that no competing interests exist.*<br><br>**Authors with competing interests**<br><br>Enter competing interest details beginning with this statement:<br><br>*I have read the journal's policy and the authors of this manuscript have the following competing interests: [insert competing interests here]*<br><br>* typeset	of the project in the late 1990ies. The remaining authors have declared that no competing interests exist.
**Ethics Statement**<br><br>Enter an ethics statement for this submission. This statement is required if the study involved:<br><br>• Human participants<br>• Human specimens or tissue<br>• Vertebrate animals or cephalopods<br>• Vertebrate embryos or tissues	At the time when the first survey was conducted in 1998 there were no ethical guidelines for involving humans subjects in surveys. The primary 2022 survey was approved by The Research Ethics Committee at the Faculties of Science and of Health and Medical Sciences at the University of Copenhagen (ReF: 504-0319/22-5000). Informed written consent was obtained from all interviewees. The consent form is included in the The representative questionnaire study was approved by the Research Ethics Committee at the Faculties of Science and of Health and Medical Sciences at the University of Copenhagen (permission no.: 504/0159/ 20-5000). Respondents did not give informed consent before participation. This approach is employed by Statistics Denmark in all the questionnaire studies that they conduct.

• Field research<br><br>Write "N/A" if the submission does not require an ethics statement.<br><br>General guidance is provided below. Consult the submission guidelines for detailed instructions. **Make sure that all information entered here is included in the Methods section of the manuscript.**	

Powered by Editorial Manager® and ProduXion Manager® from Aries Systems Corporation

**Format for specific study types**<br><br>**Human Subject Research (involving human participants and/or tissue)**<br>• Give the name of the institutional review board or ethics committee that approved the study<br>• Include the approval number and/or a statement indicating approval of this research<br>• Indicate the form of consent obtained (written/oral) or the reason that consent was not obtained (e.g. the data were analyzed anonymously)<br><br>**Animal Research (involving vertebrate animals, embryos or tissues)**<br>• Provide the name of the Institutional Animal Care and Use Committee (IACUC) or other relevant ethics board that reviewed the study protocol, and indicate whether they approved this research or granted a formal waiver of ethical approval<br>• Include an approval number if one was obtained<br>• If the study involved *non-human primates*, add *additional details* about animal welfare and steps taken to ameliorate suffering<br>• If anesthesia, euthanasia, or any kind of animal sacrifice is part of the study, include briefly which substances and/or methods were applied<br><br>**Field Research**<br><br>Include the following details if this study involves the collection of plant, animal, or other materials from a natural setting:<br>• Field permit number<br>• Name of the institution or relevant body that granted permission	
**Data Availability**<br><br>Authors are required to make all data underlying the findings described fully available, without restriction, and from the time of publication. PLOS allows rare exceptions to address legal and ethical concerns. See the PLOS Data Policy and FAQ for detailed information.	Yes - all data are fully available without restriction

Powered by Editorial Manager® and ProduXion Manager® from Aries Systems Corporation

A Data Availability Statement describing where the data can be found is required at submission. Your answers to this question constitute the Data Availability Statement and **will be published in the article**, if accepted.<br><br>**Important:** Stating 'data available on request from the author' is not sufficient. If your data are only available upon request, select 'No' for the first question and explain your exceptional situation in the text box.<br><br>Do the authors confirm that all data underlying the findings described in their manuscript are fully available without restriction?	
**Describe where the data may be found in full sentences. If you are copying our sample text, replace any instances of XXX with the appropriate details.**<br><br>• If the data are **held or will be held in a public repository**, include URLs, accession numbers or DOIs. If this information will only be available after acceptance, indicate this by ticking the box below. For example: *All XXX files are available from the XXX database (accession number(s) XXX, XXX.*).<br>• If the data are all contained **within the manuscript and/or Supporting Information files**, enter the following: *All relevant data are within the manuscript and its Supporting Information files.*<br>• If neither of these applies but you are able to provide **details of access elsewhere**, with or without limitations, please do so. For example:<br><br>*Data cannot be shared publicly because of [XXX]. Data are available from the XXX Institutional Data Access / Ethics Committee (contact via XXX) for researchers who meet the criteria for access to confidential data.*<br><br>*The data underlying the results presented in the study are available from (include the name of the third party*	All relevant data are within the manuscript and its Supporting Information files.

and contact information or URL).<br>• This text is appropriate if the data are owned by a third party and authors do not have permission to share the data.<br><br>* typeset	
Additional data availability information:	

Manuscript
Click here to access/download;Manuscript;Country cats in DK 1998 - 2022 - vers 12 - final ms.docx

Change in management of cats in the countryside – A comparison of results from surveys undertaken in the same rural area of Denmark in 1998 and 2022

Peter Sandøe[1,2*], Ulrike Gade[1], Marianne Lund Ujvári[3], Bryndis Wöhler[1], Thomas Bøker Lund[2], Henrik Meilby[2], Clare Palmer[4], Søren Saxmose Nielsen[1]

[1] Department of Veterinary and Animal Sciences, University of Copenhagen, Denmark.

[2] Department of Food and Resource Economics, University of Copenhagen, Denmark.

[3] Animal Protection Denmark

[4] Department of Philosophy, Texas A&M University, USA.

* Corresponding author

E-mail: pes@sund.ku.dk

Abstract

This study investigates changes in the management of owned cats in a rural area of Denmark, over a 24 year time period. Households in a 47 km^2 area, and on 23 farms near this area, were presented with the same questionnaire in 1998 and in 2022. Additional data about the number of cats marked and registered in 1998 were provided by two cat registers. The study population was compared to other rural areas in Denmark using data collected in 2021 from a nationwide probability survey of cat owners. A slight drop in number of owned cats and a

major shift away from cats living on full-scale farms (under a third in 2022 compared to 1998) and on hobby farms (the number halved in the period) was found, together with nearly a doubling of the number of cats that live in residential homes. In line with this, there was also a significant drop in the proportion of cats without indoor access. Cat owners increasingly adopted measures to manage their cats, including much higher proportions of cats spayed and castrated (e.g., increase from 61% to 98% among male cats with indoor access and from 13% to 70% among male cats without indoor access). There was, also, a major increase in cats that have been marked and registered from 8% to 64%. This change in management has led to many fewer kittens being born and being euthanized. The study area was found to represent rural areas in Denmark well. The findings therefore provide evidence of changing rural human-cat relationships, with rural cats increasingly being managed in the same way as urban owners of companion cats commonly do. The findings also help to explain why the number of unowned cats in Denmark recently has been found to be much lower than was hitherto assumed.

Keywords: *Felis catus*, country cats, neuter, spay, marking

Introduction

This paper presents a new study that investigates changes in the management and care of owned cats in a rural area of Western Zealand, Denmark, over a 24 year time period. It demonstrates that rural cat owners have increasingly adopted measures to manage their cats, including ~~by~~ spaying, neutering, and microchipping, and that increasing numbers of the cats now have access into their owner' homes. This change in management has led to fewer kittens being born, and fewer kittens and cats being euthanized. We also show that the study

area represents rural areas in Denmark well. As such, the findings are of significance by providing evidence of changing rural human-cat relationships, with cats increasingly being managed in the same way as is common among urban owners of companion cats. The findings also help to explain recent results from another study that the number of unowned, stray, and feral domestic cats in Denmark is much lower than hitherto assumed.

Populations of unowned domestic cats – whether unsocialized feral cats that have never lived with humans, or socialized cats that have strayed or been abandoned – are regarded as problematic in many countries (1–3). They are argued to be a nuisance to people, to carry disease to humans, owned cats and wildlife (4–6); to suffer from poor welfare (4,7,8) and to reduce biodiversity by hunting (9). For all these reasons, there's wide agreement that populations of unowned cats should be controlled. Strategies for doing so include rehoming socialized cats through animal shelters, trapping and neutering unsocialized cats and returning them to their place of origin (5,6,10), and trapping and euthanizing them (7). Because domestic cats are adored by some and vilified by others, these strategies are all controversial: they may be viewed as going too far, especially in killing cats; or not far enough, for instance by returning feral cats to roam at their place of origin, once sterilized.

However these population control strategies are regarded, they will all be ineffective if there is an ongoing influx of domestic cats from the owned population into the unowned population through both straying and reproduction; and studies consistently show that cat colonies increase by immigration (10,11). This means that to prevent unowned cat populations from growing, measures are needed not only to control existing populations, but also to prevent movement from owned to unowned populations.

One way of reducing this movement is by keeping cats indoors. In some countries, a significant percentage of cats do not have outdoor access. For instance 63% of cats in cat-only households have been found to live wholly indoors in the USA (12). However, this practice is controversial, generating significant concerns about cat welfare (13–15). And confining cats indoors isn't widely accepted outside North America. For instance, a 2010 study found that 95% of cats in New Zealand were allowed outdoor access (16); and across Europe, most cats seem to have indoor/outdoor lifestyles (17). In Denmark, the proportion of cats confined indoors or with only restricted outdoor access eg. to fenced areas that keep cats enclosed, is around 25% (18), for example. So, confining cats is unlikely to succeed if taken as the main way of preventing the flow of owned cats and their offspring into unowned populations.

Another way of staunching the flow is to embrace what's sometimes called "responsible pet ownership". In the case of cats, this involves spaying and neutering to prevent the production of offspring. It also involves marking and registering cats, by the insertion of microchips or other identifying marks and the creation of a registry of marked cats to facilitate the return of strays. Some states and countries are even beginning to require either sterilization, microchipping, or both. (Microchipping, for instance, has recently become a legal requirement in the UK (19), while the state of Western Australia now requires both sterilization and microchipping (20)).

However, historically at least, voluntary uptake of these measures seems to have varied significantly across different demographics – especially between urban/suburban and rural

communities. In particular, early studies from the USA found a low level of spaying and neutering of farm cats and other cats in rural areas (21–23). This matters because even if reproduction is controlled in urban areas, high reproductive rates among rural cats leads to dispersal (24) across rural areas and also likely inflow to urban/suburban areas, especially where there's provision of supplemental food (25).

Recent studies from Italy, the United Kingdom and USA however, seem to suggest that the gap between rural and urban cat management is diminishing, with higher rural uptake in terms of spaying and neutering (26–28). If the results of these studies can be generalized to other areas and other countries, this would suggest that populations of unowned cats should be diminishing as rural cat owners adopt measures that control the growth in cat populations. Interestingly, a recent study in Denmark found just such a drop in the number of unowned cats: the number was about 80% lower than hitherto assumed and expected, based on measures of feed availability as the only factor restricting the stray and feral cat population (29). A similar density of unowned cats was found in a near-simultaneous study undertaken in the UK (30). These findings directly prompted the research presented in this paper. We wanted to investigate whether a reduction in unowned cat populations could be explained by changes in the ways rural communities, in Denmark at least, have managed and cared for their cats over time.

The best way to test this hypothesis would be to study changes in cat management in an identified rural area over time. However, as far as we could find no such studies exist. This led us to the current study where we began with the findings of a previous questionnaire study on rural cat management undertaken in 1998 (31). The study involved households in a

47 km^2 countryside area in Western Zealand, Denmark, and on 23 additional nearby farms. We then carried out a follow-up study involving the same households and farms 24 years later, in 2022. The 1998 study was only published as a report in Danish; this is the first presentation of its results to an international scholarly audience. The 1998 study did not contain information about how many cats were marked and registered at that time; so for this information, we drew on two official existing national cat registers that were both active in 1998. In addition, the study population was compared to the target population of rural areas in Denmark using a probability-based survey of cat owners where data was collected in 2021.

The current study, then, is the first of its kind. It documents changes in management and care of owned cats (*Felis catus*) over a period of 24 years in a typical rural area. Our research questions were: 1) How has the number of owned cats and their distribution between farms and residential houses changed? 2) How has management in terms of cats with indoor access vs. cats with outdoor access only (barn cats) changed? 3) How has kitten production in the area changed? 4) How have use of methods for reproductive control and other means of cat population control changed? 5) How has the uptake of methods of marking and registering changed? 6) How representative is the studied area for rural Denmark?

Materials and Methods

Survey studies in 1998 and 2022 – overview

This cross-sectional study was conducted as a follow-up to a cross-sectional study carried out in 1998 (31). The study area was identified based on the 47 km^2 zone in the middle of the largest Danish island, Zealand (Figure S1 in Supplementary materials), and an additional 23 active agricultural premises with production animals located a maximum of 14 kms from the

47 km^2 zone, as used in the original study. The original data from the 1998-study do not exist, and data were therefore extracted from the study report (31), included in the data repository. Data collection in the present study occurred from 5 May to 1 July 2022. A questionnaire was developed to replicate the data collection in 1998. However, some adaptations had to be made owing to some development in the use of the properties for other purposes than farming and housing. The original study questionnaire was administerd in person, and this was copied in the new study: Person MU walked from house to house in 1998, and persons UG and BW did the same in 2022.

Survey studies in 1998 and 2022 – respondents

In 1998, the study area included 488 addresses in the 47 km^2-zone and 23 farms, i.e. 511 properties were visited. In 2022, all addresses were extracted from the same area via the Danish Address Registry (Danmarks Adresser, The Danish Agency for Data Supply and Infrastructure, Copenhagen NV, Denmark) on 28 March 2022. An information letter was sent to all addresses; some of these were returned with information about the reason. All returned letters and non-responders were investigated via Google Maps (Google Inc., Mountainview, California, USA), Krak (Krak Danmark, Copenhagen V, Denmark), and the Danish Central Herd Registry (Danish Veterinary and Food Administration, Glostrup, Denmark), to see whether the premises contained facilities suitable for human housing, whether production animals were recorded on the premises now, and whether production animals had been present earlier. Addresses with no facilities suitable for human housing (e.g. a church chapel, a power transformation station, abandoned houses in decay, and premises with commercial workshops only) were excluded. Premises with multiple addresses, e.g. apartment-type buildings or other houses with multiple separate households were included but kept separate. Addresses where workshops and households were combined were included. All included

addresses were then defined as a household, except for businesses that potentially could host cats (e.g. hotels, bed and breakfasts, golf courses etc.), which were categorized as "other"; addresses. Existing farms were categorised as such, and former farms were categorised as "small-scale or hobby farms". The additional 23 addresses with farms that were included in 1998 were also included in 2022. They were re-categorised to their new use if they were no longer farms. Two people (UG and BW) assisted by a hired student assistant went from house to house, introduced the project and asked if the owner would like to participate. Written informed consent was obtained from all participants in the 2022 study.

Survey studies in 1998 and 2022 – Questionnaire

An electronic questionnaire was developed for the 2022 study in SurveyXact (Rambøll Management Consulting, Aarhus N, Denmark), based on the questions asked by Ujvári (1999), with some additional questions, including questions about whether cats were marked and registered. The 1998-questionnaire is available in Ujvári (1999) to be found in the data repository together with the 2022-questionnaire (also translated into English). Briefly, the following information was retrieved at each address from the respondents: type of property, number of cats by type (cat with indoor access or cat with outdoor access only) and by sex (male, female, unknown), the purpose of keeping cats (companion animal, predator, other) by cat-type, if population control was used, and if yes: how (hormonal treatment, castration, sterilisation, killing of adults or kittens), production of kittens in the past year, along with sourcing of the kittens produced (killing, keeping, sold/given away), and registration of the cats in official registries.

Survey studies in 1998 and 2022 – data analysis

Because the raw data from the 1998-survey no longer existed (they were accidentally lost before the current collaboration started), and some data were presented as graphs only, some of the 1998 data were extracted by a combination of measuring the percentages shown as height of bars in barplots with a ruler, combining this with the total number of cats contributing to the graphs, and using this to estimate the number of cats for each bar.

The proportion of responders overall was given for the 1998-survey, while it was calculated overall and by property-type for the 2022-survey.

For the 2022-survey, the number of cats per cat type (outdoor only vs. with indoor access) and property type was estimated. For both surveys, the number of cats overall (1998 and 2022) and by property type (2022 only) was also estimated. Since neither the 1998 nor the 2022 data reached a 100% response percentage, these estimations were adjusted to take non-responders into account. This was done by dividing the number of cats by the proportion of responders. Furthermore, the number of kittens produced was estimated.

The proportions in 1998 were compared to proportions in 2002 using the prop.test-function in R (R Core Team (2022). A P-value of 0.05 was considered statistically significant.

Data from the two Danish cat registers

Two national cat registers exist in Denmark: Dansk Katteregister (c/o Inges Kattehjem, Glostrup, Denmark) and Det Danske Katteregister (c/o Danish Veterinary Association, Frederiksberg, Denmark), and both were requested to provide data in the postal codes

covering the study area (4100, 4174, 4330, 4360, and 4370) in 1998. Subsequently, the addresses that were provided with registered cats were merged with the addresses in the study area to determine the proportion of registered cats in the study area in 1998.

National survey conducted in 2021 – overview

This was a cross-sectional questionnaire survey administered and carried out by Statistics Denmark (Copenhagen Ø, Denmark). A probability sampling procedure was employed involving a random selection of Danes. In total, 5,027 persons in the age interval 18-89 years were randomly drawn from the Danish Civil Registration System and invited to participate in the survey via e-mail that had a link to an online questionnaire. The invitees that did not respond following emailed invitations were contacted by telephone, or letter, and encouraged to participate. At that point, it was also possible for invitees to respond to the questionnaire via an interviewer-assisted telephone interview. Data collection was carried out in May-June 2021.

National survey conducted in 2021 – Questionnaire and register data information

The questionnaire aimed to investigate the distribution of companion animals in Denmark, the managing of companion animals, and owner-companion animal attachment. Here only data on how people manage their cats (355 respondents) was used, based on two questions about 1) whether the cat has indoor access, and 2) whether the cat is marked (with a collar or a chip) and/or registered. For those respondents that had more than one cat, they were not asked to provide management information about all cats. Instead, they were asked to order the cats' names alphabetically and give information for the first cat only.

We further used register data information provided by Statistics Denmark about the population density of the geographical area that the cat owning participants, hence also the cats, live in. Specifically, a population density variable with three levels was constructed (1: Large city area with (>99,999 inhabitants); 2: Medium-small city area (>199 & <100,000 inhabitants); 3: Rural area or village (<200 inhabitants).

National survey conducted in 2021 – Data analysis

The percentage of cats that were only outdoor cats, and the percentage of marked/registered cats, were reported for each population density level.

To ensure that the results are representative of Danish households, weighted percentages were reported using a weight variable that adjusts the sample so that it matches the background population comprising approximately 3.1 million families in Denmark. Statistics Denmark constructed this weight variable based on the following family-level census information: population density; family income; number of family members; region of Denmark; housing type; dwelling size; and family type (i.e. ‘single without children’, ‘single with children’, ‘couple with children’, and ‘couple without children’).

We compared whether there were differences across the population density levels using a χ^2-test and considered a P-value of 0.05 to be statistically significant. The weight variable was not activated when carrying out these inferential statistics.

To test whether the percentage of cats with outdoor access and that were marked/registered in the study area was different from other rural areas of Denmark one sample binomial tests were carried out.

Results

Comparison 1998 and 2022 – respondents

A total of 488 addresses within the study area and 23 addresses outside the study area were visited in 1998. Of these 511, 467 (91.4%) responded with full information; on two properties, only partial information was retrieved, and they were therefore excluded. For the 2022-survey, the sample size and proportion responding (stratum-specific) were: 26 (57.7%) for full-scale farms, 151 (60.9%) for small-scale/hobby farms, 273 (60.4%) for houses (incl. those with multiple households), and 24 (54.1%) for Other. Overall, this corresponded to 474 possible respondents where responses were received from 285 (60.1%).

Comparison 1998 and 2022 – number of cats and ways of keeping them

In 1998, 59.6% of the households had cats, in comparison to 53.0% of the households in 2022. Overall, 840 cats were estimated to be present in the area in 1998, and 764 cats in 2022. The number and mean number of cats by property type are shown in Table 1.

Table 1. Distribution of cats for different property types, with observed and estimated number of cats. The estimated number of cats are observed number of cats divided by the percentage of respondents.

Property type	Observed data			Estimated cat population	
	No. of properties[§]	No. of cats	Mean no. cats	% respondents	No. of cats

	1998	2022	1998	2022	1998	2022	1998*	2022	1998	2022
Household in residential home#	258	266	246	309	0.95	1.16	91.4	60.4	269	511
Farm, small-scale (hobby)	130	50	216	71	1.66	1.42	91.4	60.9	236	117
Farm, full-scale	79	28	306	55	3.87	1.96	91.4	57.7	335	95
Other	0	20	0	22	0	1.10	91.4	54.1	0	41
Total	467	364	768	457	1.64	1.26	91.4	60.1	840	764

§) Including properties with no cats and additional properties outside the area

#) Including properties where single houses are divided in apartment type households

*) For 1998, only average % respondents existed, and this was therefore used for all property types

In 1998 cats lived on 269 of the properties that responded. Of these properties 117 (43%) had cats with no indoor access and 181 (67%) had cats with indoor access (note that the sum is higher than 100% because some properties had both cats with no indoor access and cats with indoor access). In 2022 there were cats on 193 of the properties that responded. Of these properties 59 (31%) had cats with no indoor access and 148 (77%) had cats with indoor access. This was a significant decrease in the proportion of cats with no indoor access from 1998 to 2022 (p=0.006).

Comparison 1998 and 2022 – Kitten production and management of cats

The production of kittens is summarised in Table 2. Overall, 410 kittens were estimated to have been produced in 1998 compared with 134 in 2022, i.e. a reduction to approximately one third of the previous number, and also the number of surplus kittens (i.e. kittens that were not adopted or euthanized) was reduced from 189 to 58, i.e. 31% of surplus kittens produced in the past. Almost half (66/134) of kittens produced were from small-scale /hobby farms in 2022.

Table 2. Estimated production of kittens by year and type of household. Only overall numbers were available for 1998.

Type of household	Kittens born		Kittens kept	Euthanised kittens	Surplus kittens	
	1998	2022	2022	2022	1998	2022
Household in villa[#]		35	18	9		8
Farm, small-scale (hobby)		66	21	16		29
Farm, full-scale		20	10	2		8
Other		13	0	0		13
Total	410	134	49	27	189	58

[#]) Including properties where single houses are divided in apartment type households

The use of different types of reproductive control used in each year for the two types of cats are summarised in Table 3 with major changes seen for all types of control. Use of birth

control hormones and killing of adult cats and kittens have mostly stopped, whereas sterilizing of both male and female cats have increased substantially for both types of cats.

Table 3. Types of regulation of the cat populations in 1998 and 2022, stratified by cat type.

		1998			2022			P-value*
Cat type	Category	N*	n	%	N	n	%	
Access indoor	Birth control hormones	104	40	38	93	4	4	<0.0001
	Sterilised female cat	104	46	44	93	86	92	<0.0001
	Castrated male cat	121	74	61	85	83	98	<0.0001
	Killing adults	104	5	5	137	1	0.7	0.11
	Killing kittens	104	22	21	137	2	1	<0.0001
Outdoor only	Birth control hormones	83	26	31	43	3	7	0.004
	Sterilised female cat	83	16	19	43	27	63	<0.0001
	Castrated male cat	89	12	13	37	26	70	<0.0001
	Killing adults	83	13	16	59	3	5	0.09
	Killing kittens	83	43	51	59	5	8	<0.0001

*) N is the total number of responders with the specific type of cats in the stratum, and n is the number of responders practicing the specific type of regulation. N is not the same for the different types of control measures, as not all responders had both male and female cats. The

P-value indicates whether there is a difference between the practice in 1998 and 2022 based on the Z-test.

Comparison 1998 and 2022 – number of cats marked and registered

By comparing information from the two Danish cat registers about addresses with postcodes overlapping with the area studied and the actual addresses in the area, it was found that 41 out of 511 addresses in 1998 had a cat that was marked and registered, a proportion of 8%.

In the 2022 survey in total 123 (74%) of those 167 respondents with cats who responded to the question (6 respondents answered "don't know") said all or some of their cats were marked or registered. On properties where cats had indoor access 78% (95/122) answered that cats were marked or registered; for those with cats with no indoor access it was 59% (35/59) (note that a few properties had both cats with indoor access and cats with no indoor access).

Comparison of findings from study area 2022 with national survey from 2021

In total, 2,347 of the 5,027 invited respondents completed the 2021 national survey (response percentage 47%). Of these, 355 reported that there was at least one cat in the family. Eight respondents were removed due to missing register data information about population density giving a sample size of 348. This sample, however, was also reduced slightly on a case-by-case basis in the tables below, because respondents that answered "don't know" were removed.

In Table 4 the share of outdoor only cats in the study area is compared to differently populated areas according to the national survey to see whether the study area is like other rural areas in Denmark in this regard. As expected, outdoor cats were much more frequent in rural areas compared to more densely populated areas in Denmark, (χ^2 test: $p<0.001$). The share of outdoor cats in the study area (31%) is very similar to other rural areas in Denmark (32%), and the difference is not statistically significant (one sample binomial test: $p=0.37$).

Table 4. Percentage of outdoor only cats stratified by population density from the 2021 national survey (unweighted n=344*) and compared with the study area

		%**
National survey	Large city area (>99,999 inhabitants)	1
	Medium-small city area (>200 & <100,000 inhabitants)	5
	Rural area or village (<200 inhabitants)	32
	Total	12
Study area		31

Weighted percentages are reported in the national survey.

* The sample size was reduced from 348 to 344 because four respondents that answered “don’t know” were removed from the analysis.

** The question formulation in the national survey was: “Is the cat able to go outdoors?”. The relevant response option was “Yes, and the cat is seldom or never indoors”.

Finally, in Table 5 we compare the relative number of marked and registered cats in differently populated areas according to the national survey with the share of these cats in the study area. The share of marked and/or registered is lower in rural areas compared to more densely populated areas in Denmark (χ^2 test: $p<0.001$). In line with this, the share of marked/registered cats in the study area (64%) is lower than the shares observed in medium/small city areas (87%; one sample binomial test: $p<0.001$) and large city areas (88%; one sample binomial test: $p<0.001$). On the other hand, the share of marked/registered cats in the study area is higher than in other rural areas in Denmark (51%; one sample binomial test: $p<0.001$).

Table 5. Percentage of marked and/or registered cats stratified by population density from the 2021 national survey (unweighted n=327*) and compared with the study area

		%**
National survey	Large city area (>99,999 inhabitants)	88
	Medium-small city area (>200 & <100,000 inhabitants)	87
	Rural area or village (<200 inhabitants)	51
	Total	77

Study area		64

Weighted percentages are reported in the national survey.

* The sample size was reduced from 348 to 327 because 21 respondents that answered “don’t know” were removed from the analysis.

** The question formulation in the national survey was: “Is the cat marked?”. The following response option were considered as a marked or registered cat: “Ear marked but not registered”, “Ear marked and registered”, “Chip marked but not registered”, “Chip marked and registered“, “Marked with a collar with contact information”.

Discussion

Outline of main findings

In light of the results presented above it is now possible to answer our research questions:

1) How has the number of owned cats and their distribution between farms and residential houses changed? The study shows there has been a slight drop in the number of owned cats and a major shift away from cats living on full-scale farms (under a third in 2022 compared to 1998) and on hobby farms (the number halved in 2022 compared to 1998). However, nearly double the number of cats live in residential homes. This shift also clearly reflects a drop in the number of full-scale farms in Denmark during the period (cf. Table 1). According to statistics from Statistics Denmark (www.statistikbanken.dk), the number of full-scale farms has dropped from 25.549 in 1998 to 7.598 in 2021.

2) How has management in terms of cats with indoor access vs. cats with no indoor access changed? As can be seen from our data, the number of cats that have no indoor access has gone down relative to the number of cats living as companion cats with indoor access, which has gone up.

3) How has kitten production in the area changed? Both the number of kittens born and the number of suplus kittens has gone down to a third of the orginal number from 1998 to 2022. This could be an important part of the explanation of the apparent drop in the number of unowned cats in Denmark (cf. Table 2).

4) How have use of methods for reproduction control and other means of cat population control changed? Here, there have been important changes regarding the use of birth control hormones, spay and neuter and killing. Use of birth control hormones ("the pill") has gone down dramatically, probably reflecting that veterinary guidelines no longer recommend birth control hormones as a means of contraception in cats (32). On the other hand the use of permanent fertility control measures has gone up dramatically, so that neutering of female cats has gone up from 44% to 92% among those with indoor access and from 19% to 63% for those which are outdoor only (cf. Table 3). And for male cats the castration proportion has gone from 61% to 98% among those with indoor access and from 13% to 70% among those living in barns. Finally, killing as a means of population control among adult cats has gone from 5% to 1% among those with indoor access and from 16% to 5% among cats with no indoor access. For kittens it has gone from 21% to 1% among those with indoor access and from 51% to 8% among those living outdoor only. This is clearly a major shift both towards

more responsible cat ownership and a growing aversion towards killing cats as a means of reproduction control.

5) How has the uptake of methods of marking and registering changed? Again, here there has been a dramatic change. Back in 1998 only 8% of the owned cats in the area studied were marked and registered. In 2022 this had gone up to 64% with more indoor cats (78%) than cats with no indoor access (54%) being marked and registered.

6) How representative is the studied area for rural Denmark? Compared with the survey covering all of Denmark, the area studied seems to fall within the rural segment, with the same level of outdoor only cats compared to the average rural group, but with more cats marked and registered compared to the average rural area (Cf. Table 4 and 5).

Overall, our study shows that over the past 2-3 decades there has been a major shift in how rural cats are managed in Denmark: A higher proportion of cats have indoor access. Gradually, management practices with surgical reproductive control measures and the marking and registering of owned cats is becoming the norm; and traditional barn cats are slowly reducing in numbers. Rural inhabitants are now also much less likely to kill cats. These developments, we speculate, may reflect a change in attitude to cats, where in rural as well as in urban areas they are increasingly viewed as companions or even as family members. This would also explain the relatively small population of stray and feral cats found in the 2022 study (29).

Comparison with previous studies

No other studies have attempted to do what we have done here. All existing studies of management of rural cats in the Global North are cross-sectional studies undertaken at a specific point in time. More responsible cat management practices are, however, found in the most recent studies compared to the earlier studies, which is in line with the findings of the present study.

Limitations

Our use of the early study from 1998 was affected by three limitations. Firstly, the original dataset no longer exists, so that some of the data had to be reconstructed based on inspection of graphs. Secondly, the original questionnaire had some limitations – for example, it was not possible to add up the number of cats across the two main categories, "with indoor access" and "outdoor access only". Thirdly, there were no real measures of people's attachment to their cats and therefore efforts to explain the findings in terms of changes in *attitudes* to owned cats are bound to be speculative.

The later study was limited by the lower response rate of 60% compared to 91% in the early study. However, by normal standards, 60% is still a very high response rate. In the later study it appears that some possible respondents may have been missed due to letters being returned by the postal service, even though there were people living at some of the addresses.

Also it is a limitation that number of cats being marked and registered are measured in different ways in 1998 and 2022, with data from cat registries in 1998 and survey based data in 2022. However, given the large difference in numbers, this limitation is likely not very important.

However, overall the limitations are not seen as major.

Acknowledgements

We want to thank the respondents for their participation in the surveys on which this presentation is based. We also want to thank Jørgen S. Petersen and Per Hvenegaard for information on cat registration in 1998. Also we owe thanks to Eliza Ruiz Izaguirre for assistance in search of literature and to Nicoline Skandov for assisting UG and BW with the data collection in 2022. Finally, thanks are due to Skibsreder Per Henriksen, R. og Hustrus Fond and Kitty og Viggo Freisleben Jensens Fond for economic support of the 2022 study and Animal Welfare Denmark and Aage V. Jensens Fonde for economic support of the 1998 study. The funders had no influence on the design of the study and the interpretation of the presented results.

Ethical permissions

At the time when the first survey was conducted in 1998 there were no ethical guidelines for involving humans subjects in surveys. The primary 2022 survey was approved by The Research Ethics Committee at the Faculties of Science and of Health and Medical Sciences at the University of Copenhagen (ReF: 504-0319/22-5000). Informed written consent was obtained from all interviewees. The consent form is included in the data repository.The representative questionnaire study was approved by the Research Ethics Committee at the Faculties of Science and of Health and Medical Sciences at the University of Copenhagen (permission no.: 504/0159/ 20-5000). Respondents did not give informed consent before

participation. This approach is employed by Statistics Denmark in all the questionnaire studies that they conduct.

References

1. Changrani-Rastogi A, Thakur N. Attitudes towards urban stray cats and managing their population in India: a pilot study. Front Vet Sci. 2023; 10:1274243.

2. De Ruyver C, Abatih E, Villa PD, Peeters EHKA, Clements J, Dufau A, m.fl. Public opinions on seven different stray cat population management scenarios in Flanders, Belgium. Res Vet Sci. 2021; 136:209–19.

3. Legge S, Murphy BP, McGregor H, Woinarski JCZ, Augusteyn J, Ballard G, m.fl. Enumerating a continental-scale threat: How many feral cats are in Australia? Biol Conserv. 2017; 206:293–303.

4. Gunther I, Raz T, Berke O, Klement E. Nuisances and welfare of free-roaming cats in urban settings and their association with cat reproduction. Prev Vet Med. 2015; 119(3–4):203–10.

5. Hiby E, Eckman H, MacFarlaine I. Cat population management. In: Turner DC, Bateson P, eds. The Domestic Cat. 3. ed. Cambridge University Press; 2013, pp. 215–30.

6. Slater MR. Understanding issues and solutions for unowned, free-roaming cat populations. J Am Vet Med Assoc. 2004; 225(9):1350–4.

7. Jessup DA. The welfare of feral cats and wildlife. J Am Vet Med Assoc. 2004; 225(9):1377–83.

8. Slater MR. The welfare of feral cats. In: Rochlitz I, ed.. The welfare of cats. Dordrecht ;: Springer; 2005, pp. 141-175.

9. Lepczyk CA, Fantle-Lepczyk JE, Dunham KD, Bonnaud E, Lindner J, Doherty TS, m.fl. A global synthesis and assessment of free-ranging domestic cat diet. Nat Commun. 12. 2023; 14(1):7809.

10. Levy JK, Crawford PC. Humane strategies for controlling feral cat populations. J Am Vet Med Assoc. 2004; 225(9):1354–60.

11. Zaunbrecher KI, Smith RE. Neutering of feral cats as an alternative to eradication programs. J Am Vet Med Assoc. 1993; 203(3):449–52.

12. Bayer Healthcare LLC. News Release [Internet]. 2013. https://www.prnewswire.com/news-releases/bayer-aafp-study-reveals-half-of-americas-74-million-cats-are-not-receiving-regular-veterinary-care-216415781.html [accessed 20 September 2024].

13. Abbate CE. A Defense of Free-Roaming Cats from a Hedonist Account of Feline Well-being. Acta Anal. 2020; 35(3):439–61.

14. Fischer B. Keep Your Cats Indoors: a Reply to Abbate. Acta Anal. 2020; 35(3):463–8.

15. Palmer C, Sandøe P. For their own good: captive cats and routine confinement. In: Gruen L, ed. Ethics of Captivity. Oxford University Press; 2014. pp. 135–55.

16. Farnworth M, Campbell J, Adams N. Public awareness in New Zealand of animal welfare legislation relating to cats. N Z Vet J. 2010; 58(4):213–7.

17. Foreman-Worsley R, Farnworth MJ. A systematic review of social and environmental factors and their implications for indoor cat welfare. Appl Anim Behav Sci. 2019; 220:104841.

18. Sandøe P, Nørspang AP, Kondrup SV, Bjørnvad CR, Forkman B, Lund TB. Roaming Companion Cats as Potential Causes of Conflict and Controversy: A Representative Questionnaire Study of the Danish Public. Anthrozoös. 2018; 31(4):459–73.

19. Department for Environment, Food & Rural Affairs. Treasured pets now safer as microchipping for cats becomes compulsory [Internet]. 2023 https://www.gov.uk/government/news/treasured-pets-now-safer-as-microchipping-for-cats-becomes-compulsory [accessed 20 September 2024].

20. Government of Western Australia. Laws for responsible cat owners. Information about the Cat Act 2011 [Internet]. 2024 https://www.dlgsc.wa.gov.au/local-government/community/cats-and-dogs/laws-for-responsible-cat-owners [accessed 20 September 2024].

21. Coleman JS, Temple SS. Rural Residents' Free-Ranging Domestic Cats: A Survey. Wildl Soc Bull 1973-2006. 1993; 21(4):381–90.

22. Mahlow JC. Estimation of the proportions of dogs and cats that are surgically sterilized. J Am Vet Med Assoc. 1999; 215(5):640–3.

23. Warner RE. Demography and Movements of Free-Ranging Domestic Cats in Rural Illinois. J Wildl Manag. 1985; 49(2):340.

24. Liberg O. Spacing Patterns in a Population of Rural Free Roaming Domestic Cats. Oikos. 1980; 35(3):336.

25. Liberg O, Sandell M, Pontier D, Natoli E. Spatial organisation and reproductive tactics in the domestic cat and other felids. In: Turner DC, Bateson P, eds. The Domestic Cat: the biology of its behaviour. Second edition. Cambridge: Cambridge University Press; 2000. s. 119–47.

26. Carvelli A, Iacoponi F, Scaramozzino P. A Cross-Sectional Survey to Estimate the Cat Population and Ownership Profiles in a Semirural Area of Central Italy. BioMed Res Int. 2016; 2016:1–9.

27. Lord LK. Attitudes toward and perceptions of free-roaming cats among individuals living in Ohio. J Am Vet Med Assoc. 2008; 232(8):1159–67.

28. Roberts C, Gruffydd-Jones TJ, Clements J, Jones TW, Farnworth MJ, Murray JK. Cats on farms in the United Kingdom: numbers and preventive care. Vet Rec. 2018; 183(1):23–23.

29. Nielsen HB, Jensen HA, Meilby H, Nielsen SS, Sandøe P. Estimating the Population of Unowned Free-Ranging Domestic Cats in Denmark Using a Combination of Questionnaires and GPS Tracking. Animals. 2022; 12(7):920.

30. McDonald JL, Skillings E. Human influences shape the first spatially explicit national estimate of urban unowned cat abundance. Sci Rep. 2021; 11(1):20216.

31. Ujvári M. Projekt “Landkat”. Dyrenes Beskyttelse; 1999, 49 pp.

32. Asa CS. Contraception in Dogs and Cats. Vet Clin North Am Small Anim Pract. 2018; 48(4):733–42.

Supporting information

The following files are available as supporting information:

1) The original report, Ujvari, 1999
2) Survey text from 2022
3) Data set from the 2022 study
4) A map of the area
5) Informed consent form from 2022

Click here to access/download
Supporting Information
Questionnairy 2022 - Country cats.pdf

Click here to access/download
Supporting Information
Consent form 2022 - Country cats.pdf

Click here to access/download
Supporting Information
Ujvari, 1999.pdf

Click here to access/download
Supporting Information
Map Country cats paper.pdf

Click here to access/download
Supporting Information
Data country cats.xlsx
