# Supplementary material for: Changes in management of owned cats in the countryside – A comparison of results from surveys undertaken in the same rural area of Denmark in 1998 and 2022
Source: PLoS One. 2025 Feb 19;20(2):e0316704. doi: 10.1371/journal.pone.0316704 (PMC11838870; doi:10.1371/journal.pone.0316704)
Supplement: Attachment — Submitted filename: Response to editor and reviewers - PONE-D-24-41843-final.docx [file pone.0316704.s008.docx]

PONE-D-24-41843 - Responses to comments from editor and reviewers

| **Nb.** | **Editor comments** | **Responses** |
| --- | --- | --- |
| 1 | 1. Please ensure that your manuscript meets PLOS ONE's style requirements, including those for file naming. The PLOS ONE style templates can be found at  <https://journals.plos.org/plosone/s/file?id=wjVg/PLOSOne_formatting_sample_main_body.pdf> and  <https://journals.plos.org/plosone/s/file?id=ba62/PLOSOne_formatting_sample_title_authors_affiliations.pdf> | Will do. |
| 2 | 2. Thank you for stating the following in the Acknowledgments Section of your manuscript:  “We want to thank the respondents for their participation in the surveys on which this presentation is based. We also want to thank Jørgen S. Petersen and Per Hvenegaard for information on cat registration in 1998. Also we owe thanks to Eliza Ruiz Izaguirre for assistance in search of literature and to Nicoline Skandov for assisting UG and BW with the data collection in 2022. Finally, thanks are due to Skibsreder Per Henriksen, R. og Hustrus Fond and Kitty og Viggo Freisleben Jensens Fond for economic support of the 2022 study and Animal Welfare Denmark and Aage V. Jensens Fonde for economic support of the 1998 study. The funders had no influence on the design of the study and the interpretation of the presented results.”  We note that you have provided funding information that is currently declared in your Funding Statement. However, funding information should not appear in the Acknowledgments section or other areas of your manuscript. We will only publish funding information present in the Funding Statement section of the online submission form.  Please remove any funding-related text from the manuscript and let us know how you would like to update your Funding Statement. Currently, your Funding Statement reads as follows:  “We received funding from Skibsreder Per Henriksen, R. og Hustrus Fond and Kitty og Viggo Freisleben Jensens Fond for the 2022 study and from Animal Welfare Denmark and Aage V. Jensens Fonde for the 1998 study. The funders had no influence on the design of the study and the interpretation of the presented results.”  Please include your amended statements within your cover letter; we will change the online submission form on your behalf. | We have removed funding-related information from the Acknowledgements Section. And we see no need to update our Funding Statement. |
| 3 | 3. Thank you for stating the following in the Competing Interests section:  “Marianne Lund Ujvári worked for Animal Protection Denmark while doing the first part of the project in the late 1990ies. The remaining authors have declared that no competing interests exist.”  Please confirm that this does not alter your adherence to all PLOS ONE policies on sharing data and materials, by including the following statement: "This does not alter our adherence to PLOS ONE policies on sharing data and materials.” (as detailed online in our guide for authors <http://journals.plos.org/plosone/s/competing-interests> ). If there are restrictions on sharing of data and/or materials, please state these. Please note that we cannot proceed with consideration of your article until this information has been declared.  Please include your updated Competing Interests statement in your cover letter; we will change the online submission form on your behalf. | We will add the statement to the Competing Interests section. |
| 4 | 4. We note that Figure S1 (Map Country cats paper.pdf ) in your submission contain [map/satellite] images which may be copyrighted. All PLOS content is published under the Creative Commons Attribution License (CC BY 4.0), which means that the manuscript, images, and Supporting Information files will be freely available online, and any third party is permitted to access, download, copy, distribute, and use these materials in any way, even commercially, with proper attribution. For these reasons, we cannot publish previously copyrighted maps or satellite images created using proprietary data, such as Google software (Google Maps, Street View, and Earth). For more information, see our copyright guidelines: http://journals.plos.org/plosone/s/licenses-and-copyright.  We require you to either (1) present written permission from the copyright holder to publish these figures specifically under the CC BY 4.0 license, or (2) remove the figures from your submission:  a. You may seek permission from the original copyright holder of Figure S1 to publish the content specifically under the CC BY 4.0 license.  We recommend that you contact the original copyright holder with the Content Permission Form (http://journals.plos.org/plosone/s/file?id=7c09/content-permission-form.pdf) and the following text:  “I request permission for the open-access journal PLOS ONE to publish XXX under the Creative Commons Attribution License (CCAL) CC BY 4.0 (http://creativecommons.org/licenses/by/4.0/). Please be aware that this license allows unrestricted use and distribution, even commercially, by third parties. Please reply and provide explicit written permission to publish XXX under a CC BY license and complete the attached form.”  Please upload the completed Content Permission Form or other proof of granted permissions as an ""Other"" file with your submission.  In the figure caption of the copyrighted figure, please include the following text: “Reprinted from [ref] under a CC BY license, with permission from [name of publisher], original copyright [original copyright year].”  b. If you are unable to obtain permission from the original copyright holder to publish these figures under the CC BY 4.0 license or if the copyright holder’s requirements are incompatible with the CC BY 4.0 license, please either i) remove the figure or ii) supply a replacement figure that complies with the CC BY 4.0 license. Please check copyright information on all replacement figures and update the figure caption with source information. If applicable, please specify in the figure caption text when a figure is similar but not identical to the original image and is therefore for illustrative purposes only.  The following resources for replacing copyrighted map figures may be helpful:  USGS National Map Viewer (public domain): <http://viewer.nationalmap.gov/viewer/>  The Gateway to Astronaut Photography of Earth (public domain): <http://eol.jsc.nasa.gov/sseop/clickmap/>  Maps at the CIA (public domain): <https://www.cia.gov/library/publications/the-world-factbook/index.html> and <https://www.cia.gov/library/publications/cia-maps-publications/index.html>  NASA Earth Observatory (public domain): <http://earthobservatory.nasa.gov/>  Landsat: <http://landsat.visibleearth.nasa.gov/>  USGS EROS (Earth Resources Observatory and Science (EROS) Center) (public domain): [http://eros.usgs.gov/#](http://eros.usgs.gov/)  Natural Earth (public domain): <http://www.naturalearthdata.com/> | The map is based on map data provided by The Danish Agency for Data Supply and Efficiency, Copenhagen NV, Denmark, and Danish Municipalities; GeoDanmark, <https://www.geodanmark.dk/home/vejledninger/vilkaar-for-data-anvendelse/> which grants users permission from the copyright holder to publish figures based on the data specifically under the CC BY 4.0 license.  This information has also been included in the ms. |
| 5 | 5. Please review your reference list to ensure that it is complete and correct. If you have cited papers that have been retracted, please include the rationale for doing so in the manuscript text, or remove these references and replace them with relevant current references. Any changes to the reference list should be mentioned in the rebuttal letter that accompanies your revised manuscript. If you need to cite a retracted article, indicate the article’s retracted status in the References list and also include a citation and full reference for the retraction notice. | We have reviewed the reference list carefully and have made the following changes:   - The entire reference list has been replaced, and each reference updated - Access-dates have been added for all references - Issue dates are removed - Month of publication removed so only the year is given - For the Ujvári-reference, the publisher (and city) has been added, along with a title in English - An internet-reference for Statistics Denmark, which was previously given in the text, has been moved to other references - All journal names are now abbreviated - The reference to the software R is now in the reference list |
| 6 | Additional Editor Comments:  Dear Authors  Kindly address all the concerns raised by the reviewers of this manuscript. | We have carefully tried to do so – see below. |

| **Nb.** | **Comments from Reviewer #1** | **Responses** |
| --- | --- | --- |
| 7 | Is the manuscript presented in an intelligible fashion and written in standard English?  PLOS ONE does not copyedit accepted manuscripts, so the language in submitted articles must be clear, correct, and unambiguous. Any typographical or grammatical errors should be corrected at revision, so please note any specific errors here.  Reviewer #1: No | The native English speaker among the authors, C.P., has done a careful check of the English. We have also made corrections at the one point in the pdf where the reviewer commented on the language. |
| 8 | Dear authors,  The paper is very interesting and important evidence for a shift in household cat keeping.  My suggestions are for your inspiration.  There are some minor issues to have a look at, according to me, this would help for clarifying some things I missed. | Thank you, a lot, for these kind words. And thank you for all the very useful comments that have allowed us to improve the manuscript in numerous ways. |
| 9 | - Terminology: be consistent in terminology: (1) euthanasia or killing, and if different define so. (2) marking of cats, defining in the beginning what you mean, would help the reader (3) the most important one: be consistent in your categories or defining of cats (household, owned, domestic, colonies,...)! (see suggestions in pdf) | Thanks. Re (1): we now consistently use the term “killing”; re (2): we now have explained in the start of the paper what we mean and have avoided the term “marking” and instead talk about “microchipping/earmarking”; re (3): we have made changes to make it more consistent and have followed all the good suggestions made in the pdf. |
| 10 | - Some more specific numbers and references to papers used would help the reader (see suggestions in pdf). | We have followed all the suggestions made about this in the pdf. |
| 11 | - Add the complete survey as an appendix, for me this is very important as a reader. | The survey has been translated into English and is now included as supporting information. |
| 12 | - Maybe a more precise title would help the reader from the onset of reading | We have changed the title in accordance with your suggestion. |
| 13 | - Management by owners and production of kittens seemed strangely (and not respectfully for me) worded. | We have reworded in accordance with your suggestions. |
| 14 | For me as a reader it would have been helpful if certain legislative changes (in relation to cats) or other societal changes in Denmark would have been described and may have helped in understanding the trend you described. I think there is no need to state (in lines 432-434) because of not using measures on attitudes, to try do give a hint what could be influential. ("Thirdly, there were no real measures of people’s attachment to 433 their cats and therefore efforts to explain the findings in terms of changes in attitudes to 434 owned cats are bound to be speculative.") Ofcourse this will be speculative, but there should be literature on this, that has been trying to find out, why cat keeping has changed over time. I would also be really interested into reading what could be important studies to help to find this out in Denmark. | Thank you for inviting us to speculate a bit more, which we are happy to do. We have added the following to the discussion: “A recent study comparing owner attachment to cats relative to dogs in three countries, Austria, Denmark and the United Kingdom (34), shows large cross-country variation. Owners in Denmark cared less about their cats, in comparison to their dogs, than in the two other countries, Austria and the UK. This indicates that attachment to cats is not static but is developing and even though attachment to cats is lower in Denmark compared to other countries it most likely has increased over time. This increased attachment to cats and the related concern about cat welfare would also help to explain the relatively small population of stray and feral cats found in a recent study (28).  There have been two potentially relevant changes to Danish legislation regarding cats during the 24 year period between the two studies at issue here. Both changes apply to the law regulating confinement of animals on an owner’s property, a law mainly focused on farm animals, but also covering cats. A 2014 change in the law banned the shooting of stray cats (35,36); and a 2021 change in the law meant that ownership can’t be claimed of cats that lack earmarks or microchips (37). Both changes reflect the increasing perceived value of cats as companion animals." |
| 15 | I wish you a good publication, | Thank you so much. |

| **Nb.** | **Comments from Reviewer #2** | **Responses** |
| --- | --- | --- |
| 16 | A great read and a welcome consideration of changing trends in cat ownership. I would welcome a version of this manuscript in publication. | Thank you for the kind words – much appreciated. |
| 17 | I was unable to review the questionnaires as they were not available in English. Apologies, it may be that some of my questions would have been addressed by having access to these, if this is the case, please take my comments to indicate further information in the main text would be helpful. The version of the questionnaire translated into English appended as supplementary material as indicated L181 would be helpful. | A version of the 2022 questionnaire translated into English has now been appended. |
| 18 | Also, my main comments are around improving the clarity of the manuscript specifically around cat terminology used throughout. | Thank you for these comments that have certainly served to clarify the text. |
| 19 | Abstract and throughout- no definition of what a “full-scale” farm is compared to a “hobby” farm. Please clarify for the reader | It has now been explained that a full-scale farm is one from which a farmer makes a living. |
| 20 | Marked and registered is used throughout but unclear of exactly what this means- assume microchipping, while wearing a collar is marking but not registering. So it’s important to define what actually is being compared. Also along these lines: • L86 says “other identifying marks” and I am unsure what this means. • Terminology used inconsistently throughout the manuscript there are instances of “marked and registered”, “marked or registered”, “marked/registered” and “marked and/or registered”. These could all mean slightly different things can this be clearly defined and used consistently throughout. Especially in results where it is unclear whether like for like scenarios are being compared e.g. L321 “in 1998 had a cat that was marked and registered” and for 2022 L325 “On properties where cats had indoor access 78% (95/122) answered 326 that cats were marked or registered” So you would expect different % as the text indicates different things are been considered in 1998 and 2022. | Thank you so much for pointing out these confusing unclarities and inconsistencies.  We have now throughout explained that the cats in question are cats that either have been micro-chipped or have a tattooed earmark and that registered means that owner information is to be found in one of the two national cat registries; and we have streamlined the terminology. Also, we have made clear that in both cases we are looking at cats which are both microchipped/earmarked and registered. There was some inconsistency when it came to comparison with national data. While the data for the area in 1998 and 2022 both concerned cats that were microchipped/earmarked and registered, the national data used a broader definition. This has now been adjusted so that we compare with national data only for cats which are both microchipped/earmarked and registered, and the revised numbers reflect this. Accordingly, we amended note ** in Table 5, so it is reported that only ”, “Ear marked and registered” and “Chip marked and registered“ cats are included. Also, the prevalences reported in Table 5 are slightly reduced, as are the reporting of these prevalences in the last paragraph of the result section. |
| 21 | The first paragraph of the introduction is largely repetitive of the abstract/conclusion. Suggest remove. | It has been removed. |
| 22 | L17 and throughout the authors use “over a 24 year time period” this implies cat populations were followed during this period. Suggest rephrasing for clarity e.g. following a 24 year time period or pre and post a 24 year time period. | Thank you for pointing out this unclear formulation. We now both in the Abstract and in the Intro say that we compare “at two points in time separated by 24 years”. |
| 23 | The cat terminology used is confusing and ambiguity in terminology is a potential limitation that should be mentioned in the discussion. E.g. • “barn cats” are mentioned but not defined (e.g. L131, L393, L413) and unclear whether these are pet cats or all cats in rural areas? Be good to compare to how cats are defined in the international literature • The authors categorise by access to indoors and not access to indoors, could the latter category include cats not considered owned? • Does the questionnaire ask about all cats, i.e. pet, farm, feral may be thought of as different so if only considering owned cats that does not mean a drop in cat numbers • Also, there is no consideration of indoor-only cats. | In the questionnaire clearly indicate that we only consider cats that the respondents view as owned. Among these we distinguish between those with indoor access and those without indoor access. We are aware that the term “barn cats” is sometimes in the literature used more broadly also to include cats that are not considered owned. We now only refer to “barn cats” only once in research question 2) which now reads: “2) How has owner behaviour in terms of owned cats with indoor access vs. cats with outdoor access only (these include cats usually called barn cats that have access to farm buildings but not residential homes) changed?”  There is no explicit consideration of indoor-only cats. From what we already know about the low prevalence of indoor-only cats in Denmark there will be very few, if any, of them in a rural area like the one here studied. They will anyway be included in the group of cats with indoor access. |
| 24 | Unclear in the methods the purpose of the information letter (L155) were these completed and returned by residents or are the returns mentioned due to the postal service being unable to deliver? | The returns mentioned are due to the postal service being unable to deliver. This has now been made clear in the text. |
| 25 | L257 “the weight variable was not activated” why? this is a bit unclear, could this potentially be a limitation to mention? | It is an unresolved discussion whether it is appropriate to use weighed data beyond the reporting of descriptive results (means, proportions etc). We will not attempt to resolve that discussion in this paper.  But we have now in the methods section added that the results are similar irrespective of whether the data are weighed or not. xt has now been clarified here |
| 26 | The Discussion is reported in an unusual way under a series of question headings, also it is largely repetitive of the results, Here I would expect to find prose and wider implications, bringing findings together to paint an overall picture, limitations, suggestions for future studies and certainly a conclusion. | We have changed the form and structure of the Discussion to accommodate this comment. And we agree that it now reads better. |
| 27 | L25 “significant drop in the proportion of cats without indoor access” This double negative is confusing, suggest rephrase e.g. increase in cats with indoor access | Good point – has been changed in line with your suggestion. |
| 28 | L89 Microchipping is not legal in all countries of the UK, just England, suggest reword | We have reworded so that the text only says that there is a legal requirement for microchipping in England. |
| 29 | L124 “In addition, the study population was compared to the target population of rural areas” unclear on what this means? Target population from the national cat registers? | No, the concern here is whether the areas are typically rural. We have tried to change the wording to make that clear. |
| 30 | L128 introduce the scientific name at point of first use and also as a domestic cat. | Done – thank you. |
| 31 | Couldn’t see any explanatory caption for Figure S1 | Has now been added. |
| 32 | L208 and L257 “p-value of 0.05”, should be below 0.05 | Done. |
| 33 | L241-242 specifies a population density but no unit of area is provided, please clarify. | This has now been specified in the Methods section. |
| 34 | L371 website should be referenced appropriately with access date | This and other references for websites have been supplemented with access dates and listed as specified in the author submission guidelines (see also Comment 5) |
| 35 | L380 typo surplus. Also, unclear of the definition of surplus, would not euthanised in some circumstances be surplus or is this known to be on health grounds? | We have now defined “surplus” kittens as those unaccounted for (and not belonging to the groups “kept” and “killed”). |
| 36 | L398 killing is population control not specifically reproduction | We have changed to “population control”. |
| 37 | L446 “However, overall the limitations are not seen as major.” This is subjective and should be removed and as mentioned previously a conclusion paragraph would be great. | Sentence has been removed. Thanks for the many precise and perceptive comments that certainly have helped to improve the paper. |
