# Supplementary material for: Changes in management of owned cats in the countryside – A comparison of results from surveys undertaken in the same rural area of Denmark in 1998 and 2022
Source: PLoS One. 2025 Feb 19;20(2):e0316704. doi: 10.1371/journal.pone.0316704 (PMC11838870; doi:10.1371/journal.pone.0316704)
Supplement: Attachment — Submitted filename: Rebuttal letter.docx [file pone.0316704.s009.docx]

**Rebuttal letter**

PONE-D-24-41843R1

*We received the following single comment:*

Reviewer #1: Dear authors,
I want to click "accept," but I held off due to one final, really minor but important concern regarding the language. Please review the text to ensure it is polished and suitable for publication—for example, replacing contractions like "don't" with "do not."
Thank you for your interesting study! All the clarifications and added material were very useful for me.
Best regards

*Our response:*

Thank you for the kind words and all the good comments that have helped us improve our paper a lot. Now the text has been carefully reviewed by the native English speaker among the authors, and among other things all contractions have been replaced.
